# Supplementary material for: Re-assembly, quality evaluation, and annotation of 678 microbial eukaryotic reference transcriptomes
Source: Gigascience. 2018 Dec 13;8(4):giy158. doi: 10.1093/gigascience/giy158 (PMC6481552; doi:10.1093/gigascience/giy158)
Supplement: GIGA-D-18-00191_Original-Submission.pdf [file giy158_giga-d-18-00191_original-submission.pdf]

GigaScience

Re-assembly, quality evaluation, and annotation of 678 microbial eukaryotic reference transcriptomes

--Manuscript Draft--

**Manuscript Number:**	GIGA-D-18-00191
**Full Title:**	Re-assembly, quality evaluation, and annotation of 678 microbial eukaryotic reference transcriptomes
**Article Type:**	Research
**Funding Information:**	Gordon and Betty Moore Foundation (GBMF4551) — Dr. C. Titus Brown
**Abstract:**	Background: De novo transcriptome assemblies are required prior to analyzing RNAseq data from a species without an existing reference genome or transcriptome. Despite the prevalence of transcriptomic studies, the effects of using different workflows, or 'pipelines', on the resulting assemblies are poorly understood. Here, a pipeline was programmatically automated and used to assemble and annotate raw transcriptomic short read data collected by the Marine Microbial Eukaryotic Transcriptome Sequencing Project (MMETSP). Transcriptome assemblies generated through this pipeline were evaluated and compared against assemblies that were previously generated with a pipeline developed by the National Center for Genome Research (NCGR). Findings: New transcriptome assemblies contained 70% of the previous contigs as well as new content. On average, 7.8% of the annotated contigs in the new assemblies were novel gene names not found in the previous assemblies. Taxonomic trends were observed in the assembly metrics, with assemblies from the Dinoflagellata and Ciliophora phyla showing a higher percentage of open reading frames and number of contigs than transcriptomes from other phyla. Conclusions: Given current bioinformatics approaches, there is no single 'best' reference transcriptome for a particular set of raw data. As the optimum transcriptome is a moving target, improving (or not) with new tools and approaches, automated and programmable pipelines are invaluable for managing the computationally-intensive tasks required for re-processing large sets of samples with revised pipelines. Moreover, automated and programmable pipelines facilitate the comparison of diverse sets of data by ensuring a common evaluation workflow was applied to all samples. Thus, re-assembling existing data with new tools using automated and programmable pipelines may yield more accurate identification of taxon-specific trends across samples in addition to novel and useful products for the community.
**Corresponding Author:**	C. Titus Brown<br>UC Davis<br>Davis, CA UNITED STATES
**Corresponding Author Secondary Information:**	
**Corresponding Author's Institution:**	UC Davis
**Corresponding Author's Secondary Institution:**	
**First Author:**	Lisa Kristine Johnson
**First Author Secondary Information:**	
**Order of Authors:**	Lisa Kristine Johnson
	Harriet Alexander
	C. Titus Brown
**Order of Authors Secondary Information:**	
**Additional Information:**	
**Question**	**Response**

Powered by Editorial Manager® and ProduXion Manager® from Aries Systems Corporation

Are you submitting this manuscript to a special series or article collection?	No
**Experimental design and statistics**<br><br>Full details of the experimental design and statistical methods used should be given in the Methods section, as detailed in our Minimum Standards Reporting Checklist. Information essential to interpreting the data presented should be made available in the figure legends.<br><br>Have you included all the information requested in your manuscript?	Yes
**Resources**<br><br>A description of all resources used, including antibodies, cell lines, animals and software tools, with enough information to allow them to be uniquely identified, should be included in the Methods section. Authors are strongly encouraged to cite Research Resource Identifiers (RRIDs) for antibodies, model organisms and tools, where possible.<br><br>Have you included the information requested as detailed in our Minimum Standards Reporting Checklist?	Yes
**Availability of data and materials**<br><br>All datasets and code on which the conclusions of the paper rely must be either included in your submission or deposited in publicly available repositories (where available and ethically appropriate), referencing such data using a unique identifier in the references and in the "Availability of Data and Materials" section of your manuscript.<br><br>Have you have met the above requirement as detailed in our Minimum Standards Reporting Checklist?	Yes

Powered by Editorial Manager® and ProduXion Manager® from Aries Systems Corporation

Click here to access/download;Manuscript;msfile-1.pdf
Click here to view linked References

Re-assembly, quality evaluation, and annotation of 678 microbial eukaryotic reference transcriptomes

Lisa K. Johnson[1,2], Harriet Alexander[1], C. Titus Brown[1,2,3]*

[1] Department of Population Health and Reproduction, School of Veterinary Medicine, University of California, Davis
[2] Molecular, Cellular, and Integrative Physiology Graduate Group, University of California, Davis
[3] Genome Center, University of California, Davis
* Correspondence: ctbrown@ucdavis.edu

Abstract

Background

De novo transcriptome assemblies are required prior to analyzing RNAseq data from a species without an existing reference genome or transcriptome. Despite the prevalence of transcriptomic studies, the effects of using different workflows, or “pipelines”, on the resulting assemblies are poorly understood. Here, a pipeline was programmatically automated and used to assemble and annotate raw transcriptomic short read data collected by the Marine Microbial Eukaryotic Transcriptome Sequencing Project (MMETSP). Transcriptome assemblies generated through this pipeline were evaluated and compared against assemblies that were previously generated with a pipeline developed by the National Center for Genome Research (NCGR).

Findings

New transcriptome assemblies contained 70% of the previous contigs as well as new content. On average, 7.8 ± 0.19% of the annotated contigs in the new assemblies were novel gene names not found in the previous assemblies. Taxonomic trends were observed in the assembly metrics, with assemblies from the Dinoflagellata and Ciliophora phyla showing a higher percentage of open reading frames and number of contigs than transcriptomes from other phyla.

Conclusions

Given current bioinformatics approaches, there is no single ‘best’ reference transcriptome for a particular set of raw data. As the optimum transcriptome is a moving target, improving (or not) with new tools and approaches, automated and programmable pipelines are invaluable for managing the computationally-intensive tasks required for re-processing large sets of samples with revised pipelines. Moreover, automated and programmable pipelines facilitate the comparison of diverse sets of data by ensuring a common evaluation workflow was applied to all samples. Thus, re-assembling existing data with new tools using automated and programmable pipelines may yield more accurate identification of taxon-specific trends across samples in addition to novel and useful products for the community.

Introduction

The analysis of gene expression from high-throughput nucleic acid sequence data relies on the presence of a high quality reference genome or transcriptome. When there is no reference genome or transcriptome for an organism of interest, raw RNA sequence data (RNAseq) must be assembled *de novo* into a transcriptome [1]. This type of analysis is ubiquitous across many fields. For example, evolutionary developmental biology [2], cancer biology [3], agriculture [4,5], ecological physiology [6,7], and biological oceanography [8]. In recent years, substantial investments have been made in data generation, primary data analysis, and development of downstream applications, such as biomarkers and diagnostic tools [9–16].

Methods for *de novo* RNAseq assembly of the most common short read Illumina sequencing data continue to evolve rapidly, especially for non-model species [17]. At this time, there are several major *de novo* transcriptome assembly software tools available to choose from, including Trinity [18], SOAPdenovo-Trans [19], Trans-ABySS [20], Oases [21], SPAdes [22], IDBA-tran [23], and Shannon [24]. The availability of these options stems from continued research into the unique computational challenges associated with transcriptome assembly of short read Illumina RNAseq data, including large memory requirements, alternative splicing and allelic variants [18,25].

The continuous development of new tools and workflows for RNAseq analysis combined with the vast amount of publicly available RNAseq data [26] raises the opportunity to re-analyze existing data with new tools. This, however, is rarely done systematically. To evaluate the performance impact of new tools on old data, we developed and applied a programmatically automated *de novo* transcriptome assembly workflow that is modularized and extensible based on the Eel Pond Protocol [27]. This workflow incorporates Trimmomatic [28], digital normalization with khmer software [29,30], and the Trinity *de novo* transcriptome assembler [18].

To evaluate this pipeline, we re-analyzed RNAseq data from 678 samples generated as part of the Marine Microbial Eukaryotic Transcriptome Sequencing Project (MMETSP). The MMETSP RNAseq data set was generated to broaden the diversity of sequenced marine protists to enhance our understanding of their evolution and roles in marine ecosystems and biogeochemical cycles [31,32]. With data from species spanning more than 40 eukaryotic phyla, the MMETSP provides one of the largest publicly-available collections of RNAseq data from a diversity of species. Moreover, the MMETSP used a standardized library preparation procedure and all of the samples were sequenced at the same facility, making this data set unusually comparable.

Reference transcriptomes for the MMETSP were originally assembled by the National Center for Genome Research (NCGR) with a pipeline which used the Trans-ABySS software program [31] to assemble the short reads. The transcriptomes generated from the NCGR pipeline have already facilitated discoveries in the evolutionary history of ecologically significant genes [33,34], differential gene expression under shifting environmental conditions [8,35], inter-group transcriptome comparisons [36], unique transcriptional features [37–39], and meta-transcriptomic studies [34–36].

In re-assembling the MMETSP data, we sought to compare and improve the original MMETSP reference transcriptome and to create a platform which facilitates automated re-assembly and evaluation. Here, we show that our re-assemblies had higher evaluation metrics and contained most of the NCGR contigs as well as adding new content.

Methods

Programmatically Automated Pipeline

An automated pipeline was developed to execute the steps of the Eel Pond mRNAseq Protocol [27], a lightweight protocol for assembling short Illumina RNA-seq reads that uses the Trinity *de novo* transcriptome assembler. This protocol generates *de novo* transcriptome assemblies of acceptable quality [43]. The pipeline was used to assemble all of the data from the MMETSP (Figure 1). The code and instructions for running the pipeline are available at https://doi.org/10.5281/zenodo.249982.

The steps of the pipeline applied to the MMETSP are as follows:

1. Download the raw data

Raw RNA-seq data sets were obtained from the National Center for Biotechnology Information (NCBI) Sequence Read Archive (SRA) from BioProject PRJNA231566. Data were paired-end (PE) Illumina reads with lengths of 50 bases for each read. A metadata (SraRunInfo.csv) file obtained from the SRA web interface was used to provide a list of samples to the *get_data.py* pipeline script, which was then used to download and extract fastq files from 719 records. The script uses the fastq-dump program from the SRA Toolkit to extract the SRA-formatted fastq files (version 2.5.4) [44]. There were 18 MMETSP samples with more than one SRA record (MMETSP0693, MMETSP1019, MMETSP0923, MMETSP0008, MMETSP1002, MMETSP1325, MMETSP1018, MMETSP1346, MMETSP0088, MMETSP0092, MMETSP0717, MMETSP0223, MMETSP0115, MMETSP0196, MMETSP0197, MMETSP0398, MMETSP0399, MMETSP0922). In these cases, reads from multiple SRA records were concatenated together per sample. Taking these redundancies into consideration, there were a total of 678 re-assemblies generated from the 719 records in PRJNA231566 (Supplemental Notebook 1). Assembly evaluation metrics were not calculated for MMETSP samples with more than one SRA record because these assemblies were different than the others, containing multiple samples, and thus not as comparable.

Initial transcriptomes that were assembled by the National Center for Genome Resources (NCGR), using methods and data described in the original publication [31], were downloaded from the iMicrobe repository to compare with our re-assemblies (ftp://ftp.imicrobe.us/projects/104/). There were two versions of each assembly, 'nt' and 'cds'. The version used for comparison is noted below in each evaluation step. To our knowledge, the NCGR took extra post-processing steps to filter content, leaving only coding sequences in the 'cds' versions of each assembly [31].

2. Perform quality control

Reads were analyzed with FastQC (version 0.11.5) and multiqc (version 1.2) [45] to confirm overall qualities before and after trimming. A conservative trimming approach [46] was used with Trimmomatic (version 0.33) [28] to remove residual Illumina adapters and cut bases off the start (LEADING) and end (TRAILING) of reads if they were below a threshold Phred quality score (Q<2).

3. Apply digital normalization

To decrease the memory requirements for each assembly, reads were interleaved, normalized to a *k*-mer ($k = 20$) coverage of 20 and a memory size of 4e9, then low-abundance *k*-mers from reads with a coverage above 18 were trimmed. Orphaned reads, where the mated pair was removed during normalization, were included in the assembly.

4. Assemble

Transcriptomes were assembled from normalized reads with Trinity 2.2.0 using default parameters ($k = 25$).

The resulting assemblies are referred to below as the “Lab for Data Intensive Biology” assemblies, or DIB assemblies. The original assemblies are referred to as the NCGR assemblies.

5. Post-assembly assessment

Transcriptomes were annotated using the dammit pipeline (Scott 2016), which relies on the following databases as evidence: Pfam-A [47], Rfam [48], OrthoDB [49]. In the case where there were multiple database hits, one gene name per contig was selected by choosing the name of the lowest e-value match (<1e-05).

All assemblies were evaluated using metrics generated by the Transrate program [50]. Trimmed reads were used to calculate a Transrate score for each assembly, which represents the geometric mean of all contig scores multiplied by the proportion of input reads providing positive support for the assembly [50]. Comparative metrics were calculated using Transrate for each MMETSP sample between DIB and the NCGR assemblies using the Conditional Reciprocal Best BLAST hits (CRBB) algorithm [51]. A forward comparison was made with the NCGR assembly used as the reference and each DIB re-assembly as the query. Reverse comparative metrics were calculated with each DIB re-assembly as the reference and the NCGR assembly as the query. Transrate scores were calculated for each assembly using the Trimmomatic quality-trimmed reads, prior to digital normalization.

Benchmarking Universal Single-Copy Orthologs (BUSCO) software (version 3) was used with a database of 234 orthologous genes specific to protistans and 306 genes specific to eukaryota with open reading frames in the assemblies. BUSCO scores are frequently used as one measure of assembly completeness [52].

To assess the occurrences of fixed-length words in the assemblies, unique 25-mers were measured in each assembly using the HyperLogLog estimator of cardinality built into the khmer software package [53].

Unique gene names were compared from a random subset of 296 samples using the dammit annotation pipeline [54]. If a gene name was annotated in NCGR but not in DIB, this was considered a gene uniquely annotated in NCGR. Unique gene names were normalized to the total number of annotated genes in each assembly.

A Tukey's honest significant different (HSD) post-hoc range test of multiple pairwise comparisons was used in conjunction with an ANOVA to measure differences between distributions of data from the top eight most-represented phyla ("Bacillariophyta", "Dinophyta", "Ochrophyta", "Haptophyta", "Ciliophora", "Chlorophyta", "Cryptophyta", "Others") using the 'agricolae' package version 1.2-8 in R version 3.4.2 (2017-09-28). Margins sharing a letter in the group label are not significantly different at the 5% level (Figure 8). Averages are reported ± standard deviation.

Results

After assemblies and annotations were completed, files were uploaded to Figshare and Zenodo are available for download [55,56]. Due to obstacles encountered uploading and maintaining 678 assemblies on Figshare, Zenodo will be the long-term archive for these re-assemblies http://doi.org/10.5281/zenodo.1212585.

Differences in available evaluation metrics between NCGR and DIB were variable.

The majority of transcriptome evaluation metrics collected for each sample were higher in Trinity-based DIB re-assemblies than for the Trans-ABySS-based NCGR assemblies (Table 1), with the exception being the Transrate score from the "nt" version of the assembly. The Transrate score with this 'cds' version was higher in DIB compared to NCGR but lower in DIB compared to the NCGR 'nt' version (Supplemental Figure 1).

The DIB re-assemblies had more contigs than the NCGR assemblies in 83.5% of the samples (Table 1). The mean number of contigs in the DIB re-assemblies was 48,361 ± 35,703 while the mean number of contigs in the NCGR 'nt' assemblies was 30,532 ± 21,353 (Figure 2). A two-sample Kolmogorov-Smirnov test comparing distributions indicated that the number of contigs were significantly different between DIB and NCGR assemblies ($p < 0.001$, D = 0.35715). Transrate scores [35], which calculate the overall quality of the assembly based on the original reads, were significantly higher in the DIB re-assemblies (0.31 ± 0.1) compared to the 'cds' versions of the NCGR assemblies (0.22 ± 0.09) ($p < 0.001$, D = 0.49899). The Transrate scores in the NCGR 'nt' assemblies (0.35 ± 0.09) were significantly higher than the DIB assemblies (0.22 ± 0.09) ($p < 0.001$, D = 0.22475) (Supplemental Figure 1). The frequency of the differences between Transrate scores in the NCGR 'nt' assemblies and the DIB re-assemblies appears to be normally distributed (Figure 2C). Transrate scores from the DIB assemblies relative to the NCGR 'nt' assemblies did not appear to have taxonomic trends (Supplemental Figure 2).

The DIB re-assemblies contained most of the NCGR contigs as well as new content.

We applied CRBB to evaluate overlap between the assemblies. A positive CRBB result indicates that one assembly contains the same contig information as the other. Thus, the proportion of positive CRBB hits can be used as a scoring metric to compare the relative similarity of content between two assemblies. For example, MMETSP0949 (*Chattonella subsalsa*) had 39,051 contigs and a CRBB score of 0.71 in the DIB re-assembly whereas in the NCGR assembly of the same sample had 18,873 contigs and a CRBB score of 0.34. This indicated that 71% of the reference of DIB was covered by the NCGR assembly, whereas in the reverse alignment, the NCGR reference assembly was only covered by 34% of the DIB re-assembly. The mean CRBB score in DIB when queried against NCGR 'nt' as a reference was 0.70 ± 0.22, while the mean proportion for NCGR 'nt' assemblies queried against DIB re-assemblies was 0.49 ± 0.10 ($p < 0.001$, D = 0.71121) (Figure 3). This indicates that more content from the NCGR assemblies was included in the DIB re-assemblies than vice versa and also suggests that the DIB re-assemblies overall have additional content. This finding is reinforced by higher unique *k*-mer content found in the DIB re-assemblies compared to NCGR, where more than 95% of the samples had more unique *k*-mers in the DIB re-assemblies compared to NCGR assemblies (Figure 4).

To investigate whether the new sequence content was genuine, we examined two different metrics that take into account the biological quality of the assemblies. First, the estimated content of open reading frames (ORFs), or coding regions, across contigs was quantified. Though DIB re-assemblies had more contigs, the ORF content is similar to the original assemblies, with a mean of 81.8 ± 9.9% ORF content in DIB re-assemblies and 76.7 ± 10.1% ORF content in the NCGR assemblies. Nonetheless, ORF content in DIB re-assemblies was slightly higher than NCGR assemblies for 95% of the samples (Figure 5 A,B), although DIB re-assemblies had significantly higher ORF content ($p < 0.001$, D = 2681). Secondly, when the assemblies were queried against the eukaryotic BUSCO database [37], the percentages of BUSCO eukaryotic matches in the DIB re-assemblies (63 ± 18.6%) were less significantly different compared to the original NCGR assemblies (65 ± 19.1%) ($p = 0.001873$, D = 0.10291) (Figure 5 C,D). Thus, although the number of contigs and amount of content was increased in the DIB re-assemblies compared to the NCGR assemblies, the ORF content and contigs matching with the BUSCO eukaryotic (Figure 5 C,D) and protistan (Supplemental Figure 3) databases did not decrease, suggesting that the extra content contained similar proportions of ORFs and BUSCO annotations and, therefore, might be biologically meaningful.

Following annotation by the dammit pipeline (Scott 2016), 91 ± 1.6% of the contigs in the DIB re-assemblies had positive matches with sequence content in the databases queried (Pfam, Rfam, and OrthoDB), with 48 ± 0.9% of those containing unique gene names (the remaining are fragments of the same gene). Of those annotations, 7.8 ± 0.2% were identified as novel compared to the NCGR 'nt' assemblies, determined by a "false" CRBB result (Figure 6). Additionally, the number of unique gene names in DIB re-assemblies were higher in 97% of the samples compared to NCGR assemblies, suggesting an increase in genic content (Figure 7).

Novel contigs in the DIB re-assemblies likely represent a combination of unique annotations, allelic variants and alternatively spliced isoforms. For example, "F0XV46_GROCL",

"Helicase_C", "ODR4-like","PsaA_PsaB", and "Metazoa_SRP" are novel gene names found annotated in the DIB re-assembly of the sample MMETSP1473 (*Stichococcus* sp.) that were absent in the NCGR assembly of this same sample. Other gene names, for example "Pkinase_Tyr","Bromodomain", and "DnaJ", are found in both the NCGR and DIB assemblies, but are identified as novel contigs based on negative CRBB results in the DIB re-assembly of sample MMETSP1473 compared to the NCGR reference.

Assembly metrics varied by taxonomic group being assembled.

To examine systematic taxonomic differences in the assemblies, metrics for content and assembly quality were assessed (Figure 8). Metrics were grouped by the top eight most represented phyla in the MMETSP data set as follows: Bacillariophyta (N=173), Dinophyta (N=114), Ochrophyta (N=73), Chlorophyta (N=62), Haptophyta (N=61), Ciliophora (N=25), Cryptophyta (N=22) and Others (N=130).

While there were no major differences between the phyla in the number of input reads (Figure 8 A), the Dinoflagellates (Dinophyta) had significantly different (higher) contigs ($p < 0.01$), unique *k*-mers ($p < 0.001$), and % ORF ($p < 0.001$) compared to than other groups (Figure 8 B,C,D), and assemblies from Ciliates (Ciliophora) had lower % ORF ($p < 0.001$) (Figure 8 D).

Discussion

DIB re-assemblies contained the majority of the previously-assembled contigs.

We used a different pipeline than the original one used to create the NCGR assemblies, in part because new software was available [8] and in part because of new trimming guidelines [27]. We had no *a priori* expectation for the similarity of the results, yet we found that in the majority of cases the new DIB re-assemblies included substantial portions of the previous NCGR assemblies. Moreover, both the fraction of contigs with ORFs and the mean percentage of BUSCO matches were similar between the two assemblies, suggesting that both pipelines yielded equally valid contigs, even though the NCGR assemblies were less sensitive.

Reassembly with new tools can yield new results.

Evaluation with quality metrics suggested that the DIB re-assemblies were more inclusive than the NCGR assemblies. The Transrate scores in the DIB re-assemblies compared to the NCGR ‘nt’ assemblies were significantly lower, indicating that the NCGR ‘nt’ assemblies had better overall read inclusion in the assembled contigs whereas the DIB assemblies had higher Transrate scores than the NCGR ‘cds’ version. This suggests that the NCGR ‘cds’ version, which was post-processed to only include coding sequence content, was missing information originally in the quality-trimmed reads. The Transrate score [50] is one of the few metrics available for evaluating the ‘quality’ of a *de novo* transcriptome. It is similar to the DETONATE RSEM-EVAL score in that it returns a metric indicating how well the assembly is supported by the read data [57]. Metrics directly evaluating the underlying de Bruijn graph data structure used to produce the assembled contigs may be better evaluators of assembly quality in the future. Here, the DIB re-assemblies, which used the Trinity *de novo* assembly software, typically contained more *k*-mers, more annotated transcripts, and more unique gene names than the NCGR

assemblies. These points all suggest that additional content in these re-assemblies might be biologically meaningful and that these re-assemblies provide new content not available in the previous NCGR assemblies. Since contigs are probabilistic predictions made by assembly software for full-length transcripts [57], 'final' reference assemblies are approximations of the full set of transcripts in the transcriptome. Results from this study suggest that achieving the 'ideal' reference transcriptome is like chasing a moving target and that these predictions may continue to improve given updated tools in the future.

The evaluation metrics described here serve as a framework for better contextualizing the quality of protistan transcriptomes. For some species and strains in the MMETSP data set, these data represent the first nucleic acid sequence information available [31].

Automated and programmable pipelines can be used to process arbitrarily many RNAseq samples.

The automated and programmable nature of this pipeline was useful for processing large data sets like the MMETSP as it allowed for batch processing of the entire collection, including re-analysis when new tools or new samples become available (see op-ed Alexander et al. 2018). During the course of this project, we ran four re-assemblies of the MMETSP data set as versions of the component tools were updated. Each re-analysis required only a single command and approximately half a CPU-year of compute. New Trinity versions were released (Supplemental Notebook 2) The value of programmable automation is clear when new data sets become available, tools are updated, or many tools are compared in benchmark studies. Despite this, few assembly efforts completely automate their process, perhaps because the up-front cost of doing so is high compared to the size of the dataset typically being analyzed.

Analyzing many samples using a common pipeline identifies taxon-specific trends.

The MMETSP dataset presents an opportunity to examine transcriptome qualities for hundreds of taxonomically diverse species spanning a wide array of protistan lineages. This is among the largest set of diverse RNAseq data to be sequenced. In comparison, the Assemblathon2 project compared genome assembly pipelines using data from three vertebrate species [59]. The BUSCO paper assessed 70 genomes and 96 transcriptomes representing groups of diverse species (vertebrates, arthropods, other metazoans, fungi) [52]. Other benchmarking studies have examined transcriptome qualities for samples representing dozens of species from different taxonomic groupings [57,58]. A study with a more restricted evolutionary analysis of 15 plant and animals species [58] found no evidence of taxonomic trend in assembly quality but did find evidence of differences between assembly software packages [58].

With the MMETSP data set, we show that comparison of assembly evaluation metrics across this diversity provides not only a baseline for assembly performance, but also highlights particular metrics which are unique within some taxonomic groups. For example, the phyla Ciliophora had a significantly lower percentage of ORFs compared to other phyla. This is supported by recent work which has found that ciliates have an alternative triplet codon dictionary, with codons normally encoding STOP serving a different purpose [37–39], thus application of typical ORF finding tools fail to identify ORFs accurately in Ciliophora. Additionally, Dinophyta data sets had a significantly higher number of unique *k*-mers and total contigs in assemblies compared to

the assemblies from other data sets, despite having the same number of input reads. Such a finding supports previous evidence from studies showing that large gene families are constitutively expressed in Dinophyta [60].

In future development of *de novo* transcriptome assembly software, the incorporation of phylum-specific information may be useful in improving the overall quality of assemblies for different taxa. Phylogenetic trends are important to consider in the assessment of transcriptome quality, given that the assemblies from Dinophyta and Ciliophora are distinguished from other assemblies by some metrics. Applying domain-specific knowledge, such as specialized transcriptional features in a given phyla, in combination with other evaluation metrics can help to evaluate whether a transcriptome is of good quality or "finished" enough to serve as a high quality reference to answer the biological questions of interest.

Conclusion

As the rate of sequencing data generation continues to increase, efforts to programmatically automate the processing and evaluation of sequence data will become increasingly important. Ultimately, the goal in generating *de novo* transcriptomes is to create the best possible reference against which downstream analyses can be accurately based. This study demonstrated that re-analysis of old data with new tools and methods improved the quality of the reference assembly through an expansion of the gene catalogue of the dataset. Notably, these improvements arose without further experimentation or sequencing.

With the growing volume of nucleic acid data in centralized and de-centralized repositories, streamlining methods into pipelines will not only enhance the reproducibility of future analyses, but will facilitate inter-comparisons amongst datasets from similar and diverse. Automation tools were key in successfully processing and analyzing this large collection of 678 samples.

Acknowledgements

Camille Scott, Luiz Irber, Daniel Standage, and other members of the Data Intensive Biology lab at UC Davis provided helpful assistance with troubleshooting the assembly, annotation and evaluation pipeline. Funding was provided from the Gordon and Betty Moore Foundation under award number GBMF4551 to CTB. Scripts were tested and run on the MSU HPCC and NSF-XSEDE Jetstream cloud platform with allocation TG-BIO160028.

References

1. Geniza M, Jaiswal P. Tools for building *de novo* transcriptome assembly. Curr. Plant Biol. 2017;11–12:41–5.
2. Tulin S, Aguiar D, Istrail S, Smith J. A quantitative reference transcriptome for *Nematostella vectensis* early embryonic development: A pipeline for *de novo* assembly in emerging model systems. Evodevo. 2013;4.
3. Mittal VK, McDonald JF. De novo assembly and characterization of breast cancer transcriptomes identifies large numbers of novel fusion-gene transcripts of potential functional significance. BMC Med. Genomics. 2017;10:53.
4. Yang S, Liu H-D, Qiang Z, Zhang H-J, Zhi-Dong Z, Li Y-D, et al. ScienceDirect High-throughput sequencing of highbush blueberry transcriptome and analysis of basic helix-loop-helix transcription factors. J. Integr. Agric. 2017;16:591–604.
5. Suárez-Vega A, Gutiérrez-Gil B, Klopp C, Tosser-Klopp G, Arranz J-J. Comprehensive RNA-Seq profiling to evaluate lactating sheep mammary gland transcriptome. Sci. Data. 2016;3:160051.
6. Carruthers M, Yurchenko AA, Augley JJ, Adams CE, Herzyk P, Elmer KR. De novo transcriptome assembly, annotation and comparison of four ecological and evolutionary model salmonid fish species. BMC Genomics. 2018;19.
7. Mansour TA, Rosenthal JJC, Brown CT, Roberson LM. Transcriptome of the Caribbean stony coral *Porites astreoides* from three developmental stages. Gigascience. 2016;5.
8. Frischkorn KR, Harke MJ, Gobler CJ, Dyhrman ST. De novo assembly of *Aureococcus anophagefferens* transcriptomes reveals diverse responses to the low nutrient and low light conditions present during blooms. Front. Microbiol. Frontiers; 2014;5:375.
9. Mansour TA, Scott EY, Finno CJ, Bellone RR, Mienaltowski MJ, Penedo MC, et al. Tissue resolved, gene structure refined equine transcriptome. BMC Genomics. 2017;18:103.
10. Gonzalez VL, Andrade SCS, Bieler R, Collins TM, Dunn CW, Mikkelsen PM, et al. A phylogenetic backbone for Bivalvia: an RNA-seq approach. Proc. R. Soc. B Biol. Sci. 2015;282:20142332–20142332.
11. Müller M, Seifert S, Lübbe T, Leuschner C, Finkeldey R. *De novo* transcriptome assembly and analysis of differential gene expression in response to drought in European beech. PLoS One. 2017;12:e0184167.
12. Heikkinen LK, Kesäniemi JE, Knott KE. *De novo* transcriptome assembly and developmental mode specific gene expression of *Pygospio elegans*. Evol. Dev. 2017;19:205–17.
13. Li F, Wang L, Lan Q, Yang H, Li Y, Liu X, et al. RNA-Seq analysis and gene discovery of Andrias davidianus using Illumina short read sequencing. PLoS One. 2015;10:e0123730.
14. Yu J, Lou Y, Zhao A. Transcriptome analysis of follicles reveals the importance of autophagy and hormones in regulating broodiness of Zhedong white goose. Sci. Rep. 2016;6:36877.
15. Seo M, Kim K, Yoon J, Jeong JY, Lee HJ, Cho S, et al. RNA-seq analysis for detecting quantitative trait-associated genes. Sci. Rep. 2016;6:24375.
16. Pedrotty DM, Morley MP, Cappola TP. Transcriptomic biomarkers of cardiovascular disease. Prog. Cardiovasc. Dis. 2012;55:64–9.
17. Conesa A, Madrigal P, Tarazona S, Gomez-Cabrero D, Cervera A, McPherson A, et al. A survey of best practices for RNA-seq data analysis. Genome Biol. 2016;17:13.
18. Grabherr MG, Haas BJ, Yassour M, Levin JZ, Thompson DA, Amit I, et al. Full-length

transcriptome assembly from RNA-Seq data without a reference genome. Nat. Biotechnol. 2011;29:644–52.
19. Xie Y, Wu G, Tang J, Luo R, Patterson J, Liu S, et al. SOAPdenovo-Trans: De novo transcriptome assembly with short RNA-Seq reads. Bioinformatics. 2014;30:1660–6.
20. Robertson G, Schein J, Chiu R, Corbett R, Field M, Jackman SD, et al. *De novo* assembly and analysis of RNA-seq data. Nat. Methods. 2010;7:909–12.
21. Schulz MH, Zerbino DR, Vingron M, Birney E. Oases: Robust *de novo* RNA-seq assembly across the dynamic range of expression levels. Bioinformatics. 2012;28:1086–92.
22. Bankevich A, Nurk S, Antipov D, Gurevich AA, Dvorkin M, Kulikov AS, et al. SPAdes: A new genome assembly algorithm and its applications to single-cell sequencing. J. Comput. Biol. 2012;19:455–77.
23. Peng Y, Leung HCM, Yiu SM, Lv MJ, Zhu XG, Chin FYL. IDBA-tran: A more robust *de novo* de Bruijn graph assembler for transcriptomes with uneven expression levels. Bioinformatics. 2013. p. i326–34.
24. Kannan S, Hui J, Mazooji K. Shannon : An information-optimal *de novo* RNA-Seq Assembler. biorxiv. 2016;1–14.
25. Chang Z, Wang. Z, Li G. The impacts of read length and transcriptome complexity for *de ovo* assembly: A simulation study. PLoS One. Public Library of Science; 2014;9:e94825.
26. Solomon B, Kingsford C. Fast search of thousands of short-read sequencing experiments. Nat. Biotechnol. 2016;34:300–2.
27. Brown CT, Scott C, Crusoe MR, Sheneman L, Rosenthal J, Howe A. khmer-protocols 0.8.4 documentation. 2013 [cited 2017 Oct 17]; Available from: https://figshare.com/articles/khmer_protocols_0_8_3_documentation/878460
28. Bolger AM, Lohse M, Usadel B. Trimmomatic: A flexible trimmer for Illumina sequence data. Bioinformatics. 2014;30:2114–20.
29. Crusoe MR, Alameldin HF, Awad S, Boucher E, Caldwell A, Cartwright R, et al. The khmer software package: enabling efficient nucleotide sequence analysis. F1000Research [Internet]. 2015 [cited 2017 Oct 17];4. Available from: http://f1000research.com/articles/4-900/v1
30. Zhang Q, Awad S, Brown C. Crossing the streams: a framework for streaming analysis of short DNA sequencing reads. PeeJ Prepr. 2015;0–27.
31. Keeling PJ, Burki F, Wilcox HM, Allam B, Allen EE, Amaral-Zettler LA, et al. The Marine Microbial Eukaryote Transcriptome Sequencing Project (MMETSP): Illuminating the Functional Diversity of Eukaryotic Life in the Oceans through Transcriptome Sequencing. Roberts RG, editor. PLoS Biol. Public Library of Science; 2014;12:e1001889.
32. Caron DA, Alexander H, Allen AE, Archibald JM, Armbrust EV, Bachy C, et al. Probing the evolution, ecology and physiology of marine protists using transcriptomics. Nat. Rev. Microbiol. 2016;15:6–20.
33. Durkin CA, Koester JA, Bender SJ, Armbrust EV. The evolution of silicon transporters in diatoms. J. Phycol. 2016;52:716–31.
34. Groussman RD, Parker MS, Armbrust EV. Diversity and evolutionary history of iron metabolism genes in diatoms. PLoS One. 2015;10:e0129081.
35. Harke MJ, Juhl AR, Haley ST, Alexander H, Dyhrman ST. Conserved transcriptional responses to nutrient stress in bloom-forming algae. Front. Microbiol. Frontiers; 2017;8:1279.
36. Koid AE, Liu Z, Terrado R, Jones AC, Caron DA, Heidelberg KB. Comparative transcriptome analysis of four prymnesiophyte algae. PLoS One. 2014;9:e97801.
37. Alkalaeva E, Mikhailova T. Reassigning stop codons via translation termination: How a few

eukaryotes broke the dogma. BioEssays. 2017;39:1600213.
38. Heaphy SM, Mariotti M, Gladyshev VN, Atkins JF, Baranov P V. Novel ciliate genetic code variants including the reassignment of all three stop codons to sense codons in *Condylostoma magnum*. Mol. Biol. Evol. 2016;33:2885–9.
39. Swart EC, Serra V, Petroni G, Nowacki M. Genetic codes with no dedicated stop codon: context-dependent translation termination. Cell. 2016;166:691–702.
40. Alexander H, Jenkins BD, Rynearson TA, Dyhrman ST. Metatranscriptome analyses indicate resource partitioning between diatoms in the field. Proc. Natl. Acad. Sci. 2015;112:E2182–90.
41. Alexander H, Rouco M, Haley ST, Wilson ST, Karl DM, Dyhrman ST. Functional group-specific traits drive phytoplankton dynamics in the oligotrophic ocean. Proc. Natl. Acad. Sci. 2015;112:E5972–9.
42. Gong W, Browne J, Hall N, Schruth D, Paerl H, Marchetti A. Molecular insights into a dinoflagellate bloom. ISME J. 2017;11:439–52.
43. Lowe EK, Swalla BJ, Brown CT. Evaluating a lightweight transcriptome assembly pipeline on two closely related ascidian species. PeerJ Prepr. 2014;2:e505v1.
44. Leinonen R, Sugawara H, Shumway M. The sequence read archive. Nucleic Acids Res. 2011;39:D19–21.
45. Ewels P, Magnusson M, Lundin S, Käller M. MultiQC: Summarize analysis results for multiple tools and samples in a single report. Bioinformatics. 2016;32:3047–8.
46. MacManes MD. On the optimal trimming of high-throughput mRNA sequence data. Front. Genet. 2014;5:13.
47. Finn RD, Coggill P, Eberhardt RY, Eddy SR, Mistry J, Mitchell AL, et al. The Pfam protein families database: Towards a more sustainable future. Nucleic Acids Res. 2016;44:D279–85.
48. Gardner PP, Daub J, Tate JG, Nawrocki EP, Kolbe DL, Lindgreen S, et al. Rfam: updates to the RNA families database. Nucleic Acids Res. Oxford University Press; 2009;37:D136–40.
49. Zdobnov EM, Tegenfeldt F, Kuznetsov D, Waterhouse RM, Simao FA, Ioannidis P, et al. OrthoDB v9.1: Cataloging evolutionary and functional annotations for animal, fungal, plant, archaeal, bacterial and viral orthologs. Nucleic Acids Res. 2017;45:D744–9.
50. Smith-Unna R, Boursnell C, Patro R, Hibberd JM, Kelly S. TransRate: Reference-free quality assessment of *de novo* transcriptome assemblies. Genome Res. 2016;26:1134–44.
51. Aubry S, Kelly S, Kümpers BMC, Smith-Unna RD, Hibberd JM. Deep evolutionary comparison of gene expression identifies parallel recruitment of trans-factors in two independent origins of C4 photosynthesis. PLoS Genet. 2014;10:e1004365.
52. Simão FA, Waterhouse RM, Ioannidis P, Kriventseva E V., Zdobnov EM. BUSCO: Assessing genome assembly and annotation completeness with single-copy orthologs. Bioinformatics. 2015;31:3210–2.
53. Irber Junior LC, Brown CT. Efficient cardinality estimation for k-mers in large DNA sequencing data sets. biorxiv. 2016;56846.
54. Scott C. dammit: an open and accessible *de novo* transcriptome annotator. in prep. [Internet]. 2016; Available from: www.camillescott.org/dammit
55. Johnson, L; Alexander, H; Brown CT. Marine Microbial Eukaryotic Transcriptome Sequencing Project, re-assemblies [Internet]. 2017. Available from: https://doi.org/10.6084/m9.figshare.3840153.v6
56. Johnson LK, Alexander H, Brown CT. MMETSP re-assemblies. 2018; Available from: https://doi.org/10.5281/zenodo.1212585
57. Li B, Fillmore N, Bai Y, Collins M, Thomson JA, Stewart R, et al. Evaluation of *de novo*

transcriptome assemblies from RNA-Seq data. Genome Biol. 2014;15:553.
58. MacManes MD. The Oyster River Protocol: A multi assembler and kmer approach for *de novo* transcriptome assembly. bioRxiv. 2017;177253.
59. Bradnam KR, Fass JN, Alexandrov A, Baranay P, Bechner M, Birol I, et al. Assemblathon 2: Evaluating de novo methods of genome assembly in three vertebrate species. Gigascience. 2013;2:10.
60. Aranda M, Li Y, Liew YJ, Baumgarten S, Simakov O, Wilson MC, et al. Genomes of coral dinoflagellate symbionts highlight evolutionary adaptations conducive to a symbiotic lifestyle. Sci. Rep. 2016;6:39734.

Quality Metric	Higher in NCGR	Higher in DIB
Transrate score, “cds”	44	583
Transrate score, “nt”	495	143
Mean ORF %	42	596
Percentage of references with CRBB	100	538
Number of contigs	12	626

Table 1. Number of assemblies with higher values in NCGR or DIB assemblies for each quality metric.

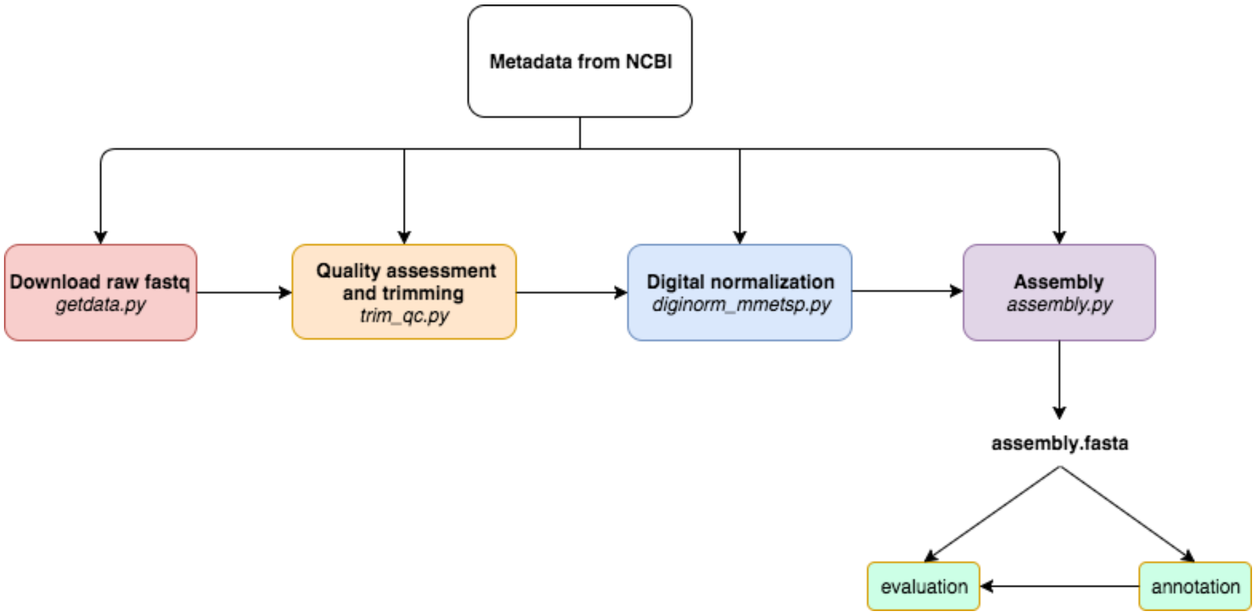

Figure 1. A programmatically automated *de novo* transcriptome assembly pipeline was developed for this study. Metadata in the SraRunInfo.csv file downloaded from NCBI was used as input for each step of the pipeline to indicate which samples were processed. The steps of the pipeline are as follows: download raw fastq data with the fastq-dump script in the SRA Toolkit, perform quality assessment with FastQC and trim residual Illumina adapters and low quality bases (Q<2) with Trimmomatic, do digital normalization with khmer version 2.0, and perform *de novo* transcriptome assembly with Trinity. If a process was terminated, the automated nature of this pipeline allowed for the last process to be run again without starting the pipeline over. In the future, if a new sample is added, the pipeline can be run from beginning to end with just new samples, without having to repeat the processing of all samples in the dataset as one batch. If a new tool becomes available, for example a new assembler, it can be substituted in lieu of the original tool used by this pipeline.

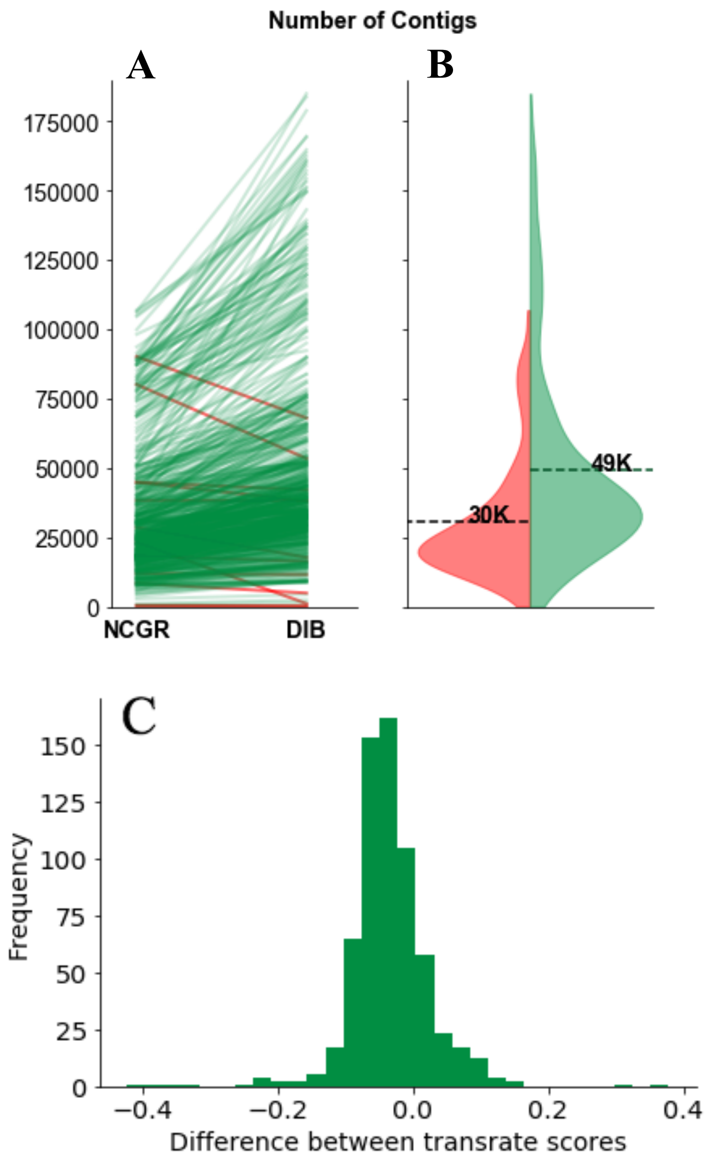

Figure 2. The number of contigs and Transrate quality score for each data set varied between DIB and NCGR assemblies. (A) Slopegraphs show shifts in the number of contigs for each individual sample between the DIB and the NCGR assembly pipelines. Red lines represent values where NCGR was higher than DIB and green lines represent values where DIB was higher than NCGR. (B) Split violin plots show the distribution of the number of contigs in each assembly with the original assemblies from NCGR in red (left) and the DIB re-assemblies and in green (right side of B). (C) The difference in Transrate score between the DIB and NCGR assemblies is shown as a histogram. Negative values on the x-axis indicate that the NCGR assembly had a higher Transrate score and positive values indicate that the DIB assembly had a higher Transrate score.

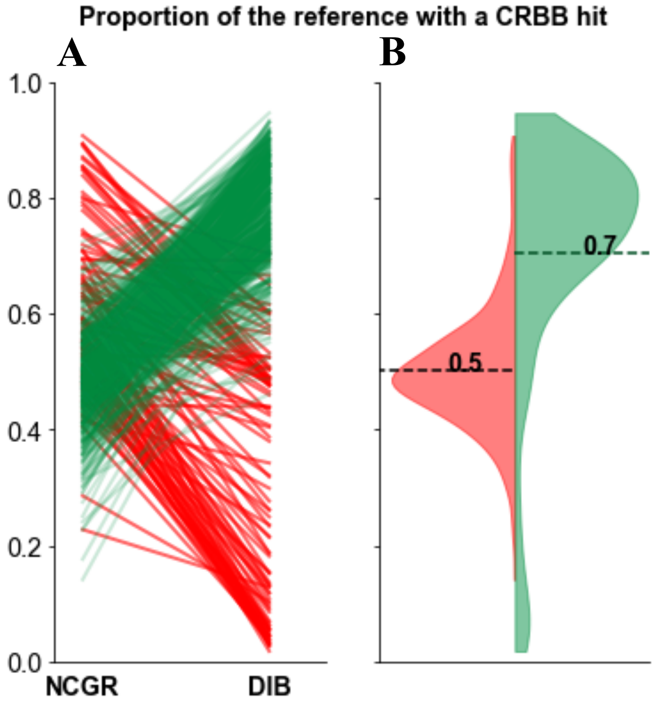

Figure 3. (A) Line plot comparing proportion of CRBB hits between NCGR ‘nt’ assemblies and DIB assemblies between the same samples. (B) Violin plots showing the distribution of the proportion of NCGR transcripts with reciprocal BLAST hits to DIB (red) and the proportion of DIB transcripts with reciprocal BLAST hits to NCGR (green).

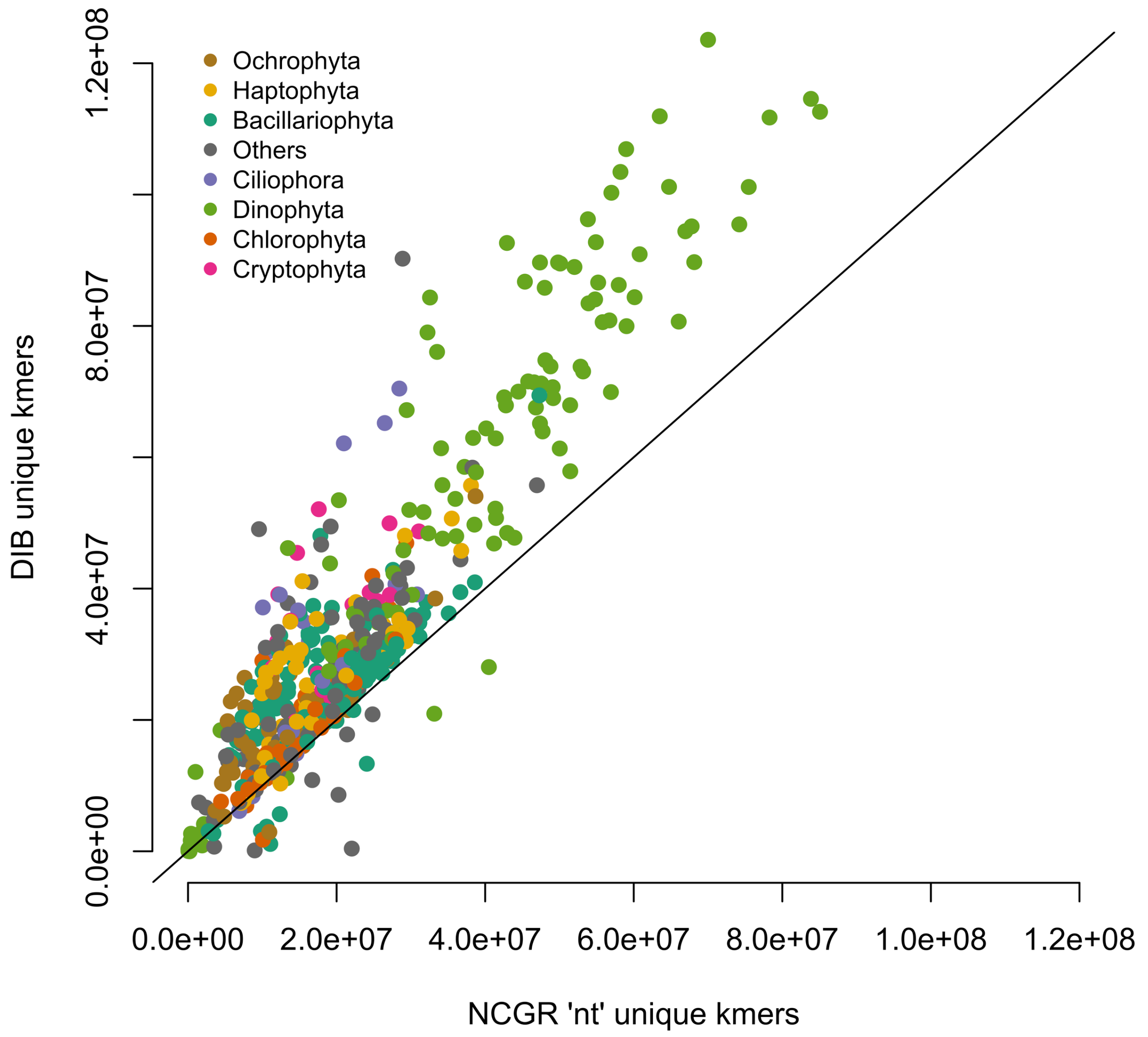

Figure 4. Unique numbers of *k*-mers (*k*=25) in seven most represented phyla, calculated with the HyperLogLog function in the khmer software package. DIB re-assemblies were compared to the NCGR 'nt' assemblies along a 1:1 line. Samples are colored based on their phylum level affiliation. More than 95% of the DIB re-assemblies had more unique *k*-mers than to the NCGR assembly of the same sample.

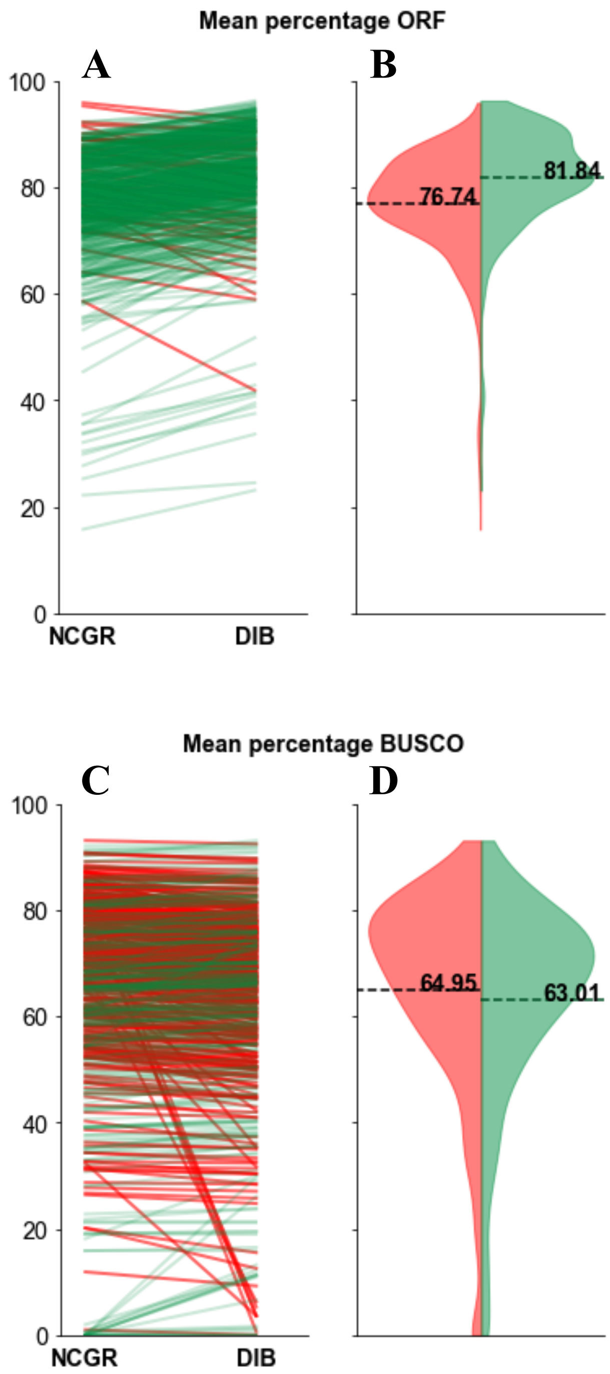

Figure 5. The percentage of contigs with a predicted open reading frame (ORF) (A, B) and the percentage of complete protistan universal single-copy orthologs (BUSCO) recovered in each assembly (C, D). In the green (right side B, D) are the "DIB" re-assemblies and in red (left side of B, D) are the original 'nt' assemblies from NCGR. Line plots (A,C) compare values between the DIB and the NCGR 'nt' assemblies. Red lines represent values where NCGR was higher than DIB and green lines represent values where DIB was higher than NCGR.

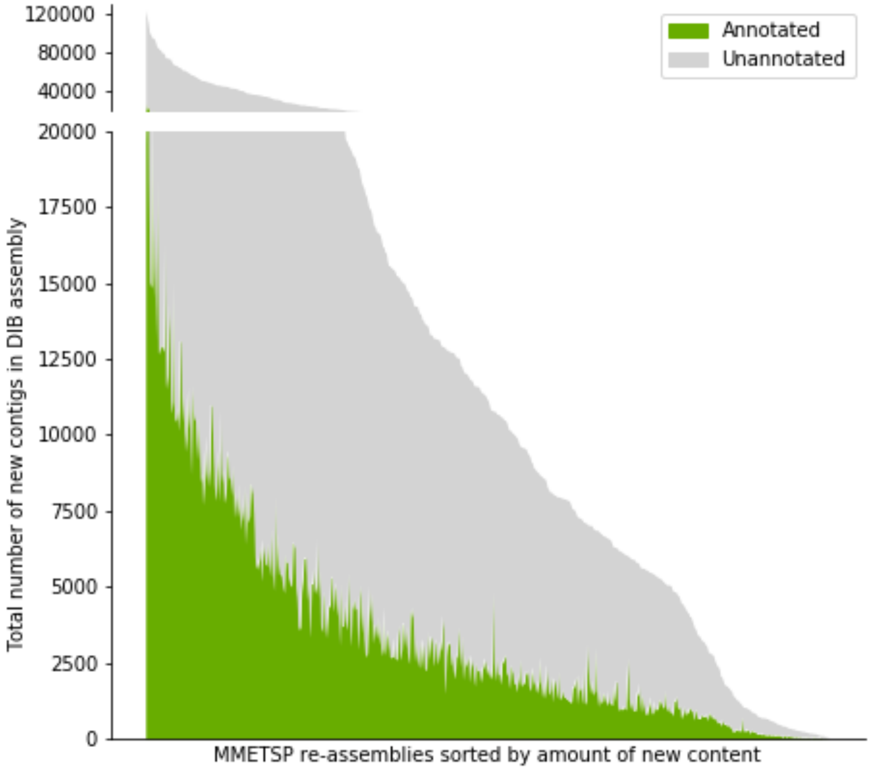

Figure 6. A histogram across MMETSP samples depicting the number of contigs identified as novel in DIB assemblies. These contigs were absent in the NCGR assemblies, based on negative conditional reciprocal best BLAST (CRBB) results. Samples are sorted from highest to lowest number of 'new' contigs. The region in gray indicates the number of unannotated contigs present in the DIB re-assemblies, absent from NCGR 'nt' assemblies. Highlighted in green are contigs that were annotated with dammit [44] to a gene name in the Pfam, Rfam, or OrthoDB databases, representing the number of contigs unique to the DIB re-assemblies with an annotation.

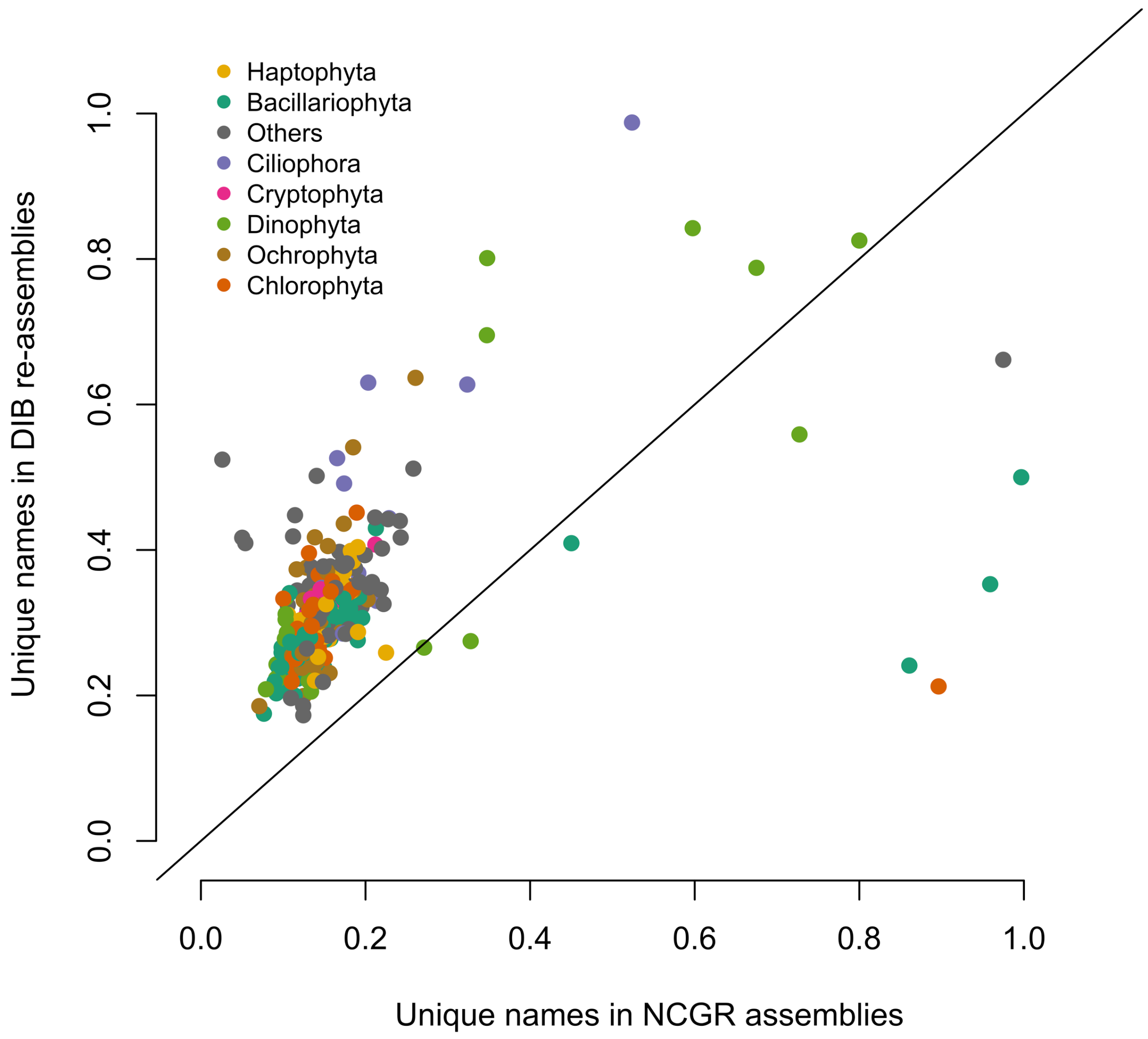

Figure 7. Unique gene names found in a subset (296 samples) of either NCGR 'nt' assemblies or DIB re-assemblies but not found in the other assembly, normalized to the number of annotated contigs in each assembly. The line indicates a 1:1 relationship between the unique gene names in DIB and NCGR. More than 97% of the DIB assemblies had more unique gene names than in NCGR assemblies of the same sample.

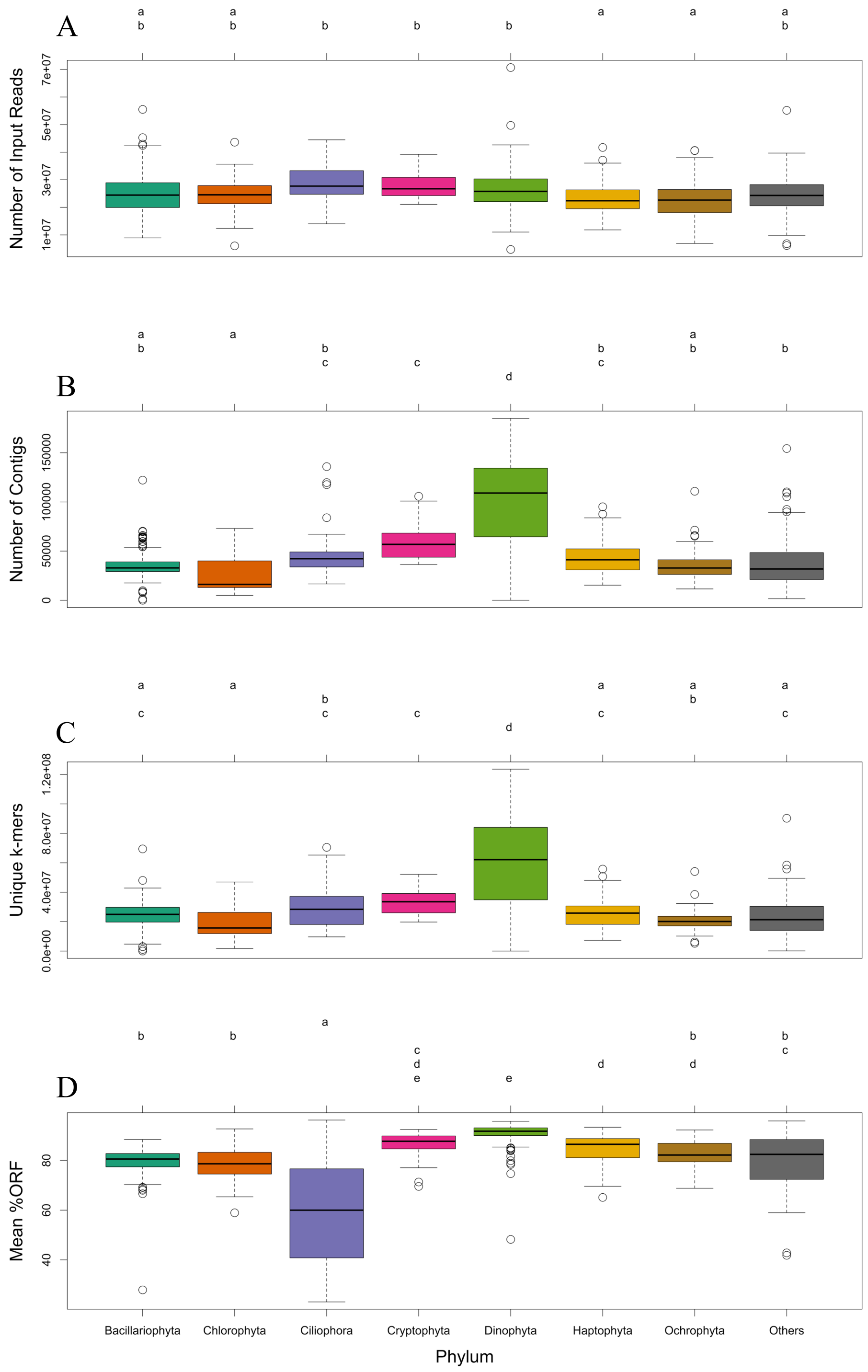

Figure 8. Box-and-whisker plots for the seven most common phyla in the MMETSP dataset, (A) number of input reads, (B) number of contigs in the assembly, (C) unique *k*-mers ($k = 25$) in the assembly, (D) mean percentage open reading frames (ORF). Groups sharing a letter in the top margin were generated from Tukey's HSD post-hoc range test of multiple pairwise comparisons used in conjunction with an ANOVA.

Click here to access/download
Supplementary Material
giga-323576.pdf

Click here to access/download
Supplementary Material
supp-1.txt

Click here to access/download
Supplementary Material
supp-2.txt

Click here to access/download
Supplementary Material
supp-3.html

Click here to access/download
Supplementary Material
supp-4.html

Click here to access/download
Supplementary Material
supp-5.pdf

Click here to access/download
Supplementary Material
supp-6.pdf

Click here to access/download
Supplementary Material
supp-7.pdf
