# Supplementary material for: Re-assembly, quality evaluation, and annotation of 678 microbial eukaryotic reference transcriptomes
Source: Gigascience. 2018 Dec 13;8(4):giy158. doi: 10.1093/gigascience/giy158 (PMC6481552; doi:10.1093/gigascience/giy158)

GigaScience

Re-assembly, quality evaluation, and annotation of 678 microbial eukaryotic reference transcriptomes

--Manuscript Draft--

**Manuscript Number:**	GIGA-D-18-00191R1
**Full Title:**	Re-assembly, quality evaluation, and annotation of 678 microbial eukaryotic reference transcriptomes
**Article Type:**	Research
**Funding Information:**	Gordon and Betty Moore Foundation (GBMF4551) — Dr. C. Titus Brown
**Abstract:**	\textbf{Background} \textit{De novo} transcriptome assemblies are required prior to analyzing RNAseq data from a species without an existing reference genome or transcriptome. Despite the prevalence of transcriptomic studies, the effects of using different workflows, or "pipelines", on the resulting assemblies are poorly understood. Here, a pipeline was programmatically automated and used to assemble and annotate raw transcriptomic short read data collected by the Marine Microbial Eukaryotic Transcriptome Sequencing Project (MMETSP). The resulting transcriptome assemblies were evaluated and compared against assemblies that were previously generated with a different pipeline developed by the National Center for Genome Research (NCGR). \textbf{Results} New transcriptome assemblies contained the majority of previous contigs as well as new content. On average, 7.8\% of the annotated contigs in the new assemblies were novel gene names not found in the previous assemblies. Taxonomic trends were observed in the assembly metrics, with assemblies from the Dinoflagellata and Ciliophora phyla showing a higher percentage of open reading frames and number of contigs than transcriptomes from other phyla. \textbf{Conclusions} Given current bioinformatics approaches, there is no single 'best' reference transcriptome for a particular set of raw data. As the optimum transcriptome is a moving target, improving (or not) with new tools and approaches, automated and programmable pipelines are invaluable for managing the computationally-intensive tasks required for re-processing large sets of samples with revised pipelines and ensuring a common evaluation workflow is applied to all samples. Thus, re-assembling existing data with new tools using automated and programmable pipelines may yield more accurate identification of taxon-specific trends across samples in addition to novel and useful products for the community.
**Corresponding Author:**	C. Titus Brown<br>UC Davis<br>Davis, CA UNITED STATES
**Corresponding Author Secondary Information:**	
**Corresponding Author's Institution:**	UC Davis
**Corresponding Author's Secondary Institution:**	
**First Author:**	Lisa Kristine Johnson
**First Author Secondary Information:**	
**Order of Authors:**	Lisa Kristine Johnson
	Harriet Alexander
	C. Titus Brown
**Order of Authors Secondary Information:**	
**Response to Reviewers:**	Reviewer #1: The manuscript submitted by Lisa Johnson and colleagues is a well written and comprehensive work aimed at reanalyzing a fairly enormous dataset. I feel that the work is important for the following two main reasons:<br>1. Assembly methods have improved substantially since the original datasets were

analyzed, and as the authors point out - these new analyses recover new transcripts that might be useful to the original researchers and to the broader community.
2. Applying standardized and reproducible methods - at scale - is challenging, and the authors provide an example for how this could be done. I can imagine others using these ideas (or the actual code) to assemble other datasets in a similar fashion.
In terms of the manuscript itself, it is sound, with just a few areas where improvements will make for a more readable paper. Interspersed with this, I have a few more pedantic suggestions that the author should feel free to ignore if deemed unhelpful.

We thank the reviewer for their helpful comments and insights into our work. Below we note alterations and some more detailed clarifications to address the comments.

Line 91: replace 'higher' with 'more favorable' or even 'better'

Changed to: "Here, we show that our re-assemblies had better evaluation metrics and contained most of the NCGR contigs as well as adding new content."

L102: The link to the code does not seem to be active. I would have loved to review it.

The link is here: https://doi.org/10.5281/zenodo.594854 and active in the current version of the GigaScience formatted manuscript.

L111: You are using 50bp reads. Do you think your conclusions would have been any different had longer reads (100-150bp) been available? More novice readers might wonder if these methods are just as applicable to them with longer reads as they are to you. I'm sure the answer is yes - your new assemblies might have been even better had you had longer reads.

Thank you for bringing this point to our attention. This is an important point to mention in the discussion. We added to the end of the second section of the discussion as an extension of subheading: "Reassembly with new tools can yield new results." (L327):

```
We predict that assembly metrics could have been further improved with longer read lengths of the original data since MMETSP data had only 50 bp read lengths, although this would have presented Keeling et al. [31] a more expensive data collection endeavor. A study by Chang et al. [[25](https://www.ncbi.nlm.nih.gov/pmc/articles/PMC3988101/)] reported a consistent increase in the percentage of full-length transcript reconstruction and a decrease in the false positive rate moving from 50 to 100 bp read lengths with the Trinity assembler. However, regardless of length, the conclusions we draw here would likely remain the same that assembling data with new tools can yield new results.
```

L180-182: If I didn't know khmer already, I might struggle with the HyperLogLog estimator. Maybe just a sentence or 3 more might be useful to explain what this is and why it is used.

L182 added: "We used the HLL function to digest each assembly and count the number of distinct fixed-length substrings of DNA (k-mers)."

L245ish: I keep wondering about your BUSCO scores, and the fact tat they are lower on average in your new assemblies compared to the older ones. Why is this? How do you reconcile this with the more general statement you are trying to make about 'more genes being recovered' in the new assembly. I see that BUSCO is just one of the available metrics to assess this, but it's a little strange I think, given that I'm convinced that these assemblies are actually better.

On average, BUSCO scores were lower in DIB vs. NCGR assemblies. However, the degree of the differences was not as dramatic (NCGR mean 64.8 vs. DIB 62.9) compared to differences between the number of contigs (NCGR length mean 30K vs. DIB 49K) and the CRBB differences (NCGR mean proportion 0.5 vs. DIB 0.7).

After re-checking the BUSCO v3 scores against the eukaryota and protista databases, we changed the mean, sd, and k-s test numbers listed in the text. The original numbers were missing a few assemblies and were from BUSCO version 2. The % complete BUSCO are still significantly higher in NCGR vs. DIB.

We edited this sentence and emphasized the less dramatic differences in % ORF and BUSCO scores relative to contig number and CRBB differences:

""
Therefore, although the number of contigs and amount of CRBB content were dramatically increased in the DIB re-assemblies compared to the NCGR assemblies, the differences in ORF content and BUSCO matches compared to the eukaryotic (Figure 5 C,D) and protistan (Supplemental Figure 3) databases - while they were significantly different - were less dramatic. This suggests that content was not lost by gaining extra contigs. The extra content contained similar proportions of ORFs and BUSCO annotations. Therefore, the re-assemblies may contribute more biologically meaningful information.

""

Looking at the eight samples where NCGR had >30% higher complete BUSCO evaluation score (MMETSP0121, MMETSP0932, MMETSP0045, MMETSP0169, MMETSP0232, MMETSP0439, MMETSP0329, MMETSP0717), we see different reasons for missing Complete BUSCOs.

```
    SampleName  Complete_BUSCO_perc_NCGR  Complete_BUSCO_perc_DIB
18   MMETSP0121                65.016502                31.683168
134  MMETSP0932                85.148515                 3.630363
232  MMETSP0045                73.927393                35.313531
282  MMETSP0169                68.646865                 6.270627
451  MMETSP0232                82.508251                 0.660066
475  MMETSP0439                80.858086                 3.630363
654  MMETSP0329                80.198020                 5.280528
661  MMETSP0717                61.716172                17.821782
```
(BUSCO output is not straight-forward to parse.)

https://github.com/ljcohen/MMETSP/blob/master/assembly_evaluation_data/busco_eval/busco_eval.ipynb

For reasons that we don't understand, in some cases a particular orthogroup in the BUSCO db does not produce output in `hmmer_output/`. Sometimes it does. For example, the Trininty-based pipeline only produced 342 contigs for sample MMETSP0232 while the NCGR 'nt' assembly had 4234 and 'cds' had 2736. BUSCO did not recognize any of the DIB contigs but it did recognize the NCGR contigs. Whereas for other samples, e.g. MMETSP0169, the BUSCO software recognized several contigs but did not score them high enough and called the BUSCO group "missing", even though there were lengths of contigs identified as being somewhat related.

This is a case where it does suggest several contigs are matching in DIB hmmer output, even though it is listed as "Missing":

MMETSP0169:

```
EOG0937060I
DIB: ['EOG0937060I', 'Missing']
NCGR: ['EOG0937060I', 'Duplicated', 'CAMNT_0039020233', '576.3', '630']
NCGR: ['EOG0937060I', 'Duplicated', 'CAMNT_0039023937', '555.9', '644']
NCGR: ['EOG0937060I', 'Duplicated', 'CAMNT_0039030405', '552.2', '636']
DIB contig: MMETSP0169-figshare3840153v7-TRINITY_DN13758_c3_g2_i1_3
```

Powered by Editorial Manager® and ProduXion Manager® from Aries Systems Corporation

	DIB contig length: 974<br>DIB contig: MMETSP0169-figshare3840153v7-TRINITY_DN3716_c0_g1_i1_1<br>DIB contig length: 154<br>DIB contig: MMETSP0169-figshare3840153v7-TRINITY_DN13467_c1_g1_i1_6<br>DIB contig length: 494<br>NCGR contig: CAMNT_0039023937_4<br>NCGR contig length: 781<br>NCGR contig: CAMNT_0039030405_5<br>NCGR contig length: 921<br>NCGR contig: CAMNT_0039020233_3<br>NCGR contig length: 842<br>```<br>The gene is:<br>```<br>DNA/RNA helicase, ATP-dependent, DEAH-box type, conserved site<br>Similarity:Contains helicase ATP-binding domain<br>```<br>When we look for this gene in the annotation file, there are no annotation results for this contig:<br>```<br>[ljcohen@dev-intel14 MMETSP0169.trinity_out_2.2.0.Trinity.fasta.dammit]$ grep "TRINITY_DN13758_c3_g2_i1" MMETSP0169.trinity_out_2.2.0.Trinity.fasta.dammit.namemap.csv<br>```<br>But there are for other contigs identified by the hmm_output file:<br>```<br>[ljcohen@dev-intel14 MMETSP0169.trinity_out_2.2.0.Trinity.fasta.dammit]$ grep "TRINITY_DN3716_c0_g1_i1" MMETSP0169.trinity_out_2.2.0.Trinity.fasta.dammit.namemap.csv<br>"TRINITY_DN3716_c0_g1_i1 len=208 path=[186:0-207] [-1, 186, -2]",Transcript_32368<br><br>[ljcohen@dev-intel14 MMETSP0169.trinity_out_2.2.0.Trinity.fasta.dammit]$ grep "Transcript_32368" MMETSP0169.trinity_out_2.2.0.Trinity.fasta.dammit.gff3<br>Transcript_32368LASTtranslated_nucleotide_match151671.300000e-06-<br>.ID=homology:18169;Name=ENSXMAP00000012674;Target=ENSXMAP00000012674 244 295 +;database=OrthoDB<br>```<br>Looking at the gene identified by the annotations:<br>```<br>[ljcohen@dev-intel14 reference]$ grep "ENSXMAP00000012674" ODB8_EukOGs_genes_ALL_levels.txt<br>7898:ActinopterygiiEOG808R8MENSXMAP000000126748083:003153Xiphophorus maculatus<br>33208:MetazoaEOG82JQ5DENSXMAP000000126748083:003153Xiphophorus maculatus<br>1261581:VertebrataEOG808P6WENSXMAP000000126748083:003153Xiphophorus maculatus<br><br>[ljcohen@dev-intel14 reference]$ grep "EOG808R8M" odb_v8_v9_1.tab<br>7898EOG808R8MEOG090C08EI.39456585<br>```<br>This does not match the original EOG0937060I and is a different gene:<br>```<br>Abl-interactor, homeo-domain homologous domain<br>ABI family, member 3a<br>```<br><br><br>But, the top ncbi blastn results with the contig sequence suggested small (only several hundred bp) region matches with the EOG0937060I gene sequence:<br>```<br>PREDICTED: Heterocephalus glaber DEAH-box helicase 37 (Dhx37), transcript variant X2, mRNA66.266.21%6e-0686%XM_021257656.1<br>Select seq XM_004843976.2PREDICTED: Heterocephalus glaber DEAH-box helicase 37 (Dhx37), transcript variant X1, mRNA66.266.21%6e-0686%XM_004843976.2

Powered by Editorial Manager® and ProduXion Manager® from Aries Systems Corporation

Select seq XM_010604294.2PREDICTED: Fukomys damarensis DEAH-box helicase 37 (Dhx37), transcript variant X6, mRNA66.266.21%6e-0686%XM_010604294.2
Select seq XM_010604293.2PREDICTED: Fukomys damarensis DEAH-box helicase 37 (Dhx37), transcript variant X5, mRNA66.266.21%6e-0686%XM_010604293.2
Select seq XM_010604292.2PREDICTED: Fukomys damarensis DEAH-box helicase 37 (Dhx37), transcript variant X4, mRNA66.266.21%6e-0686%XM_010604292.2
Select seq XM_010604291.2PREDICTED: Fukomys damarensis DEAH-box helicase 37 (Dhx37), transcript variant X3, mRNA66.266.21%6e-0686%XM_010604291.2
Select seq XR_776390.2PREDICTED: Fukomys damarensis DEAH-box helicase 37 (Dhx37), transcript variant X2, misc_RNA66.266.21%6e-0686%XR_776390.2
Select seq XM_019204571.1PREDICTED: Fukomys damarensis DEAH-box helicase 37 (Dhx37), transcript variant X1, mRNA66.266.21%6e-0686%XM_019204571.1
Select seq XM_005403001.2PREDICTED: Chinchilla lanigera DEAH (Asp-Glu-Ala-His) box polypeptide 37 (Dhx37), mRNA
```

Even though this contig was assembled, it did not successfully annotate. We don't know whether there are errors associated with this assembled contig, or if the contig is a new sequence unique to this MMETSP0169 organism (*Corethron pennatum*, Phylum: Bacillariophyta). Since the BUSCO database and corresponding orthogroups were contstructed from multiple sequence alignments with inidividuals already in the databases, it is possible that different organisms have evolved slightly different sequences that may fall outside the hmm scoring cutoffs for matching with the BUSCO orthogroup. Since the corresponding NCGR assembly had a "Duplicated" result from this particular BUSCO, it is possible that there is a particular oddity within this ortholog.

This is an example where there is not a file in the DIB `run_MMETSP0169/hmmer_output` correspondint to this orthogroup EOG.

```
EOG0937017X
DIB: ['EOG0937017X', 'Missing']
NCGR: ['EOG0937017X', 'Complete', 'CAMNT_0039023621', '802.2', '1208']
NCGR contig: CAMNT_0039023621_3
NCGR contig length: 1352
NCGR contig: CAMNT_0039027673_4
NCGR contig length: 1287
NCGR contig: CAMNT_0039044891_4
NCGR contig length: 1358
```

Sifting through BUSCO output, there are more examples that could be picked over to explore this issue with these eight MMETSP samples with 30% difference between NCGR and DIB:

https://github.com/ljcohen/MMETSP/blob/master/assembly_evaluation_data/busco_eval/busco_eval.ipynb

For now, we conclude that our assemblies are differently fragmented in some regions relative to the NCGR assemblies. We have assembled additional sequences that were not assembled by NCGR. Some NCGR assemblies had different and more complete content than the DIB assemblies. As far as we can tell, there does not appear to be a pattern in the samples that fared well with this pipeline vs. NCGR. This could be a future avenue to explore.

Could you (did you) do a CRHB against Swiss-prot? I imagine that for each assembly pair (old assembly vs new assembly), you'd see more hits to unique Swiss-Prot genes in the newer assembly.

The dammit pipeline we used did perform a CRBB with Pfam, Rfam, Orthodb. A CRBB directly with Swiss-prot would probably not give additional information since Pfam is a collection of protein family alignments constructed using HMM of multiple Swiss-prot sequence alignments. Directly querying Swiss-prot might be an interesting avenue to

Powered by Editorial Manager® and ProduXion Manager® from Aries Systems Corporation
```

pursue in the future to confirm annotations with Pfam, R-fam and Orthodb with this high quality annotated and non-redundant protein sequence database. But at this point it might be redundant and more noisy given the biodiversity and evolutionary divergence among the species found in the MMETSP collection.

L254: "less significantly different" Do you mean "significantly less"?

The difference in BUSCO was significant, but to a lesser degree than other distribution comparisons (p=0.002 rather than p < 0.001). I edited the wording here, see comment above.

L306: I'm also confused about the TransRate scores. As best as I can tell the "NT" assemblies were the raw assemblies, while the "CDS" assemblies were further filtered. If my understanding is correct, then the opening statement for this paragraph (DIB assemblies were more inclusive) is incorrect, given that transrate metrics were higher for the NCGR nt assemblies that they were for the DIB assemblies. I'm also worried about the statements about DIB assemblies being better, while transrate scores were on whole, worse. Should reconcile this.

Thank you for pointing out the imprecise language in our explanation. Yes, the "nt" assemblies were raw assemblies, whereas the "cds" assemblies that the NCGR published were filtered for only coding sequences, as far as we can tell. (Methods described by Keeling et al. 2014 were not transparent enough to correspond to the file nomenclature located on ftp://ftp.imicrobe.us/projects/104/.) Transrate scores may not be the best metric for comparison. The transrate score is a measurement of how well the original reads support the the final assembly. We have more unique k-mers, but we have worse transrate scores.

We edited "suggesting that both pipelines yielded equally valid contigs," to "suggesting that both pipelines yielded similarly valid contigs," so that the significant BUSCO and ORF differences between assemblies can be acknowledged.

To the discussion, we added:
""
Moreover, even though the number of contigs and the CRBB results between the DIB and NCGR assemblies were dramatically different, both the fraction of contigs with ORFs and the mean percentage of BUSCO matches were similar between the two assemblies, suggesting that both pipelines yielded similarly valid contigs, even though the NCGR assemblies were less sensitive.
""

L315: I'm not sure that you are "directly" evaluating the de Bruijn structure.

This is correct. We did not directly evaluate the de Bruijn graph structure. The sentence in the discussion mentions:

Metrics directly evaluating the underlying de Bruijn graph data structure used to produce the assembled contigs may be better evaluators of assembly quality.

We clarified this by adding:

In future studies, metrics directly evaluating the underlying de Bruijn graph data structure used to produce the assembled contigs may be better evaluators of assembly quality in the future.

L320: I'm not sure you show "Biologically meaningful". You show that you have recovered new stuff that is likely real (not an artifact of asembly), but not sure you can claim it's meaningful.

This is true. We changed the word "meaningful" to "relevant".

L364: In your discussion of kmer content (and other metrics) the idea that some of

these datasets might in fact be meta-transcriptomes should be discussed. Lots of marine microeukaryotes associate with bacteria, viruses, etc, and unless extreme care was takes with the target species, to grow in sterile conditions, some of patterns of kmer distrib. might be because the datasets contain more than 1 species.

We added a paragraph:

""
RNA sequences generated from the MMETSP experiments are likely to contain genetic information from more than the target species, as many were not or could not be cultured axenically. Thus both the NCGR assemblies and DIB re-assemblies, including the additional biologically relevant information, might be considered meta-transcriptomes. Sequencing data and unique k-mer content likely include bacteria, viruses, or other protists that occurred within the sequenced sample. We did not make an attempt to de-contaminate the assemblies.
""

Table 1. Can you include the BUSCO results here?

Yes, these were added.

Fig3 needs a y-axis label

Done.

Fig 5c and a few other places. There are a few DIB assemblies that are WAY worse than the original assembly. Why? This could benefit from some explanation.

Added to Discussion:

""
For some samples, the DIB re-assemblies had lower metrics than the NCGR assemblies. Complete BUSCO scores were lower than over half of DIB vs. NCGR. This could be an effect of the BUSCO metric, given that these samples did not perform poorly with other metrics, such as \% ORF and number of contigs compared to the NCGR. For other samples, MMETSP0252 (*Prorocentrum lima*) in particular, assemblies required several tries and only four contigs were assembled from 30 million reads of data. The fastqc reports were unremarkable compared to the other samples. In such a large dataset with a diversity of species with no prior sequencing data to compare make it challenging to speculate why each anomaly occurred. However, further investigation into the reasons for failures and peculiarities in the evaluation metrics may lead to interesting discoveries about how we should be effectively assembling and evaluating nucleic acid sequencing data from a diversity of species.
""

In the end, what I took away from this paper is that the new assemblies had different transcripts, and this is great and potentially helpful for researchers. Saying that, on a whole, both BUSCO and TransRate scores trended toward lower, which is maybe surprising, especially because the original assemblies were assembled (best as I can tell) using a general genome assembler (ABySS/MIRA) rather than software specialized for transcriptomes.

This is a good summary of our findings, thank you. We added a sentence to the Introduction mentioning the ABySS assembler and cited Keeling et al. 2014 for methods (LINE 78 in the formatted manuscript).

Reviewer #2: The manuscript by Johnson et al. describe the re-analysis of the Marine Microbial Eukaryotic Transcriptome Sequencing Project (MMETSP). The authors have generate a new computational pipeline for the de novo assembly (using Trinity de novo) of the RNA-Seq reads of several hundred transcriptomes as well as downstream a set of scripts to compare the outcome with the results of the original publication (which used Trans-ABySS for the assembly).
The current manuscript is a great example that shows the value of revisiting old data sets with new computational tools. The authors put strong focus on reproducibility of

Powered by Editorial Manager® and ProduXion Manager® from Aries Systems Corporation

their analysis. The effort for this should not be underestimated and the work can serve as a blueprint for similar data re-analysis projects.
I see no major issue in this work but still would like to have a few smaller ones addressed:

We thank the reviewer for their compliments on our work and appreciate the feedback they have provided. Below please find our responses to the the detailed comments:

* The manuscript is currently rather descriptive and has only a few explanations why there are certain differences in the presented assembly approaches. E.g. what are the reasons for the observation displayed in Figure 4 that there so many more unique k-mers in the DIB than in the NCGR set? Maybe not all results can be explained mechanistically but least at some potential reasons could be discussed.

Thank you for pointing this out-- we have tried to expand our explanations where we felt confident in our reasoning. For example, we have added the following explanation for why greater kmer content is conserved by the DIB assembly:
Added to L316

""
The relative increase in number of unique k-mers from the NCGR assemblies to the DIB re-assemblies could be an effect of having more contigs. Within the data, the Trinity assembler found evidence for building alternative isoforms. Whereas the ABySS assembler and transcriptome pipeline that NCGR used may not have preserved that variation, in an attempt to narrow down the contigs to a consensus transcript sequence.
""

* The authors write: "We used a different pipeline than the original one used to create the NCGR assemblies, in part because new software was available [8] and in part because of new trimming guidelines [27]". Is [8] really the correct reference here? If so this has to be further explained.

Thank you for catching this. This was a typo. Fixed to reference [18].

* I think figures 2, 3 and 5 are not red green blind safe.

You are correct-- thank you for bringing this to our attention. We have changed the figures to blue and yellow.

* In the script collection uploaded to Zenodo I personally would have removed the "pycache" folder and the containing Python byte code files (*pyc). Or do they have any purpose / contain useful information?

Thank you, we will make sure to remove this before publication.

* The supplementary notebooks could additionally be uploaded as ipyn files.

Thank you for the suggestion. We have uploaded the notebooks both as ipynb files as static html pages to a figshare: https://doi.org/10.6084/m9.figshare.7091003

* The authors have a configuration file for user specif paths but this is not strictly used. In "dibMMETSPconfiguration.py" another "basedir" variable is set and in trimqc.py even the full path for Trimmomatic is set ("/mnt/home/ljcohen/bin/Trimmomatic-0.33/trimmomatic-0.33.jar"). This make the reuse of the framework harder.

Thank you for catching this, we have removed absolute paths and added the edited the files to a new version of the repository on zenodo. While we agree that it does make sense for future reuse, we felt it was important to preserve the code in its original state used to generate the assembly files discussed in the paper.

* While I understand that it is sometime needed due to dependencies on old libraries I would like to discourage the use of Python 2.7 (aka "legacy Python") in currently

Powered by Editorial Manager® and ProduXion Manager® from Aries Systems Corporation

research projects and would strongly recommend to use a current Python version (3 and higher) instead.

We completely agree. At the time the scripts were written they were in Python 2.7. The assemblies discussed in the paper were generated with this code. Future versions of this pipeline are written in Python 3.

Reviewer #3:
In the manuscript, Johnson et al have reassembled RNA-seq data from 678 samples generated from MMETS Project using a pipeline, which follows the Eal Pond mRNA seq protocol. The pipeline (DIG) starts by quality trimming the data followed by digital normalization and assembly using the Trinity assembler. The authors have compared their re-assemblies against assemblies generated from the method suggested by the National Center for Genome Resource (NCGR). For comparison, they have used difference evaluation metrics like Conditional Reverse Best BLAST (CRBB), BUSCO scores, annotation using the Dammit pipeline and ORF content in the assembly. They argued that their pipeline is able to provide additional biologically meaningful content as compared to the NCGR pipeline. While the work overall is quite interesting and the large set of assemblies appear useful, I feel that there are some improvements and clarifications necessary:
Major comments:
1) The core reason behind the observation that DIG pipeline being better than the NCGR pipeline is not clear. It might be due to the core algorithm behind the assembler used by the pipelines (DIG using Trinity and NCGR uses AbySS). But this should be explained in more detail why their pipeline performs better. For example, is the performance increase linked to sequencing coverage of the read data sets? Or transcriptome complexity of the sample? Or is it the fact that the NCGR pipeline seems to use a custom build pipeline that uses multi-kmer ABySS but not the de novo transcriptome assembler trans-ABySS, which may be more suited?

Thank you for bringing this to our attention. We agree that we did not discuss this point thoroughly enough in the text. The main differences between the NCGR and DIB pipelines were the assemblers used (we used Trinity) and trimming parameters (MacManes 2014). Additionally, in postprocessing, we did not filter out assembly contigs for ORF content whereas the NCGR did in their 'cds' versions.**

We have added the following text to the discussion that explains that we believe the reason for improved metrics with the DIB pipeline relative to the NCGR could be because of increased contigs from the Trinity assembly and trimming parameters used.

""
The increase in number of unique k-mers from the NCGR assemblies to the DIB re-assemblies could due to the higher number of contigs generated by Trinity. Within the data, the Trinity assembler found evidence for building alternative isoforms. Whereas the ABySS assembler and transcriptome pipeline that NCGR used \citep{Keeling_2014} appears to not have preserved that variation, in an attempt to narrow down the contigs to a consensus transcript sequence.
""

2) The other major difference between the pipelines is the additional step of digital normalization which DIG uses. Normalization generally removes kmer information, which affect the overall assembly. It is not clear why normalization in case of DIG should improve the assemblies. Normally the expectation would not that the digital normalization leads to an improvement. So I assume the authors do it simply to reduce the computational costs of the many assemblies, which is plausible but should be stated.

Yes, our DIB pipeline used digital normalization with khmer, which is much like Trinity normalization, to remove redundant k-mers for the purpose of reducing computational resources required. As Brown et al (2012) showed, digital normalization does not affect the content of the resulting transcriptome. We clarified on line 144: "To decrease the memory requirements for each assembly, digital normalization was applied prior to

assembly [46]."

Also, Trinity by default performs in-silico normalization. So, the additional normalization step is redundant. Is the option for normalization switched off in the assembler. If yes, the authors should comment on why they are using Diginorm instead of using Trinity's built-in normalization, is there any indication that this works better for the assemblies they have done?

We used an older version of Trinity (2.2.0) which did not have this turned on by default. We added: "This version of Trinity (2.2.0) did not include the “in silico normalization” option as a default parameter.

The digital normalization used here is the same algorithm as the Trinity in silico normalization, but it requires considerably less memory and is faster (Brown et al. 2012).

3) It is not clear which version of NCGR assemblies ("nt" or "cds") the authors used for calculating the mean ORF% in Table 1. If they have used the "nt" version, then the number can be misleading. The "cds" version of the NCGR assemblies contains contigs that have been predicted to show coding potential and hence might have a higher mean ORF content (as this is computed as percentages). I suggest the authors compare the mean ORF% content of the two NCGR version against the assemblies generated using DIG for full transparency and then discuss the differences regarding these two NCGR version and their assemblies.

Thank you for pointing this out. We have clarifed clarified the mean % ORF metric in Table 1, which originally only included the "nt" version of the NCGR assembly, and added the assembly numbers corresponding to the "cds" version of the NCGR assembly. The DIB re-assemblies were more comparable to the "nt" versions of NCGR since we did not filter contigs based on ORF content, which the NCGR did in their "cds" version. When filtration steps were performed, potentially useful content was lost. Explanations clarifying the comparability of the DIB to the "nt" and the "cds" versions of the NCGR assemblies were added to the results and discsussion.

4) I think the line plots used in the paper can be improved, because it is hard to quantify the amount of overlapping lines. For example I think that Figure 2A, 3A,5A,5C are probably more easy to interpret when made as a scatterplot, e.g. Fig2A where the number of contigs is compared between NCGR and DIB assemblies.

We appreciate this feedback; however, we feel that in this context, the line plots are visually drawing the relationship between the same sample for each NCGR and DIB assembly. Scatter plots would not show that relationship. We attempted to clarify this point by adding this sentence (Figure 2, line 151 in the formatted manuscript)

""
Slopegraphs show shifts in the number of contigs for each individual sample between the DIB and the NCGR assembly pipelines. Negative slope (brown) lines represent values where NCGR was higher than DIB and positive slope (blue) lines represent values where DIB was higher than NCGR.
""

5) I would not say that the distribution in Figure 2c looks like a Normal distribution as the right tail is much heavier than the left one. If you want to make that statement, use a test of normality, however I feel this is not important for the paper.

Changed to:

""
The frequency of the differences between Transrate scores in the NCGR ‘nt’ assemblies and the DIB re-assemblies is centered around zero (Figure 2C).
""

Powered by Editorial Manager® and ProduXion Manager® from Aries Systems Corporation

	Minor comments:<br>-Typo in reference 25 .. de ovo assembly ..<br>Thank you for catching this typo. We have fixed it in the references.<br><br>-line 336: I was not able to understand what the (see op-ed Alexander et al. 2018) refers to, as there is no such reference in the bibliography and no footnote<br>We were not able to post to this article to a preprint server as it was not original research. Here is a link to the current github repo for the text of the citation: https://github.com/dib-lab/2018-paper-reanalysis-op-ed<br><br>The final version of this paper will include a proper citation.
**Additional Information:**	
**Question**	**Response**
Are you submitting this manuscript to a special series or article collection?	No
**Experimental design and statistics**<br><br>Full details of the experimental design and statistical methods used should be given in the Methods section, as detailed in our Minimum Standards Reporting Checklist. Information essential to interpreting the data presented should be made available in the figure legends.<br><br>Have you included all the information requested in your manuscript?	Yes
**Resources**<br><br>A description of all resources used, including antibodies, cell lines, animals and software tools, with enough information to allow them to be uniquely identified, should be included in the Methods section. Authors are strongly encouraged to cite Research Resource Identifiers (RRIDs) for antibodies, model organisms and tools, where possible.<br><br>Have you included the information requested as detailed in our Minimum Standards Reporting Checklist?	Yes
**Availability of data and materials**	Yes

All datasets and code on which the conclusions of the paper rely must be either included in your submission or deposited in publicly available repositories (where available and ethically appropriate), referencing such data using a unique identifier in the references and in the “Availability of Data and Materials” section of your manuscript.<br><br>Have you have met the above requirement as detailed in our Minimum Standards Reporting Checklist?	

Manuscript (PDF rendered)
Click here to view linked References
Click here to access/download;Manuscript;assembly-quality-evaluation (3).pdf

OXFORD (GIGA)n SCIENCE

GigaScience, 2018, 1–12

doi: xx.xxxx/xxxx
Manuscript in Preparation
Technical Note

TECHNICAL NOTE

Re-assembly, quality evaluation, and annotation of 678 microbial eukaryotic reference transcriptomes

Lisa K. Johnson[1,2], Harriet Alexander[1] and C. Titus Brown[1,2,3,*]

[1]Department of Population Health & Reproduction, School of Veterinary Medicine, University of California Davis and [2]Molecular, Cellular, and Integrative Physiology Graduate Group, University of California Davis and [3]Genome Center, University of California Davis

[*]ctbrown@ucdavis.edu

Abstract

Background *De novo* transcriptome assemblies are required prior to analyzing RNAseq data from a species without an existing reference genome or transcriptome. Despite the prevalence of transcriptomic studies, the effects of using different workflows, or "pipelines", on the resulting assemblies are poorly understood. Here, a pipeline was programmatically automated and used to assemble and annotate raw transcriptomic short read data collected by the Marine Microbial Eukaryotic Transcriptome Sequencing Project (MMETSP). The resulting transcriptome assemblies were evaluated and compared against assemblies that were previously generated with a different pipeline developed by the National Center for Genome Research (NCGR). **Results** New transcriptome assemblies contained the majority of previous contigs as well as new content. On average, 7.8% of the annotated contigs in the new assemblies were novel gene names not found in the previous assemblies. Taxonomic trends were observed in the assembly metrics, with assemblies from the Dinoflagellata and Ciliophora phyla showing a higher percentage of open reading frames and number of contigs than transcriptomes from other phyla. **Conclusions** Given current bioinformatics approaches, there is no single 'best' reference transcriptome for a particular set of raw data. As the optimum transcriptome is a moving target, improving (or not) with new tools and approaches, automated and programmable pipelines are invaluable for managing the computationally-intensive tasks required for re-processing large sets of samples with revised pipelines and ensuring a common evaluation workflow is applied to all samples. Thus, re-assembling existing data with new tools using automated and programmable pipelines may yield more accurate identification of taxon-specific trends across samples in addition to novel and useful products for the community.

Key words: marine microbial eukaryote; transcriptome assembly; automated pipeline; re-analysis

Introduction

The analysis of gene expression from high-throughput nucleic acid sequence data relies on the presence of a high quality reference genome or transcriptome. When there is no reference genome or transcriptome for an organism of interest, raw RNA sequence data (RNAseq) must be assembled *de novo* into a transcriptome [1]. This type of analysis is ubiquitous across many fields, including: evolutionary developmental biology [2], cancer biology [3], agriculture [4, 5], ecological physiology [6, 7], and biological oceanography [8]. In recent years, substantial investments have been made in data generation, primary data analysis, and development of downstream applications, such as biomarkers and diagnostic tools [9, 10, 11, 12, 13, 14, 15, 16]

Methods for *de novo* RNAseq assembly of the most common short read Illumina sequencing data continue to evolve rapidly, especially for non-model species [17]. At this time, there are several major *de novo* transcriptome assembly software tools available to choose from, including Trinity [18], SOAPdenovo-Trans [19], Trans-ABySS [20], Oases [21], SPAdes [22], IDBA-tran [23], and Shannon [24]. The availability of these options stems from continued research into the unique computational

Compiled on: September 17, 2018.
Draft manuscript prepared by the author.

Key Points

- Re-assembly with new tools can yield new results
- Automated and programmable pipelines can be used to process arbitrarily many samples.
- Analyzing many samples using a common pipeline identifies taxon-specific trends.

challenges associated with transcriptome assembly of short read Illumina RNAseq data, including large memory requirements, alternative splicing and allelic variants [18, 25],

The continuous development of new tools and workflows for RNAseq analysis combined with the vast amount of publicly available RNAseq data [26] raises the opportunity to re-analyze existing data with new tools. This, however, is rarely done systematically. To evaluate the performance impact of new tools on old data, we developed and applied a programmatically automated *de novo* transcriptome assembly workflow that is modularized and extensible based on the Eel Pond Protocol [27]. This workflow incorporates Trimmomatic [28], digital normalization with khmer software [29, 30], and the Trinity *de novo* transcriptome assembler [18].

To evaluate this pipeline, we re-analyzed RNAseq data from 678 samples generated as part of the Marine Microbial Eukaryotic Transcriptome Sequencing Project (MMETSP) [31]. The MMETSP data set was generated to broaden the diversity of sequenced marine protists to enhance our understanding of their evolution and roles in marine ecosystems and biogeochemical cycles [31, 32]. With data from species spanning more than 40 eukaryotic phyla, the MMETSP provides one of the largest publicly-available collections of RNAseq data from a diversity of species. Moreover, the MMETSP used a standardized library preparation procedure and all of the samples were sequenced at the same facility, making this data set unusually comparable.

Reference transcriptomes for the MMETSP were originally assembled by the National Center for Genome Research (NCGR) with a pipeline which used the Trans-ABySS software program to assemble the short reads [31]. The transcriptomes generated from the NCGR pipeline have already facilitated discoveries in the evolutionary history of ecologically significant genes [33, 34], differential gene expression under shifting environmental conditions [8, 35], inter-group transcriptomic comparisons [36], unique transcriptional features [37, 38, 39], and meta-transcriptomic studies [34, 35, 36]

In re-assembling the MMETSP data, we sought to compare and improve the original MMETSP reference transcriptome and to create a platform which facilitates automated re-assembly and evaluation. Here, we show that our re-assemblies had better evaluation metrics and contained most of the NCGR contigs as well as adding new content.

Methods

Programmatically Automated Pipeline

An automated pipeline was developed to execute the steps of the Eel Pond mRNAseq Protocol [27], a lightweight protocol for assembling short Illumina RNAseq reads that uses the Trinity *de novo* transcriptome assembler. This protocol generates *de novo* transcriptome assemblies of acceptable quality [40]. The pipeline was used to assemble all of the data from the MMETSP (Figure 1). The code and instructions for running the pipeline are available at https://doi.org/10.5281/zenodo.740440 [41].

The steps of the pipeline applied to the MMETSP are as follows:

1. Download the raw data

Raw RNA-seq data sets were obtained from the National Center for Biotechnology Information (NCBI) Sequence Read Archive (SRA) from BioProject PRJNA231566. Data were paired-end (PE) Illumina reads with lengths of 50 bases for each read. A metadata (SraRunInfo.csv) file obtained from the SRA web interface was used to provide a list of samples to the get_data.py pipeline script, which was then used to download and extract fastq files from 719 records. The script uses the fastq-dump program from the SRA Toolkit to extract the SRA-formatted fastq files (version 2.5.4) [42]. There were 18 MMETSP samples with more than one SRA record (MMETSP0693, MMETSP1019, MMETSP0923, MMETSP0008, MMETSP1002, MMETSP1325, MMETSP1018, MMETSP1346, MMETSP0088, MMETSP0092, MMETSP0717, MMETSP0223, MMETSP0115, MMETSP0196, MMETSP0197, MMETSP0398, MMETSP0399, MMETSP0922). In these cases, reads from multiple SRA records were concatenated together per sample. Taking these redundancies into consideration, there were a total of 678 re-assemblies generated from the 719 records in PRJNA231566 (Supplemental Notebook 1 [43]). Assembly evaluation metrics were not calculated for MMETSP samples with more than one SRA record because these assemblies were different than the others, containing multiple samples, and thus not as comparable.

Initial transcriptomes that were assembled by the National Center for Genome Resources (NCGR), using methods and data described in the original publication [31], were downloaded from the iMicrobe repository to compare with our re-assemblies (ftp://ftp.imicrobe.us/projects/104/). There were two versions of each assembly, 'nt' and 'cds'. The version used for comparison is noted below in each evaluation step. To our knowledge, the NCGR took extra post-processing steps to filter content, leaving only coding sequences in the 'cds' versions of each assembly [31]

2. Perform quality control

Reads were analyzed with FastQC (version 0.11.5) [44] and multiqc (version 1.2) [45] to confirm overall qualities before and after trimming. A conservative trimming approach [46] was used with Trimmomatic (version 0.33) [28] to remove residual Illumina adapters and cut bases off the start (LEADING) and end (TRAILING) of reads if they were below a threshold Phred quality score (Q<2).

3. Apply digital normalization

To decrease the memory requirements for each assembly, digital normalization was applied with the khmer software package (version 2.0) prior to assembly [47]. First, reads were interleaved, normalized to a k-mer (k = 20) coverage of 20 and a memory size of 4e9, then low-abundance k-mers from reads with a coverage above 18 were trimmed. Orphaned reads, where the mated pair was removed during normalization, were added to the normalized reads.

4. Assemble

Transcriptomes were assembled from normalized reads with Trinity 2.2.0 using default parameters (k = 25). This version of Trinity (2.2.0) did not include the "in silico normalization" op-

tion as a default parameter. The digital normalization approach we used with khmer is the same algorithm implemented in Trinity, but it requires less memory and is faster [48].

The resulting assemblies are referred to below as the "Lab for Data Intensive Biology" assemblies, or DIB assemblies. The original assemblies are referred to as the NCGR assemblies.

5. Post-assembly assessment

Transcriptomes were annotated using the dammit pipeline [49], which relies on the following databases as evidence: Pfam-A (version 28.0) [50], Rfam (version 12.1) [51], OrthoDB (version 8) [52]. In the case where there were multiple database hits, one gene name per contig was selected by choosing the name of the lowest e-value match (<1e-05).

All assemblies were evaluated using metrics generated by the Transrate program [53]. Trimmed reads were used to calculate a Transrate score for each assembly, which represents the geometric mean of all contig scores multiplied by the proportion of input reads providing positive support for the assembly [50]. Comparative metrics were calculated using Transrate for each MMETSP sample between DIB and the NCGR assemblies using the Conditional Reciprocal Best BLAST hits (CRBB) algorithm [54]. A forward comparison was made with the NCGR assembly used as the reference and each DIB re-assembly as the query. Reverse comparative metrics were calculated with each DIB re-assembly as the reference and the NCGR assembly as the query. Transrate scores were calculated for each assembly using the Trimmomatic quality-trimmed reads, prior to digital normalization.

Benchmarking Universal Single-Copy Orthologs (BUSCO) software (version 3) was used with a database of 215 orthologous genes specific to protistans and 303 genes specific to eukaryota with open reading frames in the assemblies. BUSCO scores are frequently used as one measure of assembly completeness [55]

To assess the occurrences of fixed-length words in the assemblies, unique 25-mers were measured in each assembly using the HyperLogLog (HLL) estimator of cardinality built into the khmer software package [56]. We used the HLL function to digest each assembly and count the number of distinct fixed-length substrings of DNA (k-mers).

Unique gene names were compared from a random subset of 296 samples using the dammit annotation pipeline [49]. If a gene name was annotated in NCGR but not in DIB, this was considered a gene uniquely annotated in NCGR. Unique gene names were normalized to the total number of annotated genes in each assembly.

Table 1. Number of assemblies with higher values in NCGR or DIB for each quality metric. The "cds" or "nt" indicate the version of the NCGR assembly compared with. The NCGR "cds" assemblies were filtered for ORF content.

Quality Metric	Higher in NCGR	Higher in DIB
Transrate score, "cds"	44	583
Transrate score, "nt"	495	143
Mean ORF %, "cds"	592	35
Mean ORF %, "nt"	42	596
% References with CRBB, "nt"	100	538
Number of contigs, "nt"	12	626
% Complete BUSCO, Eukaryota, "nt"	381	235

A Tukey's honest significant different (HSD) post-hoc range test of multiple pairwise comparisons was used in conjunction with an ANOVA to measure differences between distributions of data from the top eight most-represented phyla ("Bacillariophyta", "Dinophyta", "Ochrophyta", "Haptophyta", "Ciliophora", "Chlorophyta", "Cryptophyta", "Others") using the 'agricolae' package version 1.2-8 in R version 3.4.2 (2017-09-28). Margins sharing a letter in the group label are not significantly different at the 5% level (8). Averages are reported ± standard deviation.

Results

After assemblies and annotations were completed, files were uploaded to Figshare and Zenodo are available for download [57]. Due to obstacles encountered uploading and maintaining 678 assemblies on Figshare, Zenodo will be the long-term archive for these re-assemblies http://doi.org/10.5281/zenodo.1212585. Assembly quality metrics were summarized and are available (Supplemental Tables 1 and 2 [43]).

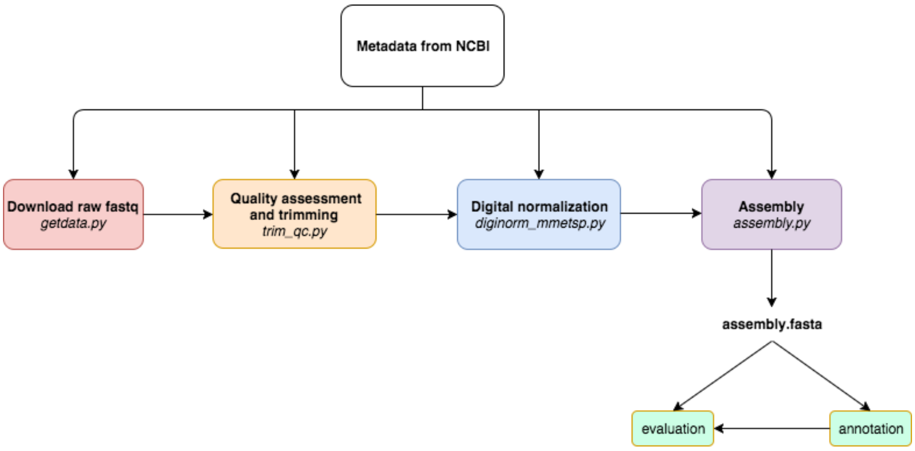

Figure 1. A programmatically automated *de novo* transcriptome assembly pipeline was developed for this study. Metadata in the SraRunInfo.csv file downloaded from NCBI was used as input for each step of the pipeline to indicate which samples were processed. The steps of the pipeline are as follows: download raw fastq data with the fastq-dump script in the SRA Toolkit, perform quality assessment with FastQC and trim residual Illumina adapters and low quality bases (Q<2) with Trimmomatic, do digital normalization with khmer version 2.0, and perform *de novo* transcriptome assembly with Trinity. If a process was terminated, the automated nature of this pipeline allowed for the last process to be run again without starting the pipeline over. In the future, if a new sample is added, the pipeline can be run from beginning to end with just new samples, without having to repeat the processing of all samples in the dataset as one batch. If a new tool becomes available, for example a new assembler, it can be substituted in lieu of the original tool used by this pipeline.

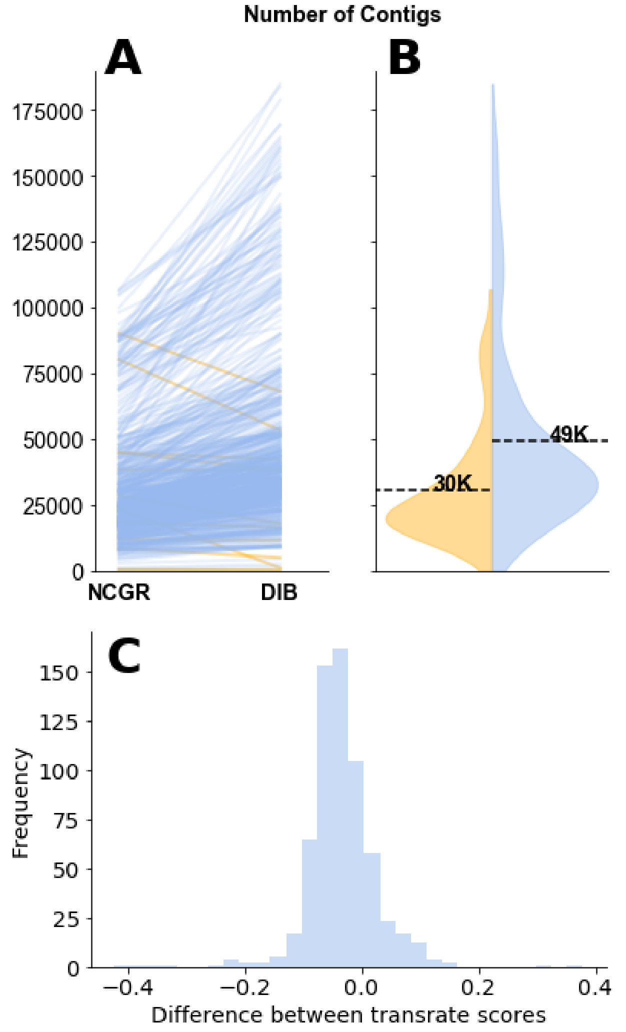

Figure 2. The number of contigs and Transrate quality score for each data set varied between DIB and NCGR assemblies. (A) Slopegraphs show shifts in the number of contigs for each individual sample between the DIB and the NCGR assembly pipelines. Negative slope (yellow) lines represent values where NCGR was higher than DIB and positive slope (blue) lines represent values where DIB was higher than NCGR. (B) Split violin plots show the distribution of the number of contigs in each assembly with the original assemblies from NCGR in yellow (left) and the DIB re-assemblies and in blue (right side of B). (C) The difference in Transrate score between the DIB and NCGR assemblies is shown as a histogram. Negative values on the x-axis indicate that the NCGR assembly had a higher Transrate score and positive values indicate that the DIB assembly had a higher Transrate score.

Differences in available evaluation metrics between NCGR and DIB were variable.

The majority of transcriptome evaluation metrics collected for each sample were higher in Trinity-based DIB re-assemblies than for the Trans-ABySS-based NCGR assemblies, 'cds' versions (1). The Transrate score from the "nt" version of the assemblies were higher in NCGR vs. DIB, whereas compared to the 'cds' version, the DIB re-assemblies were higher (Supplemental Figure 1 [43]). Since the NCGR 'cds' assemblies were filtered for open reading frame (ORF) content, and the DIB re-assemblies were not filtered, the unfiltered NCGR 'nt' assemblies are more comparable to the DIB re-assemblies.

The DIB re-assemblies had more contigs than the NCGR assemblies in 83.5% of the samples (1). The mean number of contigs in the DIB re-assemblies was 48,361 ± 35,703 while the mean number of contigs in the NCGR 'nt' assemblies was 30,532 ± 21,353 (2). A two-sample Kolmogorov-Smirnov test comparing distributions indicated that the number of contigs were significantly different between DIB and NCGR assemblies ($p < 0.001$, D = 0.35715). Transrate scores [53], which calculate the overall quality of the assembly based on the original reads, were significantly higher in the DIB re-assemblies (0.31 ± 0.1) compared to the 'cds' versions of the NCGR assemblies (0.22 ± 0.09) ($p < 0.001$, D = 0.49899). The Transrate scores in the NCGR 'nt' assemblies (0.35 ± 0.09) were significantly higher than the DIB assemblies (0.22 ± 0.09) ($p < 0.001$, D = 0.22475) (Supplemental Figure 1 [43]). The frequency of the differences between Transrate scores in the NCGR 'nt' assemblies and the DIB re-assemblies is centered around zero (Figure 2C). Transrate scores from the DIB assemblies relative to the NCGR 'nt' assemblies did not appear to have taxonomic trends (Supplemental Figure 2 [43]

The DIB re-assemblies contained most of the NCGR contigs as well as new content.

We applied CRBB to evaluate overlap between the assemblies. A positive CRBB result indicates that one assembly contains the same contig information as the other. Thus, the proportion of positive CRBB hits can be used as a scoring metric to compare the relative similarity of content between two assemblies. For example, MMETSP0949 (*Chattonella subsalsa*) had 39,051 contigs and a CRBB score of 0.71 in the DIB re-assembly whereas in the NCGR assembly of the same sample had 18,873 contigs and a CRBB score of 0.34. This indicated that 71% of the reference of DIB was covered by the NCGR assembly, whereas in the reverse alignment, the NCGR reference assembly was only covered by 34% of the DIB re-assembly. The mean CRBB score in DIB when queried against NCGR 'nt' as a reference was 0.70 ± 0.22, while the mean proportion for NCGR 'nt' assemblies queried against DIB re-assemblies was 0.49 ± 0.10 ($p < 0.001$, D = 0.71121) (3). This indicates that more content from the NCGR assemblies was included in the DIB re-assemblies than vice versa and also suggests that the DIB re-assemblies overall have additional content. This finding is reinforced by higher

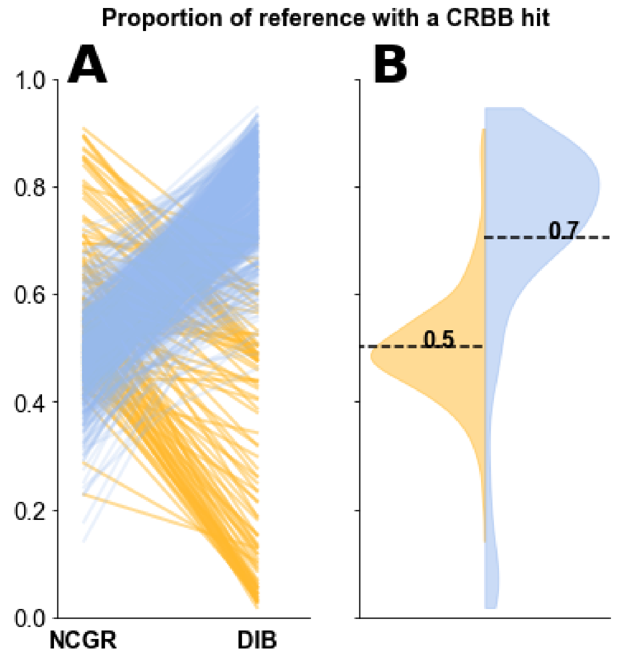

Figure 3. (A) Slopegraphs comparing the proportion of CRBB hits between NCGR 'nt' assemblies and DIB assemblies between the same samples. (B) Violin plots showing the distribution of the proportion of NCGR transcripts with reciprocal BLAST hits to DIB (blue) and the proportion of DIB transcripts with reciprocal BLAST hits to NCGR (yellow).

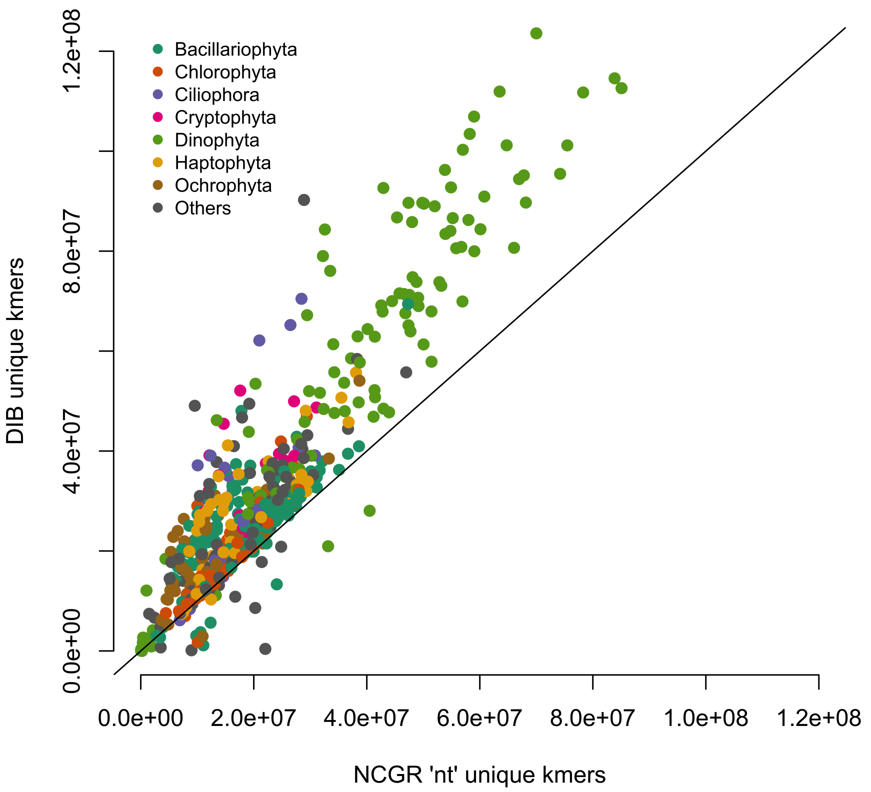

Figure 4. Unique numbers of k-mers (k=25) in seven most represented phyla, calculated with the HyperLogLog function in the khmer software package. DIB re-assemblies were compared to the NCGR 'nt' assemblies along a 1:1 line. Samples are colored based on their phylum level affiliation. More than 95% of the DIB re-assemblies had more unique k-mers than to the NCGR assembly of the same sample.

unique k-mer content found in the DIB re-assemblies compared to NCGR, where more than 95% of the samples had more unique k-mers in the DIB re-assemblies compared to NCGR assemblies (4).

To investigate whether the new sequence content was genuine, we examined two different metrics that take into account the biological quality of the assemblies. First, the estimated content of open reading frames (ORFs), or coding regions, across contigs was quantified. Though DIB re-assemblies had more contigs, the ORF content is similar to the original assemblies, with a mean of 81.8 ± 9.9% ORF content in DIB re-assemblies and 76.7 ± 10.1% ORF content in the NCGR assemblies. Nonetheless, ORF content in DIB re-assemblies was higher than NCGR assemblies for 95% of the samples (5), although DIB re-assemblies had significantly higher ORF content ($p < 0.001$, $D = 2681$). Second, when the assemblies were queried against the eukaryotic BUSCO database [55], the percentages of BUSCO eukaryotic matches in the DIB re-assemblies (61.8 ± 19.9%) were similar to the original NCGR assemblies (63.8 ± 20.3%) (5). However, the DIB re-assemblies were significantly different compared to the NCGR assemblies ($p = 0.002408$, $D = 0.099645$). Therefore, although the number of contigs and amount of CRBB content were dramatically increased in the DIB re-assemblies compared to the NCGR assemblies, the differences in ORF content and BUSCO matches compared to eukaryotic (5) and protistan (Supplemental Figure 3 [43]) databases, while they were significantly different, were less dramatic. This suggests that content was not lost by gaining extra contigs. Since the extra content contained roughly similar proportions of ORFs and BUSCO annotations, it is likely that the re-assemblies contribute more biologically meaningful information.

Looking through the results for missing BUSCOs in the eight samples where NCGR had >30% higher complete BUSCO evaluation score (MMETSP0121, MMETSP0932, MMETSP0045, MMETSP0169, MMETSP0232, MMETSP0439, MMETSP0329, MMETSP0717), in some cases a particular orthogroup in the BUSCO database does not produce output for reasons that we don't understand. For example, the Trinity-based pipeline only produced 342 contigs for sample MMETSP0232 while the NCGR 'nt' assembly had 4234 and the 'cds' version had 2736. BUSCO did not recognize any of the DIB contigs but it did recognize the NCGR contigs. For other samples, MMETSP0169 (*Corethron pennatum*, Phylum: Bacillariophyta), the BUSCO software recognized several DIB contigs but the BUSCO group was still considered "missing", even though there were lengths of the contig identified in the output as being similar. For example, the BUSCO orthogroup "EOG0937060I" is a "DNARNA helicase, ATP-dependent, DEAH-box type, conserved site". The BUSCO output indicates the DIB contig, "TRINITY_DN13758_c3_g2_i1" with a length 974 bases is related to this orthogroup. When we look for this gene in the gff annotation file for MMETSP0169, there are no annotation results for this contig. Another DIB contig, "TRINITY_DN3716_c0_g1_i1" (length 154) is also identified as similar to this same orthogroup. This contig does have annotation results, but it matches with a BUSCO orthogroup, "EOG090C08EI", which is a different gene, Abl-interactor, homeo-domain homologous domain (ABI family, member 3a). The top results comparing the contig

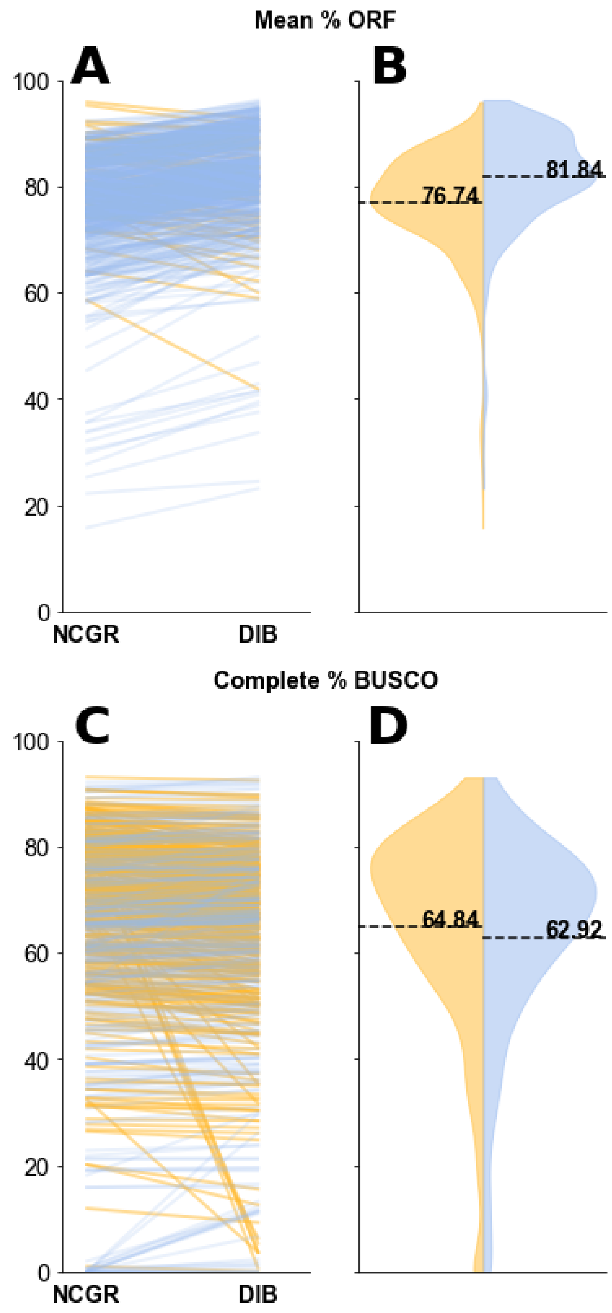

Figure 5. The percentage of contigs with a predicted open reading frame (ORF) (A, B) and the percentage of complete protistan universal single-copy orthologs (BUSCO) recovered in each assembly (C, D). In the blue (right side B, D) are the “DIB” re-assemblies and in yellow (left side of B, D) are the original ‘nt’ assemblies from NCGR. Slopegraphs (A,C) compare values between the DIB and the NCGR ‘nt’ assemblies. Yellow lines represent negative slope values where NCGR was higher than DIB and blue lines represent positive slope values where DIB was higher than NCGR.

sequence, "TRINITY_DN13758_c3_g2_i1" against the NCBI blastn database matches with small, several hundred bp regions of the EOG0937060I gene sequence (XM_021257656.1, XM_004843976.2, XM_010604294.2, XM_010604293.2, XM_010604291.2, XR_776390.2). Even though this contig was assembled, it did not successfully annotate. We do not know whether there are errors associated with this assembled contig, or if the contig sequence is unique to this MMETSP0169 organism. Since the BUSCO database and corresponding orthogroups were constructed from multiple sequence alignments with existing individuals in the databases, it is possible that the transcriptome from the newly sequenced, MMETSP0169 (*Corethron pennatum*) may naturally fall outside the hmm scoring cutoffs for matching with the BUSCO orthogroups. Since the corresponding NCGR assembly had a “Duplicated” result from this particular BUSCO, it is also possible that there is a particular oddity within this ortholog.

There are many examples that can be picked over in these results, which suggests that there is more to learn about the evaluation tools within the context of the organisms in this data set. For now, we conclude that our assemblies are differently fragmented in some regions relative to the NCGR assemblies. We have assembled additional sequences that were not assembled by NCGR. Some NCGR assemblies had different and more complete content than the DIB assemblies. As far as we can tell, there does not appear to be a pattern in the samples that fared well with this pipeline vs. NCGR. This could be a future avenue to explore.

Following annotation by the dammit pipeline [49], 91 ± 1.6% of the contigs in the DIB re-assemblies had positive matches with sequence content in the databases queried (Pfam, Rfam, and OrthoDB), with 48 ± 0.9% of those containing unique gene names (the remaining are fragments of the same gene). Of those annotations, 7.8 ± 0.2% were identified as novel compared to the NCGR ‘nt’ assemblies, determined by a “false” CRBB result (6). Additionally, the number of unique gene names in DIB re-assemblies were higher in 97% of the samples compared to NCGR assemblies, suggesting an increase in genic content (7).

Novel contigs in the DIB re-assemblies likely represent a combination of unique annotations, allelic variants and alternatively spliced isoforms. For example, "F0XV46_GROCL", "Helicase_C", "ODR4-like","PsaA_PsaB", and "Metazoa_SRP" are novel gene names found annotated in the DIB re-assembly of the sample MMETSP1473 (Stichococcus sp.) that were absent in the NCGR assembly of this same sample. Other gene names,

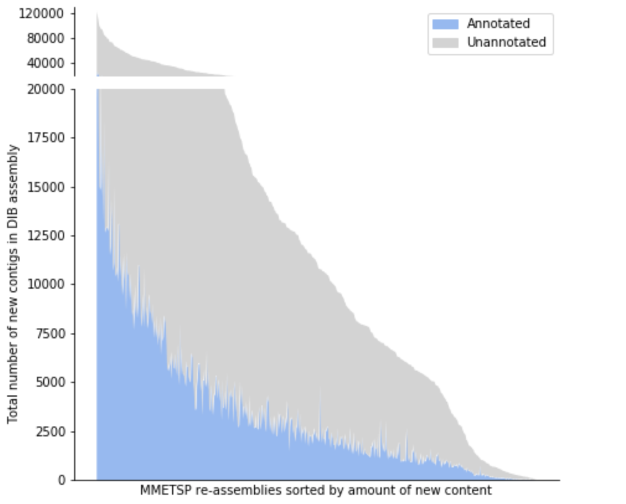

Figure 6. A histogram across MMETSP samples depicting the number of contigs identified as novel in DIB assemblies. These contigs were absent in the NCGR assemblies, based on negative conditional reciprocal best BLAST (CRBB) results. Samples are sorted from highest to lowest number of ‘new’ contigs. The region in gray indicates the number of unannotated contigs present in the DIB re-assemblies, absent from NCGR ‘nt’ assemblies. Highlighted in blue are contigs that were annotated with dammit [49] to a gene name in the Pfam, Rfam, or OrthoDB databases, representing the number of contigs unique to the DIB re-assemblies with an annotation.

for example "Pkinase_Tyr","Bromodomain", and "DnaJ", are found in both the NCGR and DIB assemblies, but are identified as novel contigs based on negative CRBB results in the DIB re-assembly of sample MMETSP1473 compared to the NCGR reference.

Assembly metrics varied by taxonomic group being assembled. To examine systematic taxonomic differences in the assemblies, metrics for content and assembly quality were assessed (8). Metrics were grouped by the top eight most represented phyla in the MMETSP data set as follows: Bacillariophyta (N=173), Dinophyta (N=114), Ochrophyta (N=73), Chlorophyta (N=62), Haptophyta (N=61), Ciliophora (N=25), Cryptophyta (N=22) and Others (N=130).

While there were no major differences between the phyla in the number of input reads (Figure 8 A), the Dinoflagellates (Dinophyta) had significantly different (higher) number of contigs ($p < 0.01$), unique k-mers ($p < 0.001$), and % ORF ($p < 0.001$) compared to other groups (8), and assemblies from Ciliates (Ciliophora) had lower % ORF ($p < 0.001$) (8).

Discussion

DIB re-assemblies contained the majority of the previously-assembled contigs. We used a different pipeline than the original one used to create the NCGR assemblies, in part because new software was available [18] and in part because of new trimming guidelines [46]. The general genome assembler ABySS [20] was used in conjunction with a *de novo* transcriptome assembly pipeline described by Keeling et al. [31]. We had no a priori expectation for the similarity of the results, yet we found that the majority of new DIB re-assemblies included substantial portions of the previous NCGR assemblies seen in the CRBB results. Given this, it may seem surprising that the Transrate and BUSCO scores are lower in the DIB re-assemblies relative to the NCGR counterparts. However, given that the number of contigs and the k-mer content were both dramatically increased in the DIB re-assemblies, it is interesting that the ORFs and annotations were similar between the two assemblies. If the extra content observed was due to assembly artifact, we would not expect these content-based results to be similar. The two metrics, Transrate and BUSCO, which estimate “completeness” of the transcriptomes, may not be telling the whole story. Our results suggest that both pipelines yielded similarly valid contigs, even though the NCGR assemblies appeared to be less sensitive.

The relative increase in number of unique k-mers from the NCGR assemblies to the DIB re-assemblies could be due to the higher number of contigs generated by Trinity. Within the data, the Trinity assembler found evidence for building alternative isoforms. The ABySS assembler and transcriptome pipeline that NCGR used [31] appears to not have preserved that variation, perhaps in an attempt to narrow down the contigs to a consensus transcript sequence.

Re-assembly with new tools can yield new results Evaluation with quality metrics suggested that the DIB re-assemblies were more inclusive than the NCGR assemblies. The Transrate scores in the DIB re-assemblies compared to the NCGR ‘nt’ assemblies were significantly lower, indicating that the NCGR ‘nt’ assemblies had better overall read inclusion in the assembled contigs whereas the DIB assemblies had higher Transrate scores than the NCGR ‘cds’ version. This suggests that the NCGR ‘cds’ version, which was post-processed to only include coding sequence content, was missing information originally in the quality-trimmed reads. As we also saw with % ORF, when filtration steps select only for ORF content in the NCGR ’cds’ versions, potentially useful content is lost. The Transrate score [53] is one of the few metrics available for evaluating the ‘quality’ of a *de novo* transcriptome. It is similar to the DETONATE RSEM-EVAL score in that it returns a metric indicating how well the assembly is supported by the read data [13]. It does not directly evaluate the underlying de Bruijn graph data structure used to produce the assembled contigs. In the future, metrics directly evaluating the underlying de Bruijn graph data structure may better evaluate assembly quality. Here, the DIB re-assemblies, which used the Trinity *de novo* assembly software, typically contained more k-mers, more annotated transcripts, and more unique gene names than the NCGR assemblies.

These points all suggest that additional content in these re-assemblies might be biologically relevant and that these re-assemblies provide new content not available in the previous NCGR assemblies. Since contigs are probabilistic predictions of full-length transcripts made by assembly software [18], ‘final’ reference assemblies are approximations of the full set of transcripts in the transcriptome. Results from this study suggest that the ‘ideal’ reference transcriptome is a moving target and that these predictions may continue to improve given updated tools in the future.

For some samples, complete BUSCO scores were lower than over half of DIB vs. NCGR. This could be an effect of the BUSCO metric, given that these samples did not perform poorly with other metrics such as % ORF and number of contigs compared to the NCGR. For other samples, MMETSP0252 (*Prorocentrum lima*) in particular, assemblies required several tries and only four contigs were assembled from 30 million reads. The fastqc reports were unremarkable, compared to the other samples. In such a large dataset with a diversity of species with no prior sequencing data it is challenging to speculate why each anomaly occurred. However, further investigation into the reasons for failures and peculiarities in the evaluation metrics may lead to interesting discoveries about how we should be effectively assembling and evaluating nucleic acid sequencing data from a diversity of species.

We predict that assembly metrics could have been further improved with longer read lengths of the original data since MMETSP data had only 50 bp read lengths, although this would have presented Keeling et al. [31] with a more expensive data collection endeavor. A study by Chang et al. [25] reported a consistent increase in the percentage of full-length transcript reconstruction and a decrease in the false positive rate moving from 50 to 100 bp read lengths with the Trinity assembler. However, regardless of length, the conclusions we draw would likely remain the same that assembling data with new tools can yield new results.

The DIB re-assemblies, including the additional biologically relevant information, are likely to be meta-transcriptomes. RNA sequences generated from the MMETSP experiments are likely to contain genetic information from more than the target species, as many were not or could not be cultured axenically. Thus, both the NCGR assemblies and the DIB re-assemblies, including the additional biologically relevant information, might be considered meta-transcriptomes. Sequencing data and unique k-mer content likely include bacteria, viruses, or other protists that occurred within the sequenced sample. We did not make an attempt to de-contaminate the assemblies.

The evaluation metrics described here generally serve as a framework for better contextualizing the quality of protistan transcriptomes. For some species and strains in the MMETSP data set, these data represent the first nucleic acid sequence information available [31].

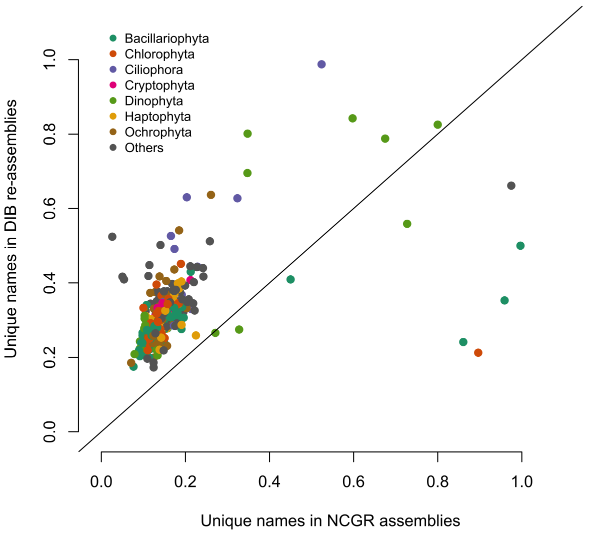

Figure 7. Unique gene names found in a subset (296 samples) of either NCGR 'nt' assemblies or DIB re-assemblies but not found in the other assembly, normalized to the number of annotated contigs in each assembly. The line indicates a 1:1 relationship between the unique gene names in DIB and NCGR. More than 97% of the DIB assemblies had more unique gene names than in NCGR assemblies of the same sample.

Automated and programmable pipelines can be used to process arbitrarily many RNAseq samples.

The automated and programmable nature of this pipeline was useful for processing large data sets like the MMETSP as it allowed for batch processing of the entire collection, including re-analysis when new tools or new samples become available (see op-ed Alexander et al. 2018). During the course of this project, we ran two re-assemblies of the complete MMETSP data set and one subset as new versions Trinity were released (Supplemental Notebook 2 [43]). Each re-analysis of the complete dataset required only a single command and approximately half a CPU-year of compute. The value of automation is clear when new data from samples become available to expand the data set, tools are updated, or many tools are compared in benchmark studies. Despite this, few assembly efforts completely automate their process, perhaps because the up-front cost of doing so is high compared to the size of the dataset typically being analyzed.

For the purposes of future benchmarking studies, a subset of 12 "High" and 15 "Low" performing samples were identified based on the evaluation metrics: number of contigs, longest contig length, unique k-mers (k=25), and % Complete BUSCO (eukaryota) (Supplemental Figure 4 [43]).

Analyzing many samples using a common pipeline identifies taxon-specific trends.

The MMETSP dataset presents an opportunity to examine transcriptome qualities for hundreds of taxonomically diverse species spanning a wide array of protistan lineages. This is among the largest set of diverse RNAseq data to be sequenced. In comparison, the Assemblathon2 project compared genome assembly pipelines using data from three vertebrate species [58]. The BUSCO paper assessed 70 genomes and 96 transcriptomes representing groups of diverse species (vertebrates, arthropods, other metazoans, fungi) [55]. Other benchmarking studies have examined transcriptome qualities for samples representing dozens of species from different taxonomic groupings [59, 60]. A study with a more restricted evolutionary analysis of 15 plant and animals species [60] found no evidence of taxonomic trends in assembly quality but did find evidence of differences between assembly software packages [59].

With the MMETSP data set, we show that comparison of assembly evaluation metrics across this diversity provides not only a baseline for assembly performance, but also highlights particular metrics which are unique within some taxonomic groups. For example, the phyla Ciliophora had a significantly lower percentage of ORFs compared to other phyla. This is supported by recent work which has found that ciliates have an alternative triplet codon dictionary, with codons normally encoding STOP serving a different purpose [37, 38, 39], thus application of typical ORF finding tools fail to identify ORFs accurately in Ciliophora. Additionally, Dinophyta data sets had a significantly higher number of unique k-mers and total contigs in assemblies compared to the assemblies from other data sets, despite having the same number of input reads. Such a finding supports previous evidence from studies showing that large gene families are constitutively expressed in Dinophyta [61].

In future development of *de novo* transcriptome assembly software, the incorporation of phylum-specific information may be useful in improving the overall quality of assemblies for different taxa. Phylogenetic trends are important to consider in the assessment of transcriptome quality, given that the assemblies from Dinophyta and Ciliophora are distinguished from other assemblies by some metrics. Applying domain-specific knowledge, such as specialized transcriptional features in a given phyla, in combination with other evaluation metrics can help to evaluate whether a transcriptome is of good quality or "finished" enough to serve as a high quality reference to answer the biological questions of interest.

Conclusion

As the rate of sequencing data generation continues to increase, efforts to programmatically automate the processing and evaluation of sequence data will become increasingly important. Ultimately, the goal in generating *de novo* transcriptomes is to create the best possible reference against which downstream analyses can be accurately based. This study demonstrated that re-analysis of old data with new tools and methods improved the quality of the reference assembly through an expansion of the gene catalog of the dataset. Notably, these improvements arose without further experimentation or sequencing.

With the growing volume of nucleic acid data in centralized and decentralized repositories, streamlining methods into pipelines will not only enhance the reproducibility of future analyses, but will facilitate inter-comparisons amongst from both similar and diverse datasets. Automation tools were key in successfully processing and analyzing this large collection of 678 samples.

Acknowledgements

The authors would gratefully like to acknowledge the contributions of Camille Scott (0000-0001-8822-8779), Luiz Irber (0000-0003-4371-9659), Taylor Reiter (0000-0002-7388-421X), Daniel Standage (0000-0003-0342-8531) and other members of the Data Intensive Biology lab at UC Davis for providing their time towards helpful assistance with troubleshooting the assembly, annotation and evaluation pipeline and visualizations presented in this paper.

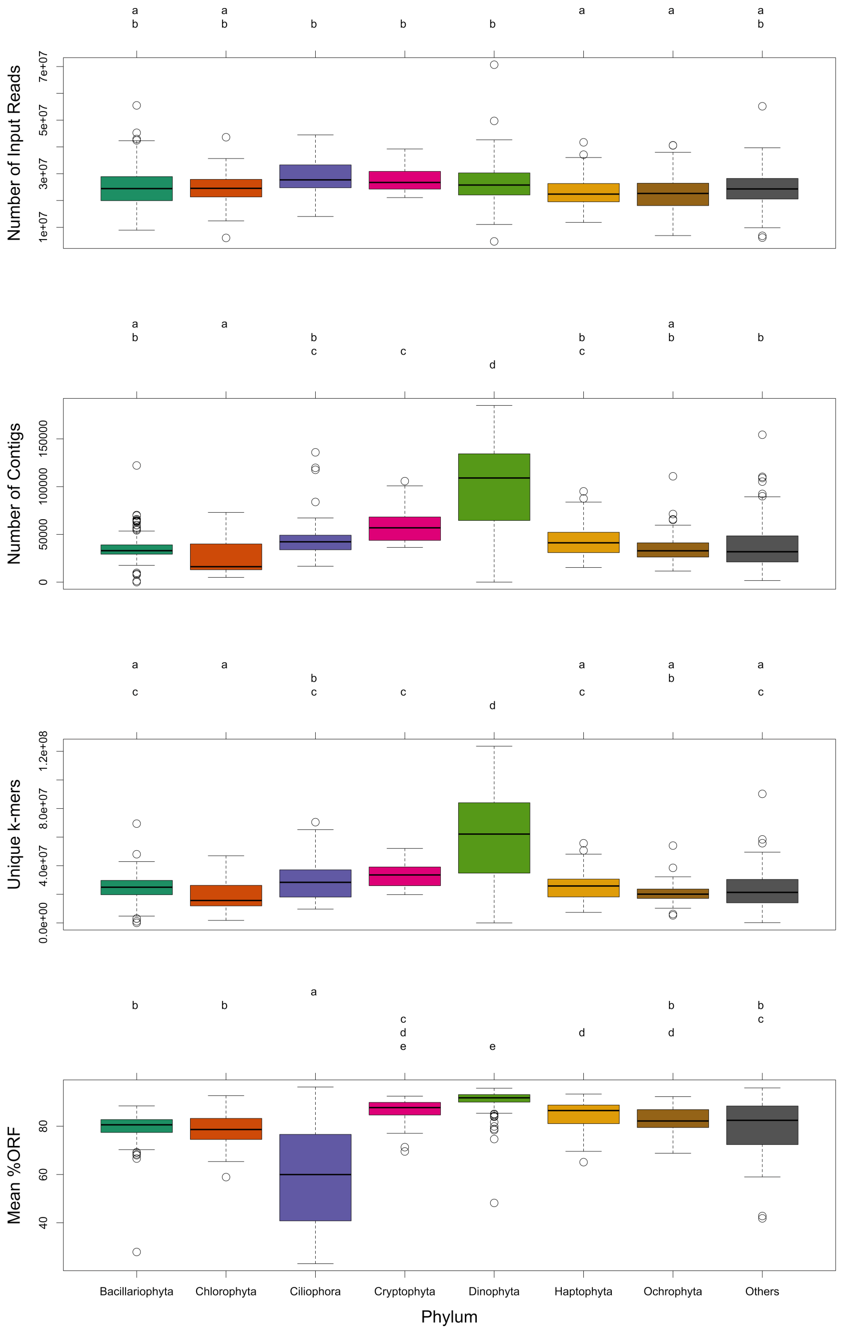

Figure 8. Box-and-whisker plots for the seven most common phyla in the MMETSP dataset, (A) number of input reads, (B) number of contigs in the assembly, (C) unique k-mers (k = 25) in the assembly, (D) mean percentage open reading frames (ORF). Groups sharing a letter in the top margin were generated from Tukey's HSD post-hoc range test of multiple pairwise comparisons used in conjunction with an ANOVA.

Declarations

List of abbreviations

- BLAST = Basic Local Alignment Search Tool
- CRBB = Conditional Recriprocal Best BLAST
- DIB = Data Intensive Biology Lab at the University of California Davis
- HLL = HyperLogLog
- MMETSP = Marine Microbial Eukaryotic Transcriptome Sequencing Project
- NCGR = National Center for Genome Research
- ORF = Open Reading Frame
- NCBI = National Center for Biotechnology Information
- SRA = Sequence Read Archive

Ethical Approval

Data were downloaded from public repositories, provided by [31] and NCBI BioProject PRJNA231566 as cited in the text.

Consent for publication

Not applicable.

Competing Interests

The authors declare that they have no competing interests.

Funding

Funding was provided from the Gordon and Betty Moore Foundation under award number GBMF4551 to CTB. Scripts were tested and run on the MSU HPCC and NSF-XSEDE Jetstream cloud platform with allocation TG-BIO160028 [62, 63].

Author's Contributions

Re-assembly, automated analysis pipeline, and data curation were done by LKJ. Writing, review & editing were done by LKJ, HA, CTB. Visualizations were developed by HA and LKJ.

References

1. Geniza M, Jaiswal P. Tools for building de novo transcriptome assembly. Current Plant Biology 2017 sep;11-12:41–45. https://doi.org/10.1016%2Fj.cpb.2017.12.004.
2. Tulin S, Aguiar D, Istrail S, Smith J. A quantitative reference transcriptome for Nematostella vectensis early embryonic development: a pipeline for de novo assembly in emerging model systems. EvoDevo 2013;4(1):16. https://doi.org/10.1186%2F2041-9139-4-16.
3. Mittal VK, McDonald JF. De novo assembly and characterization of breast cancer transcriptomes identifies large numbers of novel fusion-gene transcripts of potential functional significance. BMC Medical Genomics 2017 aug;10(1). https://doi.org/10.1186%2Fs12920-017-0289-7.
4. SONG Y, di LIU H, ZHOU Q, jun ZHANG H, dong ZHANG Z, dong LI Y, et al. High-throughput sequencing of highbush blueberry transcriptome and analysis of basic helix-loop-helix transcription factors. Journal of Integrative Agriculture 2017 mar;16(3):591–604. https://doi.org/10.1016%2Fs2095-3119%2816%2961461-2.
5. Suárez-Vega A, Gutiérrez-Gil B, Klopp C, Tosser-Klopp G, Arranz JJ. Comprehensive RNA-Seq profiling to evaluate lactating sheep mammary gland transcriptome. Scientific Data 2016 jul;3:160051. https://doi.org/10.1038%2Fsdata.2016.51.
6. Carruthers M, Yurchenko AA, Augley JJ, Adams CE, Herzyk P, Elmer KR. De novo transcriptome assembly, annotation and comparison of four ecological and evolutionary model salmonid fish species. BMC Genomics 2018 jan;19(1). https://doi.org/10.1186%2Fs12864-017-4379-x.
7. Mansour TA, Rosenthal JJC, Brown CT, Roberson LM. Transcriptome of the Caribbean stony coral Porites astreoides from three developmental stages. GigaScience 2016 aug;5(1). https://doi.org/10.1186%2Fs13742-016-0138-1.
8. Frischkorn KR, Harke MJ, Gobler CJ, Dyhrman ST. De novo assembly of Aureococcus anophagefferens transcriptomes reveals diverse responses to the low nutrient and low light conditions present during blooms. Frontiers in Microbiology 2014 jul;5. https://doi.org/10.3389%2Ffmicb.2014.00375.
9. Mansour TA, Scott EY, Finno CJ, Bellone RR, Mienaltowski MJ, Penedo MC, et al. Tissue resolved, gene structure refined equine transcriptome. BMC Genomics 2017 jan;18(1). https://doi.org/10.1186%2Fs12864-016-3451-2.
10. Gonzalez VL, Andrade SCS, Bieler R, Collins TM, Dunn CW, Mikkelsen PM, et al. A phylogenetic backbone for Bivalvia: an RNA-seq approach. Proceedings of the Royal Society B: Biological Sciences 2015 jan;282(1801):20142332–20142332. https://doi.org/10.1098%2Frspb.2014.2332.
11. Müller M, Seifert S, Lübbe T, Leuschner C, Finkeldey R. De novo transcriptome assembly and analysis of differential gene expression in response to drought in European beech. PLOS ONE 2017 sep;12(9):e0184167. https://doi.org/10.1371%2Fjournal.pone.0184167.
12. Heikkinen LK, Kesäniemi JE, Knott KE. De novo transcriptome assembly and developmental mode specific gene expression of Pygospio elegans. Evolution & Development 2017 jul;19(4-5):205–217. https://doi.org/10.1111%2Fede.12230.
13. Li F, Wang L, Lan Q, Yang H, Li Y, Liu X, et al. RNA-Seq Analysis and Gene Discovery of Andrias davidianus Using Illumina Short Read Sequencing. PLOS ONE 2015 apr;10(4):e0123730. https://doi.org/10.1371%2Fjournal.pone.0123730.
14. Yu J, Lou Y, Zhao A. Transcriptome analysis of follicles reveals the importance of autophagy and hormones in regulating broodiness of Zhedong white goose. Scientific Reports 2016 nov;6(1). https://doi.org/10.1038%2Fsrep36877.
15. Seo M, Kim K, Yoon J, Jeong JY, Lee HJ, Cho S, et al. RNA-seq analysis for detecting quantitative trait-associated genes. Scientific Reports 2016 apr;6(1). https://doi.org/10.1038%2Fsrep24375.
16. Pedrotty DM, Morley MP, Cappola TP. Transcriptomic Biomarkers of Cardiovascular Disease. Progress in Cardiovascular Diseases 2012 jul;55(1):64–69. https://doi.org/10.1016%2Fj.pcad.2012.06.003.
17. Conesa A, Madrigal P, Tarazona S, Gomez-Cabrero D, Cervera A, McPherson A, et al. A survey of best practices for RNA-seq data analysis. Genome Biology 2016 jan;17(1). https://doi.org/10.1186%2Fs13059-016-0881-8.
18. Grabherr MG, Haas BJ, Yassour M, Levin JZ, Thompson DA, Amit I, et al. Full-length transcriptome assembly from RNA-Seq data without a reference genome. Nature Biotechnology 2011 may;29(7):644–652. https://doi.org/10.1038%2Fnbt.1883.
19. Xie Y, Wu G, Tang J, Luo R, Patterson J, Liu S, et al. SOAPdenovo-Trans: de novo transcriptome assembly with short RNA-Seq reads. Bioinformatics 2014 feb;30(12):1660–1666. https://doi.org/10.1093%2Fbioinformatics%2Fbtu077.
20. Robertson G, Schein J, Chiu R, Corbett R, Field M, Jackman SD, et al. De novo assembly and analysis of RNA-seq data. Nature Methods 2010 oct;7(11):909–912. https://doi.org/10.1038%2Fnmeth.1517.
21. Schulz MH, Zerbino DR, Vingron M, Birney E. Oases: robust de novo RNA-seq assembly across the dynamic range of expression levels. Bioinformatics 2012 feb;28(8):1086–1092. https://doi.org/10.1093%2Fbioinformatics%2Fbts094.
22. Bankevich A, Nurk S, Antipov D, Gurevich AA, Dvorkin M, Kulikov AS, et al. SPAdes: A New Genome Assembly Algorithm and Its Applications to Single-Cell Sequencing. Journal of Computational Biology 2012 may;19(5):455–477. https://doi.org/10.1089%2Fcmb.2012.0021.
23. Peng Y, Leung HCM, Yiu SM, Lv MJ, Zhu XG, Chin FYL. IDBA-tran: a more robust de novo de Bruijn graph assembler for transcriptomes with uneven expression levels. Bioinformatics 2013 jun;29(13):i326–i334. https://doi.org/10.1093%2Fbioinformatics%2Fbtt219.
24. Kannan S, Hui J, Mazooji K, Pachter L, Tse D. Shannon: An Information-Optimal de Novo RNA-Seq Assembler. bioRxiv 2016;https://www.biorxiv.org/content/early/2016/02/09/039230.
25. Chang Z, Wang Z, Li G. The Impacts of Read Length and Transcriptome Complexity for De Novo Assembly: A Sim-

ulation Study. PLoS ONE 2014 apr;9(4):e94825. https://doi.org/10.1371%2Fjournal.pone.0094825.
26. Solomon B, Kingsford C. Fast search of thousands of short-read sequencing experiments. Nature Biotechnology 2016 feb;34(3):300–302. https://doi.org/10.1038%2Fnbt.3442.
27. Brown CT, Scott C, Crusoe MR, Sheneman L, Rosenthal J, Howe A, khmer-protocols 0.8.4 documentation; 2013. https://figshare.com/articles/khmer_protocols_0_8_3_documentation/878460.
28. Bolger AM, Lohse M, Usadel B. Trimmomatic: a flexible trimmer for Illumina sequence data. Bioinformatics 2014 apr;30(15):2114–2120. https://doi.org/10.1093%2Fbioinformatics%2Fbtu170.
29. Crusoe MR, Alameldin HF, Awad S, Boucher E, Caldwell A, Cartwright R, et al. The khmer software package: enabling efficient nucleotide sequence analysis. F1000Research 2015 sep;https://doi.org/10.12688%2Ff1000research.6924.1.
30. Zhang Q, Awad S, Brown CT. Crossing the streams: a framework for streaming analysis of short DNA sequencing reads 2015 mar;https://doi.org/10.7287%2Fpeerj.preprints.890v1.
31. Keeling PJ, Burki F, Wilcox HM, Allam B, Allen EE, Amaral-Zettler LA, et al. The Marine Microbial Eukaryote Transcriptome Sequencing Project (MMETSP): Illuminating the Functional Diversity of Eukaryotic Life in the Oceans through Transcriptome Sequencing. PLoS Biology 2014 jun;12(6):e1001889. https://doi.org/10.1371%2Fjournal.pbio.1001889.
32. Caron DA, Alexander H, Allen AE, Archibald JM, Armbrust EV, Bachy C, et al. Probing the evolution, ecology and physiology of marine protists using transcriptomics. Nature Reviews Microbiology 2016 nov;15(1):6–20. https://doi.org/10.1038%2Fnrmicro.2016.160.
33. Durkin CA, Koester JA, Bender SJ, Armbrust EV. The evolution of silicon transporters in diatoms. Journal of Phycology 2016 aug;52(5):716–731. https://doi.org/10.1111%2Fjpy.12441.
34. Groussman RD, Parker MS, Armbrust EV. Diversity and Evolutionary History of Iron Metabolism Genes in Diatoms. PLOS ONE 2015 jun;10(6):e0129081. https://doi.org/10.1371%2Fjournal.pone.0129081.
35. Harke MJ, Juhl AR, Haley ST, Alexander H, Dyhrman ST. Conserved Transcriptional Responses to Nutrient Stress in Bloom-Forming Algae. Frontiers in Microbiology 2017 jul;8. https://doi.org/10.3389%2Ffmicb.2017.01279.
36. Koid AE, Liu Z, Terrado R, Jones AC, Caron DA, Heidelberg KB. Comparative Transcriptome Analysis of Four Prymnesiophyte Algae. PLoS ONE 2014 jun;9(6):e97801. https://doi.org/10.1371%2Fjournal.pone.0097801.
37. Alkalaeva E, Mikhailova T. Reassigning stop codons via translation termination: How a few eukaryotes broke the dogma. BioEssays 2016 dec;39(3):1600213. https://doi.org/10.1002%2Fbies.201600213.
38. Heaphy SM, Mariotti M, Gladyshev VN, Atkins JF, Baranov PV. Novel Ciliate Genetic Code Variants Including the Reassignment of All Three Stop Codons to Sense Codons inCondylostoma magnum. Molecular Biology and Evolution 2016 aug;33(11):2885–2889. https://doi.org/10.1093%2Fmolbev%2Fmsw166.
39. Swart EC, Serra V, Petroni G, Nowacki M. Genetic Codes with No Dedicated Stop Codon: Context-Dependent Translation Termination. Cell 2016 jul;166(3):691–702. https://doi.org/10.1016%2Fj.cell.2016.06.020.
40. Lowe EK, Swalla BJ, Brown CT. Evaluating a lightweight transcriptome assembly pipeline on two closely related ascidian species 2014 sep;https://doi.org/10.7287%2Fpeerj.preprints.505v1.
41. Johnson LK, Alexander H, dib-lab/dib-MMETSP: v2; 2018. https://doi.org/10.5281/zenodo.594854.
42. Leinonen R, Sugawara H, and MS. The Sequence Read Archive. Nucleic Acids Research 2010 nov;39(Database):D19–D21. https://doi.org/10.1093%2Fnar%2Fgkq1019.
43. Johnson L, Alexander H, Brown CT. Supplemental Information for MMETSP article: 'Re-assembly, quality evaluation, and annotation of 678 microbial eukaryotic reference transcriptomes' 2018;https://doi.org/10.6084/m9.figshare.7091003.
44. Andrews S, FastQC: A quality control tool for high throughput sequence data.; 2016. http://www.bioinformatics.babraham.ac.uk/projects/fastqc/.
45. Ewels P, Magnusson M, Lundin S, Käller M. MultiQC: summarize analysis results for multiple tools and samples in a single report. Bioinformatics 2016 jun;32(19):3047–3048. https://doi.org/10.1093%2Fbioinformatics%2Fbtw354.
46. MacManes MD. On the optimal trimming of high-throughput mRNA sequence data. Frontiers in Genetics 2014;5. https://doi.org/10.3389%2Ffgene.2014.00013.
47. Brown CT, Howe A, Zhang Q, Pyrkosz AB, Brom TH. A Reference-Free Algorithm for Computational Normalization of Shotgun Sequencing Data 2012 mar;http://arxiv.org/abs/1203.4802.
48. Brown CT, What does Trinity's In Silico normalization do?; 2012. https://doi.org/10.6084/m9.figshare.98198.v1.
49. Scott C, dammit: an open and accessible de novo transcriptome annotator; 2016. www.camillescott.org/dammit.
50. Finn RD, Coggill P, Eberhardt RY, Eddy SR, Mistry J, Mitchell AL, et al. The Pfam protein families database: towards a more sustainable future. Nucleic Acids Research 2015 dec;44(D1):D279–D285. https://doi.org/10.1093%2Fnar%2Fgkv1344.
51. Gardner PP, Daub J, Tate JG, Nawrocki EP, Kolbe DL, Lindgreen S, et al. Rfam: updates to the RNA families database. Nucleic Acids Research 2009 jan;37(Database):D136–D140. https://doi.org/10.1093%2Fnar%2Fgkn766.
52. Zdobnov EM, Tegenfeldt F, Kuznetsov D, Waterhouse RM, Simão FA, Ioannidis P, et al. OrthoDB v9.1: cataloging evolutionary and functional annotations for animal, fungal, plant, archaeal, bacterial and viral orthologs. Nucleic Acids Research 2016 nov;45(D1):D744–D749. https://doi.org/10.1093%2Fnar%2Fgkw1119.
53. Smith-Unna R, Boursnell C, Patro R, Hibberd JM, Kelly S. TransRate: reference-free quality assessment of de novo transcriptome assemblies. Genome Research 2016 jun;26(8):1134–1144. https://doi.org/10.1101%2Fgr.196469.115.
54. Aubry S, Kelly S, Kümpers BMC, Smith-Unna RD, Hibberd JM. Deep Evolutionary Comparison of Gene Expression Identifies Parallel Recruitment of Trans-Factors in Two Independent Origins of C4 Photosynthesis. PLoS Genetics 2014 jun;10(6):e1004365. https://doi.org/10.1371%2Fjournal.pgen.1004365.
55. Simão FA, Waterhouse RM, Ioannidis P, Kriventseva EV, Zdobnov EM. BUSCO: assessing genome assembly and annotation completeness with single-copy orthologs. Bioinformatics 2015 jun;31(19):3210–3212. https://doi.org/10.1093%2Fbioinformatics%2Fbtv351.
56. Junior LCI, Brown CT. Efficient cardinality estimation for k-mers in large DNA sequencing data sets 2016 jun;https://doi.org/10.1101%2F056846.
57. Johnson L, Alexander H, Brown CT, Marine Microbial Eukaryotic Transcriptome Sequencing Project, re-assemblies; 2018. https://doi.org/10.6084/m9.figshare.3840153.
58. Bradnam KR, Fass JN, Alexandrov A, Baranay P, Bech-

ner M, Birol I, et al. Assemblathon 2: evaluating de novo methods of genome assembly in three vertebrate species. GigaScience 2013 jul;2(1). https://doi.org/10.1186%2F2047-217x-2-10.

59. Li B, Fillmore N, Bai Y, Collins M, Thomson JA, Stewart R, et al. Evaluation of de novo transcriptome assemblies from RNA-Seq data. Genome Biology 2014 dec;15(12). https://doi.org/10.1186%2Fs13059-014-0553-5.
60. MacManes MD. The Oyster River Protocol: a multi-assembler and kmer approach for de novo transcriptome assembly. PeerJ 2018 aug;6:e5428. https://doi.org/10.7717%2Fpeerj.5428.
61. Aranda M, Li Y, Liew YJ, Baumgarten S, Simakov O, Wilson MC, et al. Genomes of coral dinoflagellate symbionts highlight evolutionary adaptations conducive to a symbiotic lifestyle. Scientific Reports 2016 dec;6(1). https://doi.org/10.1038%2Fsrep39734.
62. Towns J, Cockerill T, Dahan M, Foster I, Gaither K, Grimshaw A, et al. XSEDE: Accelerating Scientific Discovery. Computing in Science & Engineering 2014 sep;16(5):62–74. https://doi.org/10.1109%2Fmcse.2014.80.
63. Stewart CA, Turner G, Vaughn M, Gaffney NI, Cockerill TM, Foster I, et al. Jetstream. In: Proceedings of the 2015 XSEDE Conference on Scientific Advancements Enabled by Enhanced Cyberinfrastructure - XSEDE '15 ACM Press; 2015. https://doi.org/10.1145%2F2792745.2792774.

Manuscript (LaTeX source) Click here to access/download;Manuscript;main.tex
Click here to view linked References

```
This is pdfTeX, Version 3.14159265-2.6-1.40.19 (TeX Live 2018/W32TeX)
(preloaded format=pdflatex 2018.8.1)  18 SEP 2018 09:15
entering extended mode
 restricted \write18 enabled.
 %&-line parsing enabled.
**main.tex
(./main.tex
LaTeX2e <2018-04-01> patch level 5
(./oup-contemporary.cls
Document Class: oup-contemporary 2017/06/28, v1.1
(c:/TeXLive/2018/texmf-dist/tex/latex/base/article.cls
Document Class: article 2014/09/29 v1.4h Standard LaTeX document class
(c:/TeXLive/2018/texmf-dist/tex/latex/base/size10.clo
File: size10.clo 2014/09/29 v1.4h Standard LaTeX file (size option)
)
\c@part=\count80
\c@section=\count81
\c@subsection=\count82
\c@subsubsection=\count83
\c@paragraph=\count84
\c@subparagraph=\count85
\c@figure=\count86
\c@table=\count87
\abovecaptionskip=\skip41
\belowcaptionskip=\skip42
\bibindent=\dimen102
) (c:/TeXLive/2018/texmf-dist/tex/latex/base/inputenc.sty
Package: inputenc 2018/04/06 v1.3b Input encoding file
\inpenc@prehook=\toks14
\inpenc@posthook=\toks15
) (c:/TeXLive/2018/texmf-dist/tex/latex/base/fontenc.sty
Package: fontenc 2017/04/05 v2.0i Standard LaTeX package
(c:/TeXLive/2018/texmf-dist/tex/latex/base/t1enc.def
File: t1enc.def 2017/04/05 v2.0i Standard LaTeX file
LaTeX Font Info:    Redeclaring font encoding T1 on input line 48.
)) (c:/TeXLive/2018/texmf-dist/tex/generic/oberdiek/ifpdf.sty
Package: ifpdf 2017/03/15 v3.2 Provides the ifpdf switch
) (c:/TeXLive/2018/texmf-dist/tex/latex/microtype/microtype.sty
Package: microtype 2018/01/14 v2.7a Micro-typographical refinements (RS)
(c:/TeXLive/2018/texmf-dist/tex/latex/graphics/keyval.sty
Package: keyval 2014/10/28 v1.15 key=value parser (DPC)
\KV@toks@=\toks16
)
\MT@toks=\toks17
\MT@count=\count88
LaTeX Info: Redefining \textls on input line 793.
\MT@outer@kern=\dimen103
LaTeX Info: Redefining \textmicrotypecontext on input line 1339.
\MT@listname@count=\count89
(c:/TeXLive/2018/texmf-dist/tex/latex/microtype/microtype-pdftex.def
File: microtype-pdftex.def 2018/01/14 v2.7a Definitions specific to
pdftex (RS)

LaTeX Info: Redefining \lsstyle on input line 913.
```

```
LaTeX Info: Redefining \lslig on input line 913.
\MT@outer@space=\skip43
)
Package microtype Info: Loading configuration file microtype.cfg.
(c:/TeXLive/2018/texmf-dist/tex/latex/microtype/microtype.cfg
File: microtype.cfg 2018/01/14 v2.7a microtype main configuration file
(RS)
)) (c:/TeXLive/2018/texmf-dist/tex/latex/euler/euler.sty
Package: euler 1995/03/05 v2.5
Package: `euler' v2.5 <1995/03/05> (FJ and FMi)
LaTeX Font Info:    Redeclaring symbol font `letters' on input line 35.
LaTeX Font Info:    Encoding `OML' has changed to `U' for symbol font
(Font)              `letters' in the math version `normal' on input line
35.
LaTeX Font Info:    Overwriting symbol font `letters' in version `normal'
(Font)                  OML/cmm/m/it --> U/eur/m/n on input line 35.
LaTeX Font Info:    Encoding `OML' has changed to `U' for symbol font
(Font)              `letters' in the math version `bold' on input line
35.
LaTeX Font Info:    Overwriting symbol font `letters' in version `bold'
(Font)                  OML/cmm/b/it --> U/eur/m/n on input line 35.
LaTeX Font Info:    Overwriting symbol font `letters' in version `bold'
(Font)                  U/eur/m/n --> U/eur/b/n on input line 36.
LaTeX Font Info:    Redeclaring math symbol \Gamma on input line 47.
LaTeX Font Info:    Redeclaring math symbol \Delta on input line 48.
LaTeX Font Info:    Redeclaring math symbol \Theta on input line 49.
LaTeX Font Info:    Redeclaring math symbol \Lambda on input line 50.
LaTeX Font Info:    Redeclaring math symbol \Xi on input line 51.
LaTeX Font Info:    Redeclaring math symbol \Pi on input line 52.
LaTeX Font Info:    Redeclaring math symbol \Sigma on input line 53.
LaTeX Font Info:    Redeclaring math symbol \Upsilon on input line 54.
LaTeX Font Info:    Redeclaring math symbol \Phi on input line 55.
LaTeX Font Info:    Redeclaring math symbol \Psi on input line 56.
LaTeX Font Info:    Redeclaring math symbol \Omega on input line 57.
\symEulerFraktur=\mathgroup4
LaTeX Font Info:    Overwriting symbol font `EulerFraktur' in version
`bold'
(Font)                  U/euf/m/n --> U/euf/b/n on input line 63.
LaTeX Info: Redefining \oldstylenums on input line 85.
\symEulerScript=\mathgroup5
LaTeX Font Info:    Overwriting symbol font `EulerScript' in version
`bold'
(Font)                  U/eus/m/n --> U/eus/b/n on input line 93.
LaTeX Font Info:    Redeclaring math symbol \aleph on input line 97.
LaTeX Font Info:    Redeclaring math symbol \Re on input line 98.
LaTeX Font Info:    Redeclaring math symbol \Im on input line 99.
LaTeX Font Info:    Redeclaring math delimiter \vert on input line 101.
LaTeX Font Info:    Redeclaring math delimiter \backslash on input line
103.
LaTeX Font Info:    Redeclaring math symbol \neg on input line 106.
LaTeX Font Info:    Redeclaring math symbol \wedge on input line 108.
LaTeX Font Info:    Redeclaring math symbol \vee on input line 110.
LaTeX Font Info:    Redeclaring math symbol \setminus on input line 112.
LaTeX Font Info:    Redeclaring math symbol \sim on input line 113.
```

```
LaTeX Font Info:    Redeclaring math symbol \mid on input line 114.
LaTeX Font Info:    Redeclaring math delimiter \arrowvert on input line
116.
LaTeX Font Info:    Redeclaring math symbol \mathsection on input line
117.
\symEulerExtension=\mathgroup6
LaTeX Font Info:    Redeclaring math symbol \coprod on input line 125.
LaTeX Font Info:    Redeclaring math symbol \prod on input line 125.
LaTeX Font Info:    Redeclaring math symbol \sum on input line 125.
LaTeX Font Info:    Redeclaring math symbol \intop on input line 130.
LaTeX Font Info:    Redeclaring math symbol \ointop on input line 131.
LaTeX Font Info:    Redeclaring math symbol \braceld on input line 132.
LaTeX Font Info:    Redeclaring math symbol \bracerd on input line 133.
LaTeX Font Info:    Redeclaring math symbol \bracelu on input line 134.
LaTeX Font Info:    Redeclaring math symbol \braceru on input line 135.
LaTeX Font Info:    Redeclaring math symbol \infty on input line 136.
LaTeX Font Info:    Redeclaring math symbol \nearrow on input line 153.
LaTeX Font Info:    Redeclaring math symbol \searrow on input line 154.
LaTeX Font Info:    Redeclaring math symbol \nwarrow on input line 155.
LaTeX Font Info:    Redeclaring math symbol \swarrow on input line 156.
LaTeX Font Info:    Redeclaring math symbol \Leftrightarrow on input line
157.
LaTeX Font Info:    Redeclaring math symbol \Leftarrow on input line 158.
LaTeX Font Info:    Redeclaring math symbol \Rightarrow on input line
159.
LaTeX Font Info:    Redeclaring math symbol \leftrightarrow on input line
160.
LaTeX Font Info:    Redeclaring math symbol \leftarrow on input line 161.
LaTeX Font Info:    Redeclaring math symbol \rightarrow on input line
163.
LaTeX Font Info:    Redeclaring math delimiter \uparrow on input line
166.
LaTeX Font Info:    Redeclaring math delimiter \downarrow on input line
168.
LaTeX Font Info:    Redeclaring math delimiter \updownarrow on input line
170.
LaTeX Font Info:    Redeclaring math delimiter \Uparrow on input line
172.
LaTeX Font Info:    Redeclaring math delimiter \Downarrow on input line
174.
LaTeX Font Info:    Redeclaring math delimiter \Updownarrow on input line
176.
LaTeX Font Info:    Redeclaring math symbol \leftharpoonup on input line
177.
LaTeX Font Info:    Redeclaring math symbol \leftharpoondown on input
line 178.

LaTeX Font Info:    Redeclaring math symbol \rightharpoonup on input line
179.
LaTeX Font Info:    Redeclaring math symbol \rightharpoondown on input
line 180
.
LaTeX Font Info:    Redeclaring math delimiter \lbrace on input line 182.
LaTeX Font Info:    Redeclaring math delimiter \rbrace on input line 184.
```

```
\symcmmigroup=\mathgroup7
LaTeX Font Info:    Overwriting symbol font `cmmigroup' in version `bold'
(Font)                  OML/cmm/m/it --> OML/cmm/b/it on input line 200.
LaTeX Font Info:    Redeclaring math accent \vec on input line 201.
LaTeX Font Info:    Redeclaring math symbol \triangleleft on input line
202.
LaTeX Font Info:    Redeclaring math symbol \triangleright on input line
203.
LaTeX Font Info:    Redeclaring math symbol \star on input line 204.
LaTeX Font Info:    Redeclaring math symbol \lhook on input line 205.
LaTeX Font Info:    Redeclaring math symbol \rhook on input line 206.
LaTeX Font Info:    Redeclaring math symbol \flat on input line 207.
LaTeX Font Info:    Redeclaring math symbol \natural on input line 208.
LaTeX Font Info:    Redeclaring math symbol \sharp on input line 209.
LaTeX Font Info:    Redeclaring math symbol \smile on input line 210.
LaTeX Font Info:    Redeclaring math symbol \frown on input line 211.
LaTeX Font Info:    Redeclaring math accent \grave on input line 245.
LaTeX Font Info:    Redeclaring math accent \acute on input line 246.
LaTeX Font Info:    Redeclaring math accent \tilde on input line 247.
LaTeX Font Info:    Redeclaring math accent \ddot on input line 248.
LaTeX Font Info:    Redeclaring math accent \check on input line 249.
LaTeX Font Info:    Redeclaring math accent \breve on input line 250.
LaTeX Font Info:    Redeclaring math accent \bar on input line 251.
LaTeX Font Info:    Redeclaring math accent \dot on input line 252.
LaTeX Font Info:    Redeclaring math accent \hat on input line 254.
) (c:/TeXLive/2018/texmf-dist/tex/latex/merriweather/merriweather.sty
Package: merriweather 2014/01/22 (Bob Tennent) Supports
Merriweather(Sans) font
s for all LaTeX engines.
(c:/TeXLive/2018/texmf-dist/tex/generic/ifxetex/ifxetex.sty
Package: ifxetex 2010/09/12 v0.6 Provides ifxetex conditional
) (c:/TeXLive/2018/texmf-dist/tex/generic/oberdiek/ifluatex.sty
Package: ifluatex 2016/05/16 v1.4 Provides the ifluatex switch (HO)
Package ifluatex Info: LuaTeX not detected.
) (c:/TeXLive/2018/texmf-dist/tex/latex/base/textcomp.sty
Package: textcomp 2017/04/05 v2.0i Standard LaTeX package
Package textcomp Info: Sub-encoding information:
(textcomp)               5 = only ISO-Adobe without \textcurrency
(textcomp)               4 = 5 + \texteuro
(textcomp)               3 = 4 + \textohm
(textcomp)               2 = 3 + \textestimated + \textcurrency
(textcomp)               1 = TS1 - \textcircled - \t
(textcomp)               0 = TS1 (full)
(textcomp)             Font families with sub-encoding setting implement
(textcomp)             only a restricted character set as indicated.
(textcomp)             Family '?' is the default used for unknown fonts.
(textcomp)             See the documentation for details.
Package textcomp Info: Setting ? sub-encoding to TS1/1 on input line 79.
(c:/TeXLive/2018/texmf-dist/tex/latex/base/ts1enc.def
File: ts1enc.def 2001/06/05 v3.0e (jk/car/fm) Standard LaTeX file
Now handling font encoding TS1 ...
... processing UTF-8 mapping file for font encoding TS1
(c:/TeXLive/2018/texmf-dist/tex/latex/base/ts1enc.dfu
File: ts1enc.dfu 2018/04/05 v1.2c UTF-8 support for inputenc
```

```
defining Unicode char U+00A2 (decimal 162)
defining Unicode char U+00A3 (decimal 163)
defining Unicode char U+00A4 (decimal 164)
defining Unicode char U+00A5 (decimal 165)
defining Unicode char U+00A6 (decimal 166)
defining Unicode char U+00A7 (decimal 167)
defining Unicode char U+00A8 (decimal 168)
defining Unicode char U+00A9 (decimal 169)
defining Unicode char U+00AA (decimal 170)
defining Unicode char U+00AC (decimal 172)
defining Unicode char U+00AE (decimal 174)
defining Unicode char U+00AF (decimal 175)
defining Unicode char U+00B0 (decimal 176)
defining Unicode char U+00B1 (decimal 177)
defining Unicode char U+00B2 (decimal 178)
defining Unicode char U+00B3 (decimal 179)
defining Unicode char U+00B4 (decimal 180)
defining Unicode char U+00B5 (decimal 181)
defining Unicode char U+00B6 (decimal 182)
defining Unicode char U+00B7 (decimal 183)
defining Unicode char U+00B9 (decimal 185)
defining Unicode char U+00BA (decimal 186)
defining Unicode char U+00BC (decimal 188)
defining Unicode char U+00BD (decimal 189)
defining Unicode char U+00BE (decimal 190)
defining Unicode char U+00D7 (decimal 215)
defining Unicode char U+00F7 (decimal 247)
defining Unicode char U+0192 (decimal 402)
defining Unicode char U+02C7 (decimal 711)
defining Unicode char U+02D8 (decimal 728)
defining Unicode char U+02DD (decimal 733)
defining Unicode char U+0E3F (decimal 3647)
defining Unicode char U+2016 (decimal 8214)
defining Unicode char U+2020 (decimal 8224)
defining Unicode char U+2021 (decimal 8225)
defining Unicode char U+2022 (decimal 8226)
defining Unicode char U+2030 (decimal 8240)
defining Unicode char U+2031 (decimal 8241)
defining Unicode char U+203B (decimal 8251)
defining Unicode char U+203D (decimal 8253)
defining Unicode char U+2044 (decimal 8260)
defining Unicode char U+204E (decimal 8270)
defining Unicode char U+2052 (decimal 8274)
defining Unicode char U+20A1 (decimal 8353)
defining Unicode char U+20A4 (decimal 8356)
defining Unicode char U+20A6 (decimal 8358)
defining Unicode char U+20A9 (decimal 8361)
defining Unicode char U+20AB (decimal 8363)
defining Unicode char U+20AC (decimal 8364)
defining Unicode char U+20B1 (decimal 8369)
defining Unicode char U+2103 (decimal 8451)
defining Unicode char U+2116 (decimal 8470)
defining Unicode char U+2117 (decimal 8471)
defining Unicode char U+211E (decimal 8478)
```

```
   defining Unicode char U+2120 (decimal 8480)
   defining Unicode char U+2122 (decimal 8482)
   defining Unicode char U+2126 (decimal 8486)
   defining Unicode char U+2127 (decimal 8487)
   defining Unicode char U+212E (decimal 8494)
   defining Unicode char U+2190 (decimal 8592)
   defining Unicode char U+2191 (decimal 8593)
   defining Unicode char U+2192 (decimal 8594)
   defining Unicode char U+2193 (decimal 8595)
   defining Unicode char U+2329 (decimal 9001)
   defining Unicode char U+232A (decimal 9002)
   defining Unicode char U+2422 (decimal 9250)
   defining Unicode char U+25E6 (decimal 9702)
   defining Unicode char U+25EF (decimal 9711)
   defining Unicode char U+266A (decimal 9834)
   defining Unicode char U+FEFF (decimal 65279)
))
LaTeX Info: Redefining \oldstylenums on input line 334.
Package textcomp Info: Setting cmr sub-encoding to TS1/0 on input line
349.
Package textcomp Info: Setting cmss sub-encoding to TS1/0 on input line
350.
Package textcomp Info: Setting cmtt sub-encoding to TS1/0 on input line
351.
Package textcomp Info: Setting cmvtt sub-encoding to TS1/0 on input line
352.
Package textcomp Info: Setting cmbr sub-encoding to TS1/0 on input line
353.
Package textcomp Info: Setting cmtl sub-encoding to TS1/0 on input line
354.
Package textcomp Info: Setting ccr sub-encoding to TS1/0 on input line
355.
Package textcomp Info: Setting ptm sub-encoding to TS1/4 on input line
356.
Package textcomp Info: Setting pcr sub-encoding to TS1/4 on input line
357.
Package textcomp Info: Setting phv sub-encoding to TS1/4 on input line
358.
Package textcomp Info: Setting ppl sub-encoding to TS1/3 on input line
359.
Package textcomp Info: Setting pag sub-encoding to TS1/4 on input line
360.
Package textcomp Info: Setting pbk sub-encoding to TS1/4 on input line
361.
Package textcomp Info: Setting pnc sub-encoding to TS1/4 on input line
362.
Package textcomp Info: Setting pzc sub-encoding to TS1/4 on input line
363.
Package textcomp Info: Setting bch sub-encoding to TS1/4 on input line
364.
Package textcomp Info: Setting put sub-encoding to TS1/5 on input line
365.
Package textcomp Info: Setting uag sub-encoding to TS1/5 on input line
366.
```

```
Package textcomp Info: Setting ugq sub-encoding to TS1/5 on input line 367.
Package textcomp Info: Setting ul8 sub-encoding to TS1/4 on input line 368.
Package textcomp Info: Setting ul9 sub-encoding to TS1/4 on input line 369.
Package textcomp Info: Setting augie sub-encoding to TS1/5 on input line 370.
Package textcomp Info: Setting dayrom sub-encoding to TS1/3 on input line 371.
Package textcomp Info: Setting dayroms sub-encoding to TS1/3 on input line 372.

Package textcomp Info: Setting pxr sub-encoding to TS1/0 on input line 373.
Package textcomp Info: Setting pxss sub-encoding to TS1/0 on input line 374.
Package textcomp Info: Setting pxtt sub-encoding to TS1/0 on input line 375.
Package textcomp Info: Setting txr sub-encoding to TS1/0 on input line 376.
Package textcomp Info: Setting txss sub-encoding to TS1/0 on input line 377.
Package textcomp Info: Setting txtt sub-encoding to TS1/0 on input line 378.
Package textcomp Info: Setting lmr sub-encoding to TS1/0 on input line 379.
Package textcomp Info: Setting lmdh sub-encoding to TS1/0 on input line 380.
Package textcomp Info: Setting lmss sub-encoding to TS1/0 on input line 381.
Package textcomp Info: Setting lmssq sub-encoding to TS1/0 on input line 382.
Package textcomp Info: Setting lmvtt sub-encoding to TS1/0 on input line 383.
Package textcomp Info: Setting lmtt sub-encoding to TS1/0 on input line 384.
Package textcomp Info: Setting qhv sub-encoding to TS1/0 on input line 385.
Package textcomp Info: Setting qag sub-encoding to TS1/0 on input line 386.
Package textcomp Info: Setting qbk sub-encoding to TS1/0 on input line 387.
Package textcomp Info: Setting qcr sub-encoding to TS1/0 on input line 388.
Package textcomp Info: Setting qcs sub-encoding to TS1/0 on input line 389.
Package textcomp Info: Setting qpl sub-encoding to TS1/0 on input line 390.
Package textcomp Info: Setting qtm sub-encoding to TS1/0 on input line 391.
Package textcomp Info: Setting qzc sub-encoding to TS1/0 on input line 392.
```

```
Package textcomp Info: Setting qhvc sub-encoding to TS1/0 on input line
393.
Package textcomp Info: Setting futs sub-encoding to TS1/4 on input line
394.
Package textcomp Info: Setting futx sub-encoding to TS1/4 on input line
395.
Package textcomp Info: Setting futj sub-encoding to TS1/4 on input line
396.
Package textcomp Info: Setting hlh sub-encoding to TS1/3 on input line
397.
Package textcomp Info: Setting hls sub-encoding to TS1/3 on input line
398.
Package textcomp Info: Setting hlst sub-encoding to TS1/3 on input line
399.
Package textcomp Info: Setting hlct sub-encoding to TS1/5 on input line
400.
Package textcomp Info: Setting hlx sub-encoding to TS1/5 on input line
401.
Package textcomp Info: Setting hlce sub-encoding to TS1/5 on input line
402.
Package textcomp Info: Setting hlcn sub-encoding to TS1/5 on input line
403.
Package textcomp Info: Setting hlcw sub-encoding to TS1/5 on input line
404.
Package textcomp Info: Setting hlcf sub-encoding to TS1/5 on input line
405.
Package textcomp Info: Setting pplx sub-encoding to TS1/3 on input line
406.
Package textcomp Info: Setting pplj sub-encoding to TS1/3 on input line
407.
Package textcomp Info: Setting ptmx sub-encoding to TS1/4 on input line
408.
Package textcomp Info: Setting ptmj sub-encoding to TS1/4 on input line
409.
) (c:/TeXLive/2018/texmf-dist/tex/latex/xkeyval/xkeyval.sty
Package: xkeyval 2014/12/03 v2.7a package option processing (HA)
(c:/TeXLive/2018/texmf-dist/tex/generic/xkeyval/xkeyval.tex
(c:/TeXLive/2018/te
xmf-dist/tex/generic/xkeyval/xkvutils.tex
\XKV@toks=\toks18
\XKV@tempa@toks=\toks19
)
\XKV@depth=\count90
File: xkeyval.tex 2014/12/03 v2.7a key=value parser (HA)
)) (c:/TeXLive/2018/texmf-dist/tex/latex/base/fontenc.sty
Package: fontenc 2017/04/05 v2.0i Standard LaTeX package
) (c:/TeXLive/2018/texmf-dist/tex/latex/fontaxes/fontaxes.sty
Package: fontaxes 2014/03/23 v1.0d Font selection axes
LaTeX Info: Redefining \upshape on input line 29.
LaTeX Info: Redefining \itshape on input line 31.
LaTeX Info: Redefining \slshape on input line 33.
LaTeX Info: Redefining \scshape on input line 37.
) (c:/TeXLive/2018/texmf-dist/tex/latex/mweights/mweights.sty
```

```
Package: mweights 2017/03/30 (Bob Tennent) Support package for multiple-
weight
font packages.
LaTeX Info: Redefining \bfseries on input line 22.
LaTeX Info: Redefining \mdseries on input line 30.
LaTeX Info: Redefining \rmfamily on input line 38.
LaTeX Info: Redefining \sffamily on input line 66.
LaTeX Info: Redefining \ttfamily on input line 94.
))

! LaTeX Error: File `mathastext.sty' not found.

Type X to quit or <RETURN> to proceed,
or enter new name. (Default extension: sty)

Enter file name:
! Emergency stop.
<read *>

l.47 \RequirePackage
                    {relsize}^^M
*** (cannot \read from terminal in nonstop modes)


Here is how much of TeX's memory you used:
 2829 strings out of 492642
 45105 string characters out of 6133163
 131660 words of memory out of 5000000
 6734 multiletter control sequences out of 15000+600000
 4403 words of font info for 15 fonts, out of 8000000 for 9000
 1141 hyphenation exceptions out of 8191
 38i,0n,40p,255b,89s stack positions out of
5000i,500n,10000p,200000b,80000s
!  ==> Fatal error occurred, no output PDF file produced!
```

Figure 1

Click here to access/download;Figure;MMETSP_pipeline_paper.png

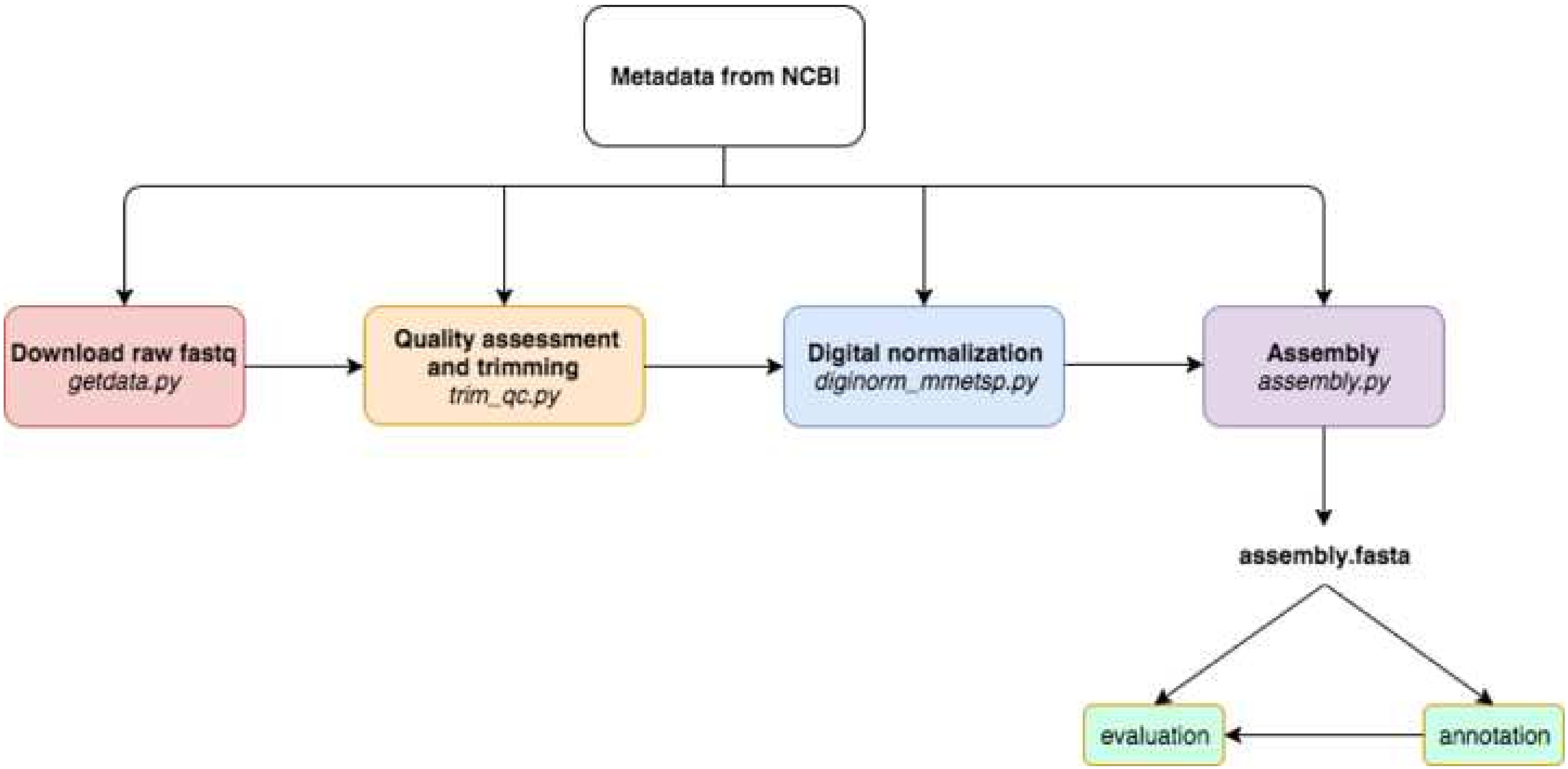

Click here to access/download;Figure;n_contigs.png

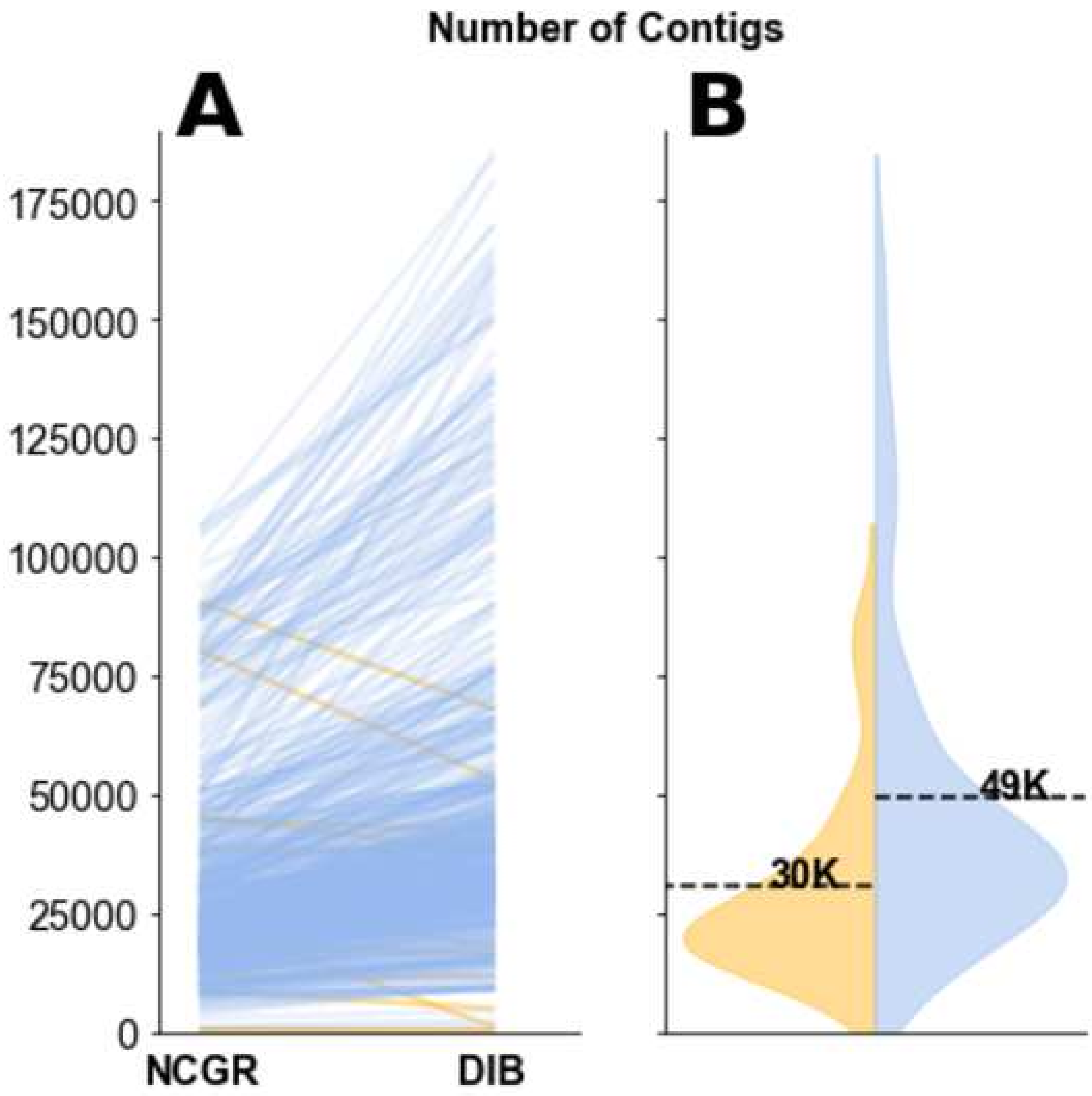

Click here to access/download;Figure;hist_transrate_diff.png

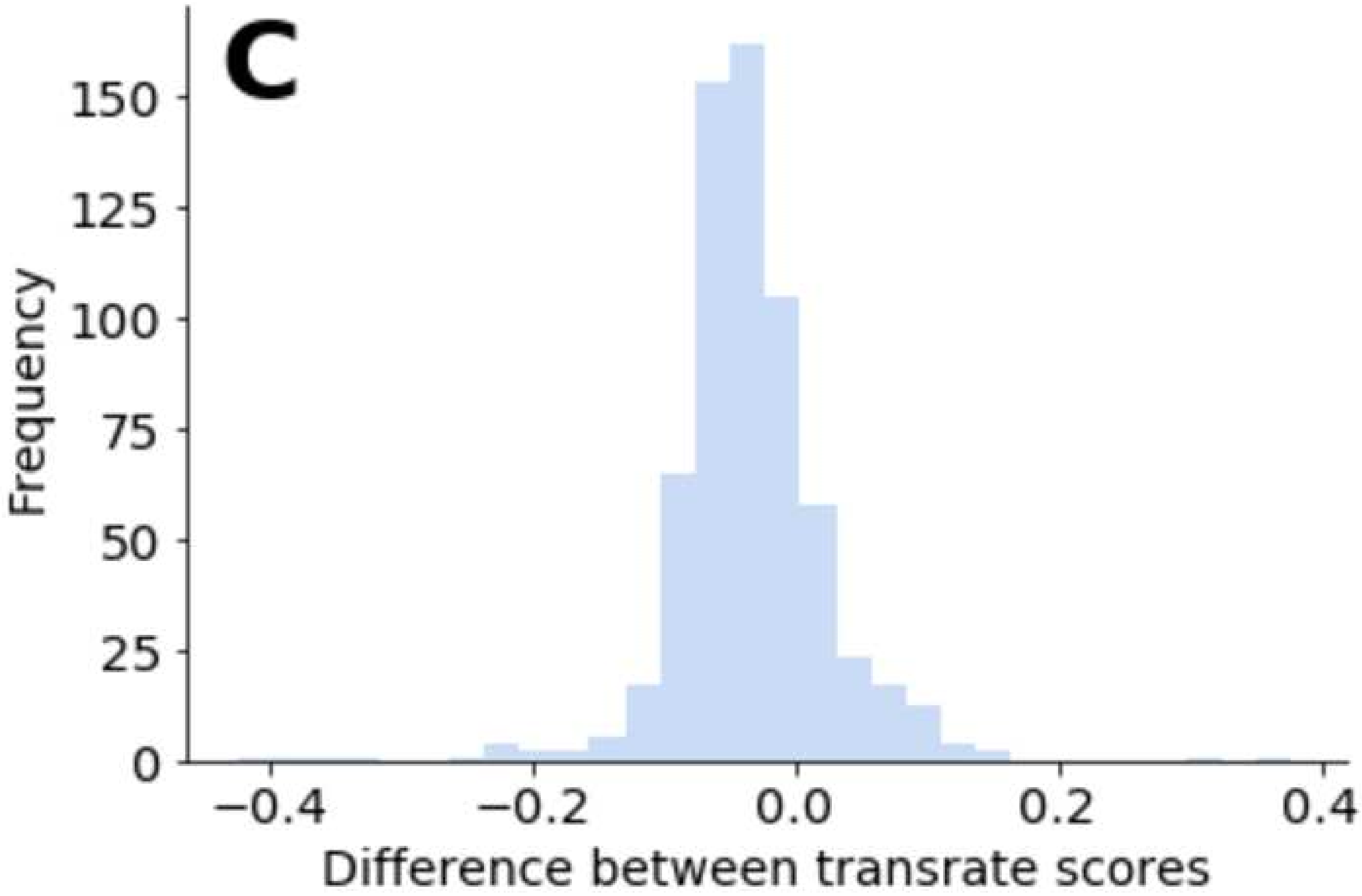

 Click here to access/download;Figure;CRBB-blast.png

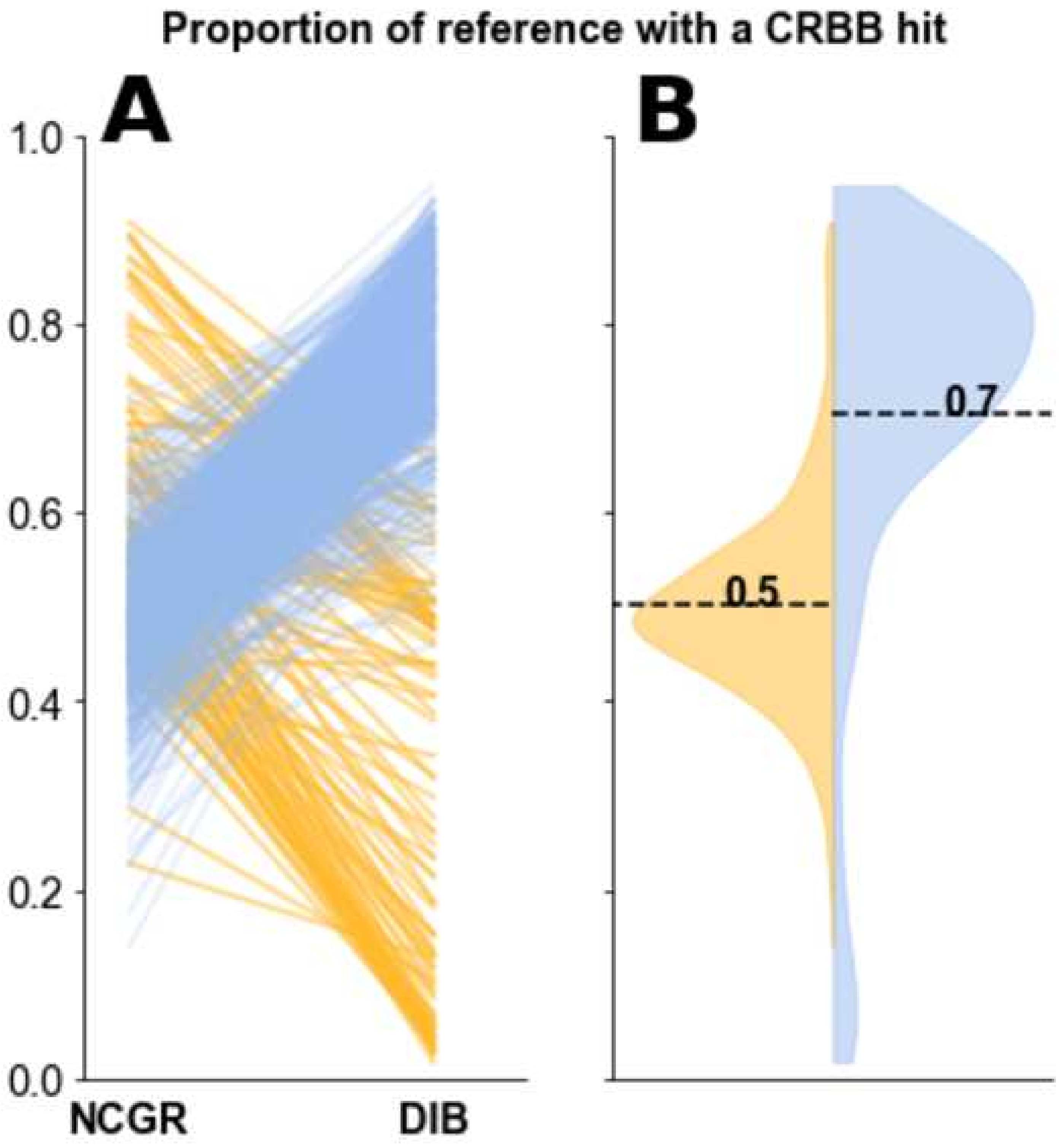

Figure 4

Click here to access/download;Figure;Figure4_unique_kmers.png

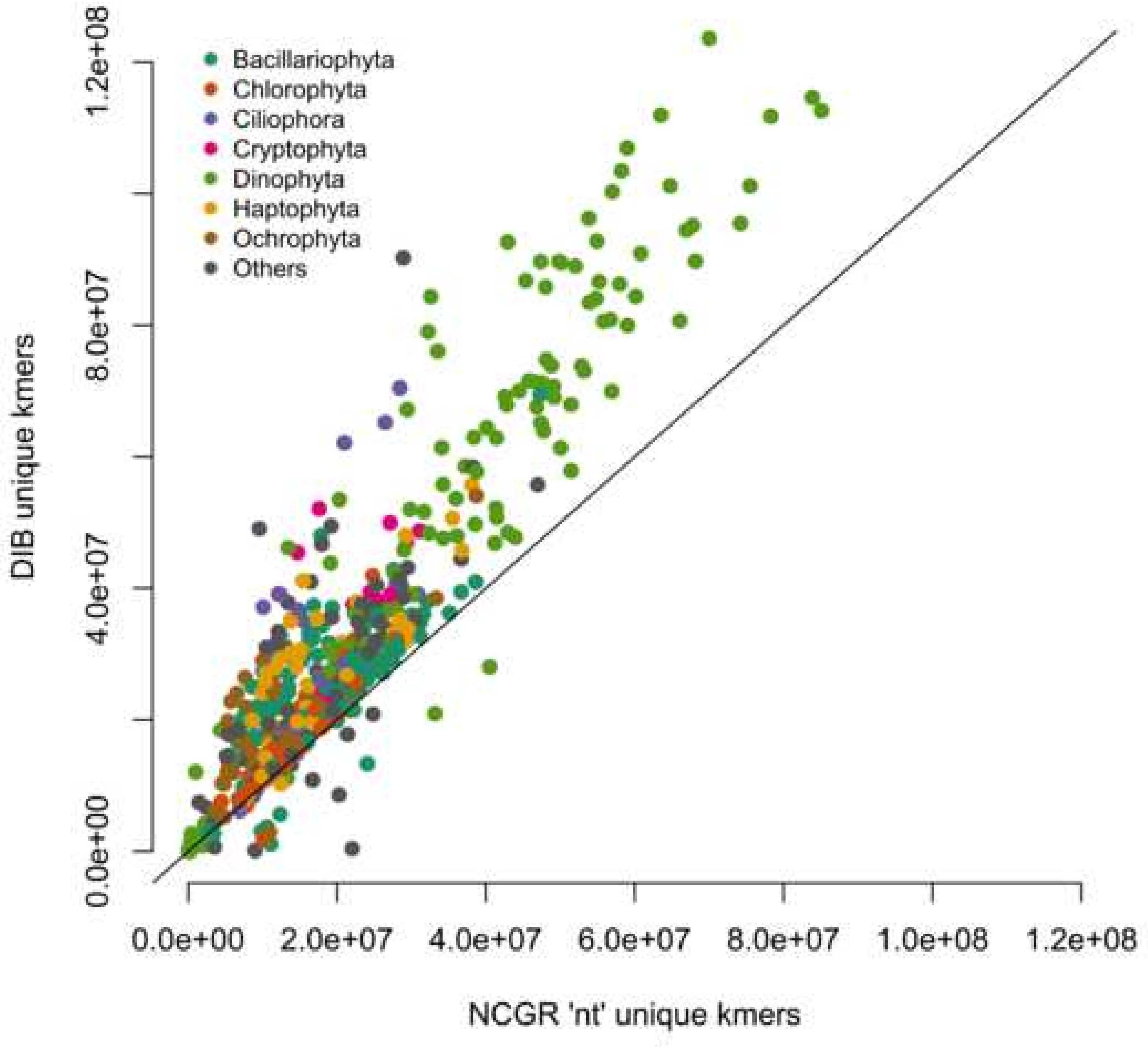

Click here to access/download;Figure;mean_ORF.png

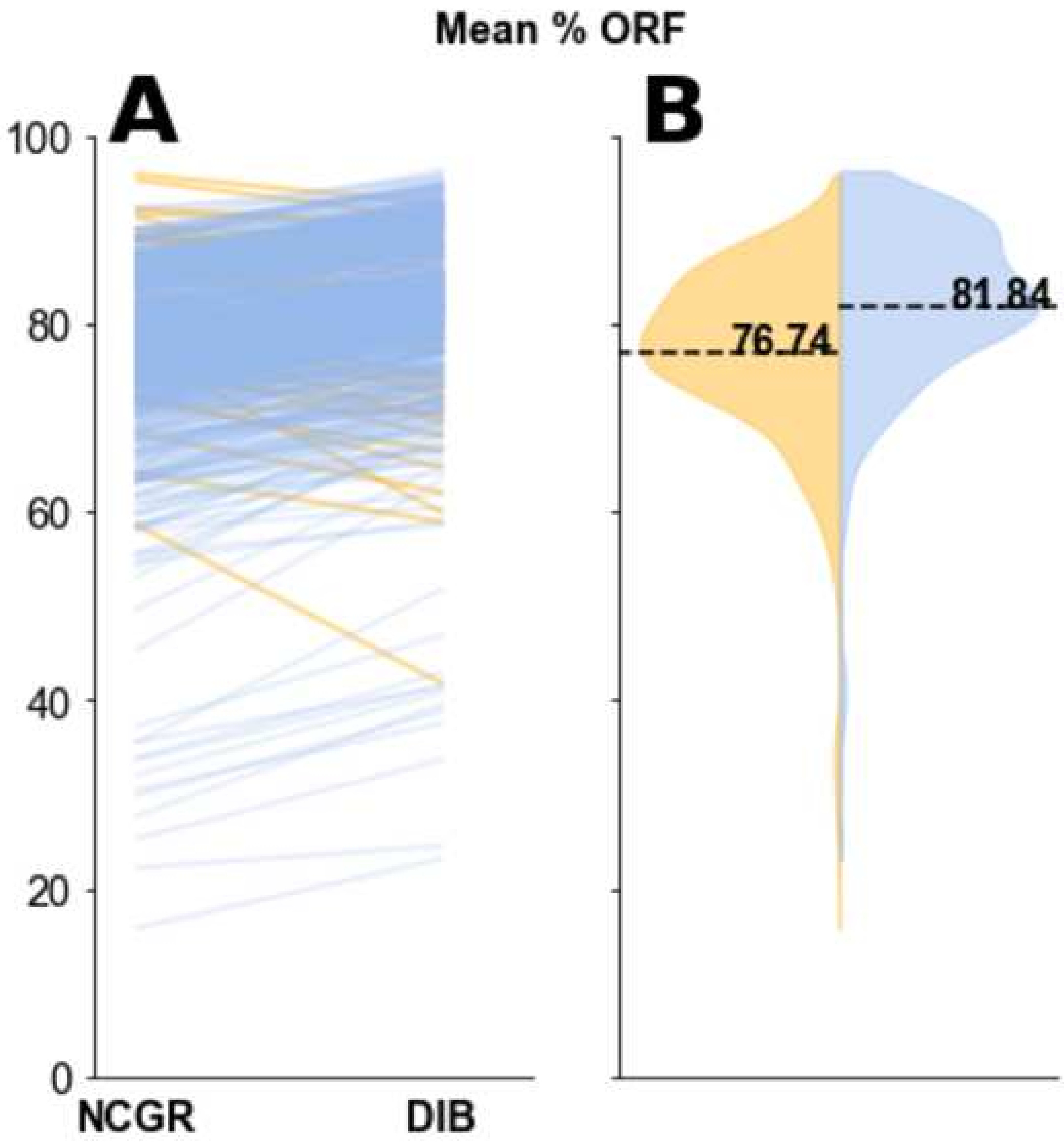

Figure 5cd

Click here to access/download;Figure;mean_euk_BUSCO_v3.png

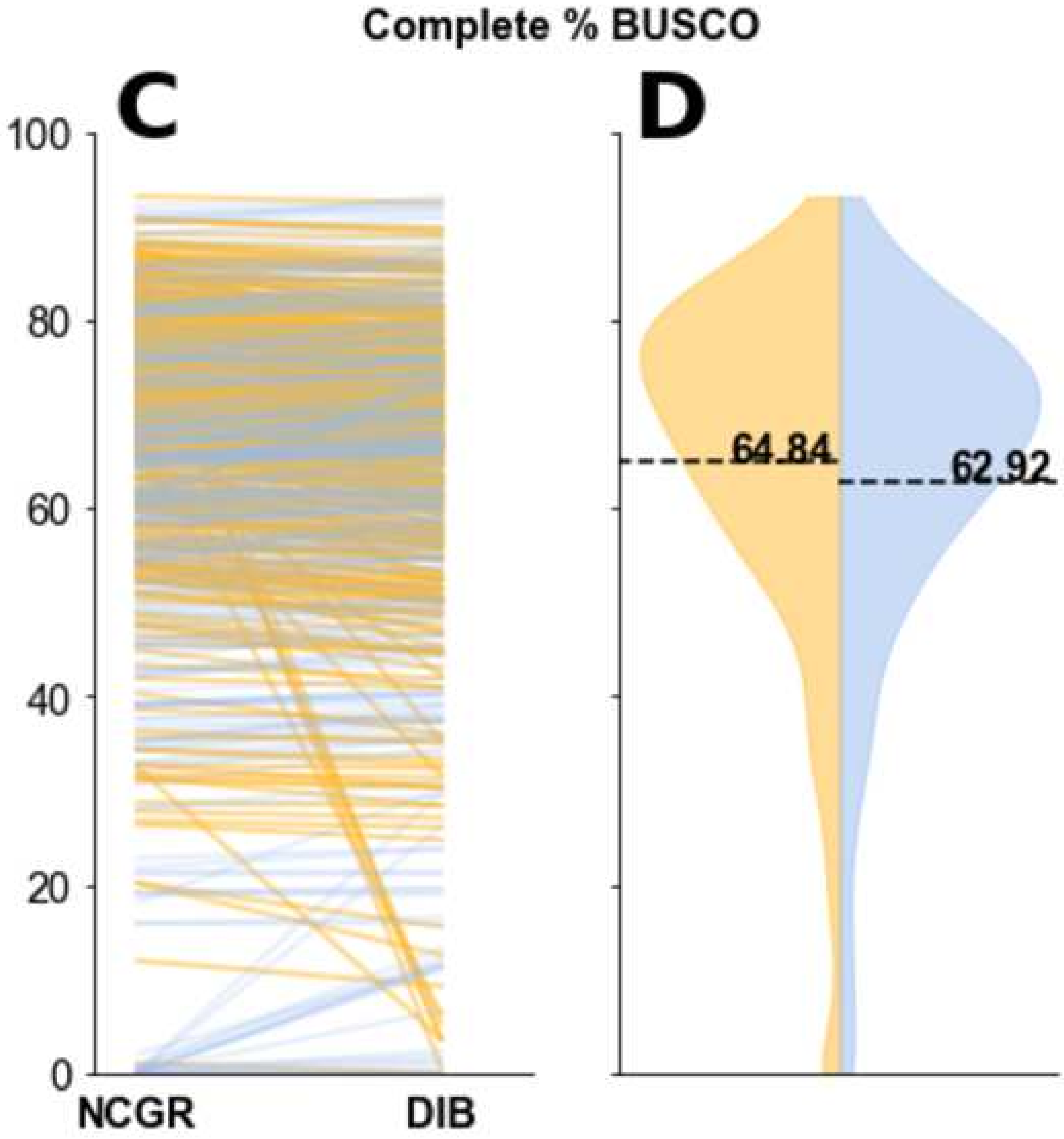

Figure 6

Click here to access/download;Figure;false_crbb.png

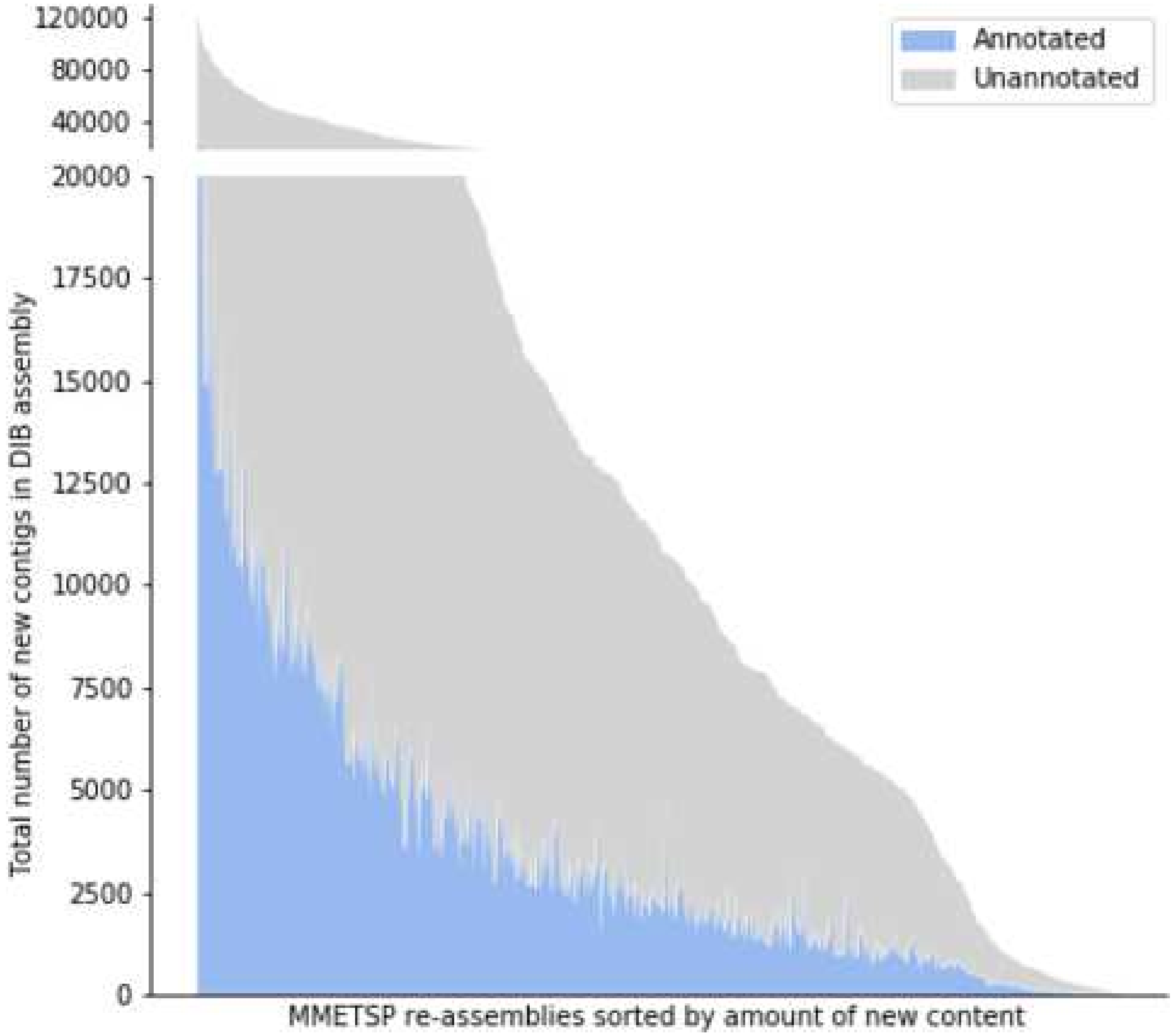

Figure 7

Click here to access/download;Figure;Figure7_unique_gene_names.png

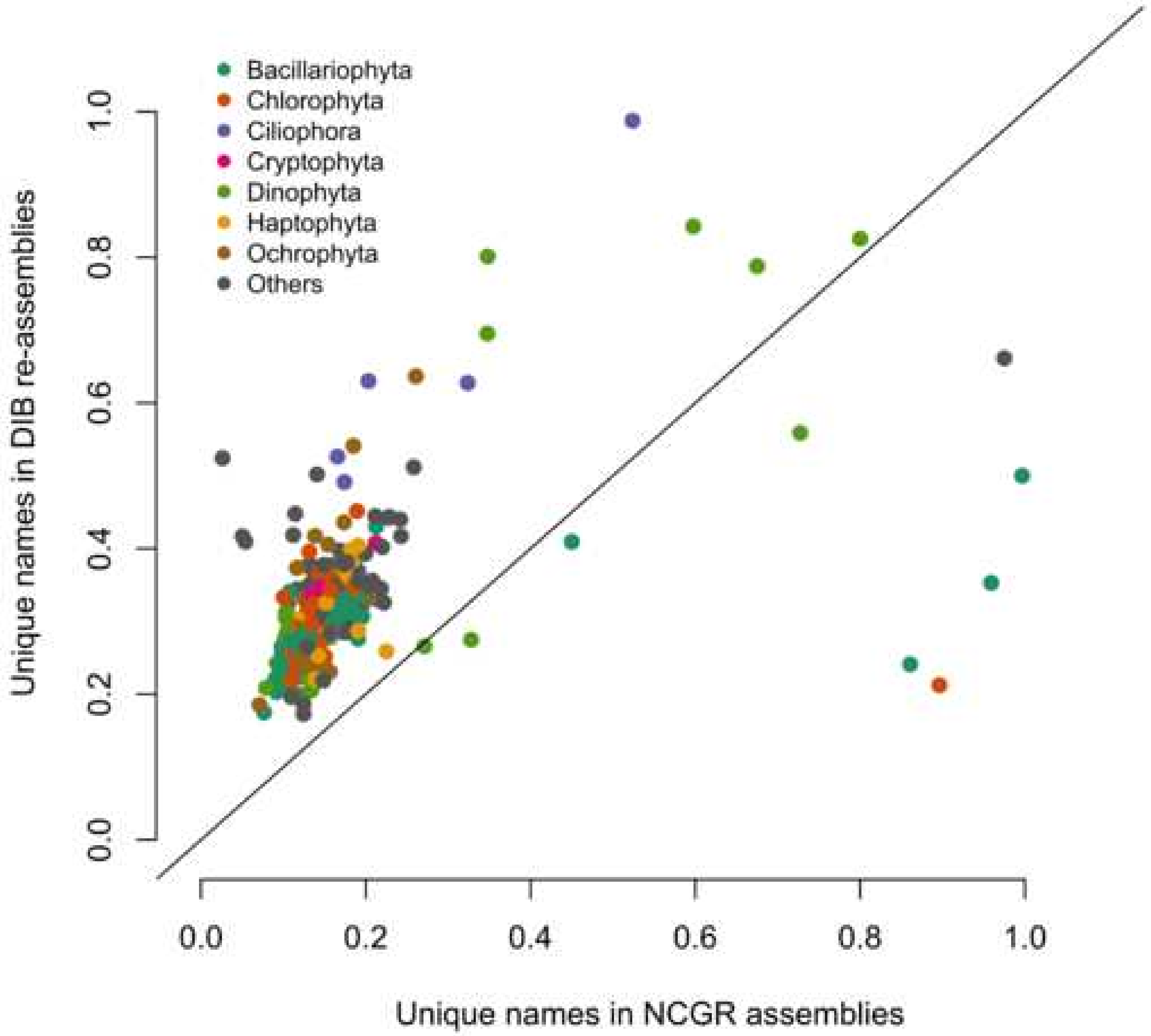

Figure 8

Click here to access/download;Figure;Figure8_all_small.png

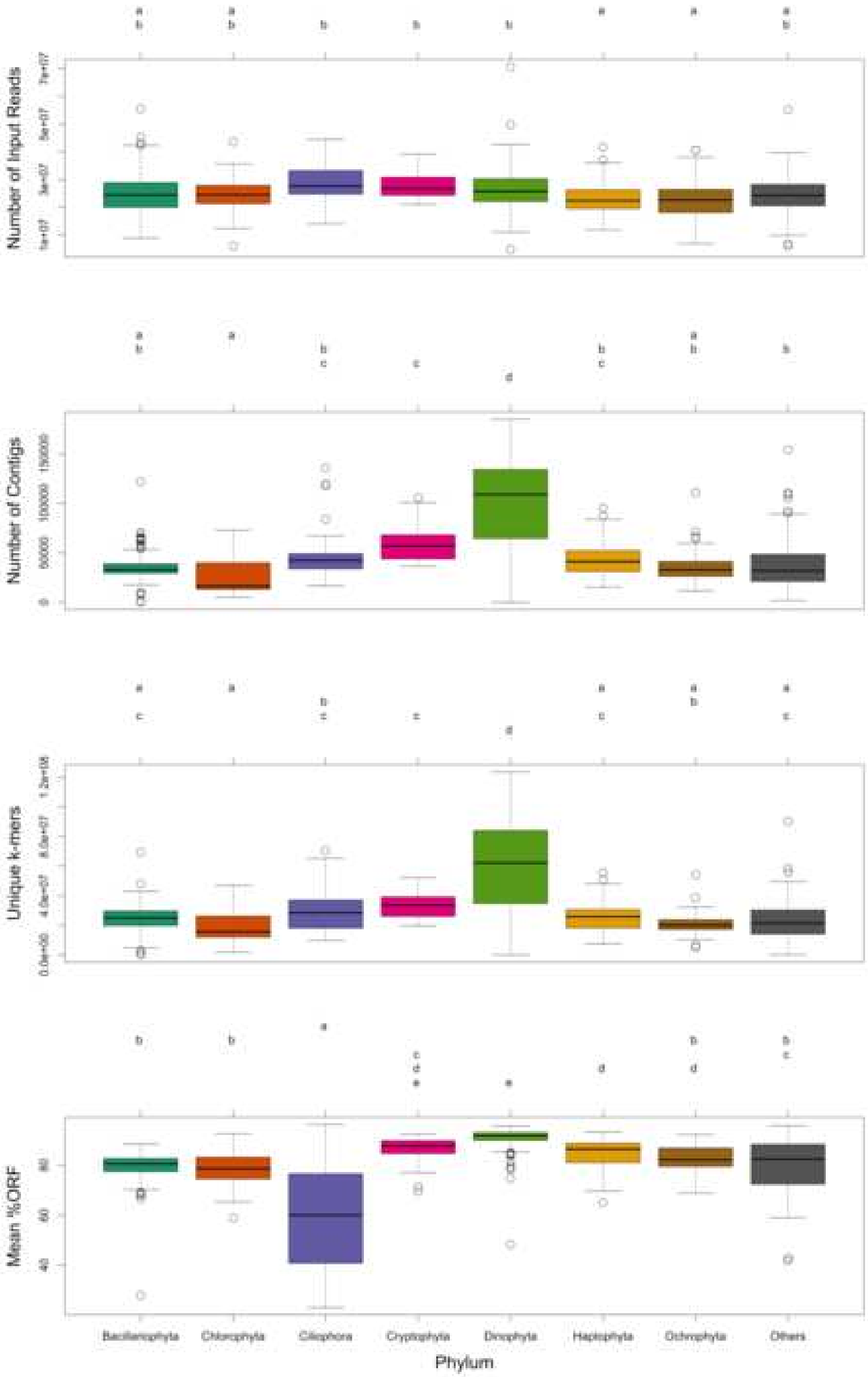

Supplement: GIGA-D-18-00191_Revision-1.pdf [file giy158_giga-d-18-00191_revision-1.pdf]
