# Supplementary material for: Re-assembly, quality evaluation, and annotation of 678 microbial eukaryotic reference transcriptomes
Source: Gigascience. 2018 Dec 13;8(4):giy158. doi: 10.1093/gigascience/giy158 (PMC6481552; doi:10.1093/gigascience/giy158)
Supplement: Response-to-Reviewer-Comments_Original-Submission.pdf [file giy158_response-to-reviewer-comments_original-submission.pdf]

Author's Response To Reviewer Comments

Close

Reviewer #1: The manuscript submitted by Lisa Johnson and colleagues is a well written and comprehensive work aimed at reanalyzing a fairly enormous dataset. I feel that the work is important for the following two main reasons:
1. Assembly methods have improved substantially since the original datasets were analyzed, and as the authors point out - these new analyses recover new transcripts that might be useful to the original researchers and to the broader community.
2. Applying standardized and reproducible methods - at scale - is challenging, and the authors provide an example for how this could be done. I can imagine others using these ideas (or the actual code) to assemble other datasets in a similar fashion.
In terms of the manuscript itself, it is sound, with just a few areas where improvements will make for a more readable paper. Interspersed with this, I have a few more pedantic suggestions that the author should feel free to ignore if deemed unhelpful.

We thank the reviewer for their helpful comments and insights into our work. Below we note alterations and some more detailed clarifications to address the comments.

Line 91: replace 'higher' with 'more favorable' or even 'better'

Changed to: "Here, we show that our re-assemblies had better evaluation metrics and contained most of the NCGR contigs as well as adding new content."

L102: The link to the code does not seem to be active. I would have loved to review it.

The link is here: https://doi.org/10.5281/zenodo.594854 and active in the current version of the GigaScience formatted manuscript.

L111: You are using 50bp reads. Do you think your conclusions would have been any different had longer reads (100-150bp) been available? More novice readers might wonder if these methods are just as applicable to them with longer reads as they are to you. I'm sure the answer is yes - your new assemblies might have been even better had you had longer reads.

Thank you for bringing this point to our attention. This is an important point to mention in the discussion. We added to the end of the second section of the discussion as an extension of subheading: "Reassembly with new tools can yield new results." (L327):

```
We predict that assembly metrics could have been further improved with longer read lengths of the original data since MMETSP data had only 50 bp read lengths, although this would have presented Keeling et al. [31] a more expensive data collection endeavor. A study by Chang et al. [[25](https://www.ncbi.nlm.nih.gov/pmc/articles/PMC3988101/)] reported a consistent
```

increase in the percentage of full-length transcript reconstruction and a decrease in the false positive rate moving from 50 to 100 bp read lengths with the Trinity assembler. However, regardless of length, the conclusions we draw here would likely remain the same that assembling data with new tools can yield new results.
```

L180-182: If I didn't know khmer already, I might struggle with the HyperLogLog estimator. Maybe just a sentence or 3 more might be useful to explain what this is and why it is used.

L182 added: "We used the HLL function to digest each assembly and count the number of distinct fixed-length substrings of DNA (k-mers)."

L245ish: I keep wondering about your BUSCO scores, and the fact tat they are lower on average in your new assemblies compared to the older ones. Why is this? How do you reconcile this with the more general statement you are trying to make about 'more genes being recovered' in the new assembly. I see that BUSCO is just one of the available metrics to assess this, but it's a little strange I think, given that I'm convinced that these assemblies are actually better.

On average, BUSCO scores were lower in DIB vs. NCGR assemblies. However, the degree of the differences was not as dramatic (NCGR mean 64.8 vs. DIB 62.9) compared to differences between the number of contigs (NCGR length mean 30K vs. DIB 49K) and the CRBB differences (NCGR mean proportion 0.5 vs. DIB 0.7).

After re-checking the BUSCO v3 scores against the eukaryota and protista databases, we changed the mean, sd, and k-s test numbers listed in the text. The original numbers were missing a few assemblies and were from BUSCO version 2. The % complete BUSCO are still significantly higher in NCGR vs. DIB.

We edited this sentence and emphasized the less dramatic differences in % ORF and BUSCO scores relative to contig number and CRBB differences:

""
Therefore, although the number of contigs and amount of CRBB content were dramatically increased in the DIB re-assemblies compared to the NCGR assemblies, the differences in ORF content and BUSCO matches compared to the eukaryotic (Figure 5 C,D) and protistan (Supplemental Figure 3) databases - while they were significantly different - were less dramatic. This suggests that content was not lost by gaining extra contigs. The extra content contained similar proportions of ORFs and BUSCO annotations. Therefore, the re-assemblies may contribute more biologically meaningful information.

""

Looking at the eight samples where NCGR had >30% higher complete BUSCO evaluation score (MMETSP0121, MMETSP0932, MMETSP0045, MMETSP0169, MMETSP0232, MMETSP0439, MMETSP0329, MMETSP0717), we see different reasons for missing Complete BUSCOs.
```

```
SampleName Complete_BUSCO_perc_NCGR Complete_BUSCO_perc_DIB
18 MMETSP0121 65.016502 31.683168
134 MMETSP0932 85.148515 3.630363
232 MMETSP0045 73.927393 35.313531
282 MMETSP0169 68.646865 6.270627
451 MMETSP0232 82.508251 0.660066
475 MMETSP0439 80.858086 3.630363
654 MMETSP0329 80.198020 5.280528
661 MMETSP0717 61.716172 17.821782
```
(BUSCO output is not straight-forward to parse.)

https://github.com/ljcohen/MMETSP/blob/master/assembly_evaluation_data/busco_eval/busco_eval.ipynb

For reasons that we don't understand, in some cases a particular orthogroup in the BUSCO db does not produce output in `hmmer_output/`. Sometimes it does. For example, the Trininty-based pipeline only produced 342 contigs for sample MMETSP0232 while the NCGR 'nt' assembly had 4234 and 'cds' had 2736. BUSCO did not recognize any of the DIB contigs but it did recognize the NCGR contigs. Whereas for other samples, e.g. MMETSP0169, the BUSCO software recognized several contigs but did not score them high enough and called the BUSCO group "missing", even though there were lengths of contigs identified as being somewhat related.

This is a case where it does suggest several contigs are matching in DIB hmmer output, even though it is listed as "Missing":

MMETSP0169:

```
EOG0937060I
DIB: ['EOG0937060I', 'Missing']
NCGR: ['EOG0937060I', 'Duplicated', 'CAMNT_0039020233', '576.3', '630']
NCGR: ['EOG0937060I', 'Duplicated', 'CAMNT_0039023937', '555.9', '644']
NCGR: ['EOG0937060I', 'Duplicated', 'CAMNT_0039030405', '552.2', '636']
DIB contig: MMETSP0169-figshare3840153v7-TRINITY_DN13758_c3_g2_i1_3
DIB contig length: 974
DIB contig: MMETSP0169-figshare3840153v7-TRINITY_DN3716_c0_g1_i1_1
DIB contig length: 154
DIB contig: MMETSP0169-figshare3840153v7-TRINITY_DN13467_c1_g1_i1_6
DIB contig length: 494
NCGR contig: CAMNT_0039023937_4
NCGR contig length: 781
NCGR contig: CAMNT_0039030405_5
```

NCGR contig length: 921
NCGR contig: CAMNT_0039020233_3
NCGR contig length: 842
```
The gene is:
```
DNA/RNA helicase, ATP-dependent, DEAH-box type, conserved site
Similarity:Contains helicase ATP-binding domain
```
When we look for this gene in the annotation file, there are no annotation results for this contig:
```
[ljcohen@dev-intel14 MMETSP0169.trinity_out_2.2.0.Trinity.fasta.dammit]$ grep "TRINITY_DN13758_c3_g2_i1" MMETSP0169.trinity_out_2.2.0.Trinity.fasta.dammit.namemap.csv
```
But there are for other contigs identified by the hmm_output file:
```
[ljcohen@dev-intel14 MMETSP0169.trinity_out_2.2.0.Trinity.fasta.dammit]$ grep "TRINITY_DN3716_c0_g1_i1" MMETSP0169.trinity_out_2.2.0.Trinity.fasta.dammit.namemap.csv
"TRINITY_DN3716_c0_g1_i1 len=208 path=[186:0-207] [-1, 186, -2]",Transcript_32368

[ljcohen@dev-intel14 MMETSP0169.trinity_out_2.2.0.Trinity.fasta.dammit]$ grep "Transcript_32368" MMETSP0169.trinity_out_2.2.0.Trinity.fasta.dammit.gff3
Transcript_32368 LAST translated_nucleotide_match 15 167 1.300000e-06 - . ID=homology:18169;Name=ENSXMAP00000012674;Target=ENSXMAP00000012674 244 295 +;database=OrthoDB
```
Looking at the gene identified by the annotations:
```
[ljcohen@dev-intel14 reference]$ grep "ENSXMAP00000012674" ODB8_EukOGs_genes_ALL_levels.txt
7898:Actinopterygii EOG808R8M ENSXMAP00000012674 8083:003153 Xiphophorus maculatus
33208:Metazoa EOG82JQ5D ENSXMAP00000012674 8083:003153 Xiphophorus maculatus
1261581:Vertebrata EOG808P6W ENSXMAP00000012674 8083:003153 Xiphophorus maculatus

[ljcohen@dev-intel14 reference]$ grep "EOG808R8M" odb_v8_v9_1.tab
7898 EOG808R8M EOG090C08EI .39456585
```
This does not match the original EOG0937060I and is a different gene:
```
Abl-interactor, homeo-domain homologous domain
ABI family, member 3a

```

But, the top ncbi blastn results with the contig sequence suggested small (only several hundred bp) region matches with the EOG0937060I gene sequence:
```
PREDICTED: Heterocephalus glaber DEAH-box helicase 37 (Dhx37), transcript variant X2, mRNA 66.2 66.2 1% 6e-06 86% XM_021257656.1
Select seq XM_004843976.2 PREDICTED: Heterocephalus glaber DEAH-box helicase 37 (Dhx37), transcript variant X1, mRNA 66.2 66.2 1% 6e-06 86% XM_004843976.2
Select seq XM_010604294.2 PREDICTED: Fukomys damarensis DEAH-box helicase 37 (Dhx37), transcript variant X6, mRNA 66.2 66.2 1% 6e-06 86% XM_010604294.2
Select seq XM_010604293.2 PREDICTED: Fukomys damarensis DEAH-box helicase 37 (Dhx37), transcript variant X5, mRNA 66.2 66.2 1% 6e-06 86% XM_010604293.2
Select seq XM_010604292.2 PREDICTED: Fukomys damarensis DEAH-box helicase 37 (Dhx37), transcript variant X4, mRNA 66.2 66.2 1% 6e-06 86% XM_010604292.2
Select seq XM_010604291.2 PREDICTED: Fukomys damarensis DEAH-box helicase 37 (Dhx37), transcript variant X3, mRNA 66.2 66.2 1% 6e-06 86% XM_010604291.2
Select seq XR_776390.2 PREDICTED: Fukomys damarensis DEAH-box helicase 37 (Dhx37), transcript variant X2, misc_RNA 66.2 66.2 1% 6e-06 86% XR_776390.2
Select seq XM_019204571.1 PREDICTED: Fukomys damarensis DEAH-box helicase 37 (Dhx37), transcript variant X1, mRNA 66.2 66.2 1% 6e-06 86% XM_019204571.1
Select seq XM_005403001.2 PREDICTED: Chinchilla lanigera DEAH (Asp-Glu-Ala-His) box polypeptide 37 (Dhx37), mRNA
```

Even though this contig was assembled, it did not successfully annotate. We don't know whether there are errors associated with this assembled contig, or if the contig is a new sequence unique to this MMETSP0169 organism (*Corethron pennatum*, Phylum: Bacillariophyta). Since the BUSCO database and corresponding orthogroups were contstructed from multiple sequence alignments with inidividuals already in the databases, it is possible that different organisms have evolved slightly different sequences that may fall outside the hmm scoring cutoffs for matching with the BUSCO orthogroup. Since the corresponding NCGR assembly had a "Duplicated" result from this particular BUSCO, it is possible that there is a particular oddity within this ortholog.

This is an example where there is not a file in the DIB `run_MMETSP0169/hmmer_output` correspondint to this orthogroup EOG.

```
EOG0937017X
DIB: ['EOG0937017X', 'Missing']
NCGR: ['EOG0937017X', 'Complete', 'CAMNT_0039023621', '802.2', '1208']
NCGR contig: CAMNT_0039023621_3
NCGR contig length: 1352
NCGR contig: CAMNT_0039027673_4
NCGR contig length: 1287

NCGR contig: CAMNT_0039044891_4
NCGR contig length: 1358
```

Sifting through BUSCO output, there are more examples that could be picked over to explore this issue with these eight MMETSP samples with 30% difference between NCGR and DIB:

https://github.com/ljcohen/MMETSP/blob/master/assembly_evaluation_data/busco_eval/busco_eval.ipynb

For now, we conclude that our assemblies are differently fragmented in some regions relative to the NCGR assemblies. We have assembled additional sequences that were not assembled by NCGR. Some NCGR assemblies had different and more complete content than the DIB assemblies. As far as we can tell, there does not appear to be a pattern in the samples that fared well with this pipeline vs. NCGR. This could be a future avenue to explore.

Could you (did you) do a CRHB against Swiss-prot? I imagine that for each assembly pair (old assembly vs new assembly), you'd see more hits to unique Swiss-Prot genes in the newer assembly.

The dammit pipeline we used did perform a CRBB with Pfam, Rfam, Orthodb. A CRBB directly with Swiss-prot would probably not give additional information since Pfam is a collection of protein family alignments constructed using HMM of multiple Swiss-prot sequence alignments. Directly querying Swiss-prot might be an interesting avenue to pursue in the future to confirm annotations with Pfam, R-fam and Orthodb with this high quality annotated and non-redundant protein sequence database. But at this point it might be redundant and more noisy given the biodiversity and evolutionary divergence among the species found in the MMETSP collection.

L254: "less significantly different" Do you mean "significantly less"?

The difference in BUSCO was significant, but to a lesser degree than other distribution comparisons ($p=0.002$ rather than $p < 0.001$). I edited the wording here, see comment above.

L306: I'm also confused about the TransRate scores. As best as I can tell the "NT" assemblies were the raw assemblies, while the "CDS" assemblies were further filtered. If my understanding is correct, then the opening statement for this paragraph (DIB assemblies were more inclusive) is incorrect, given that transrate metrics were higher for the NCGR nt assemblies that they were for the DIB assemblies. I'm also worried about the statements about DIB assemblies being better, while transrate scores were on whole, worse. Should reconcile this.

Thank you for pointing out the imprecise language in our explanation. Yes, the "nt" assemblies were raw assemblies, whereas the "cds" assemblies that the NCGR published were filtered for
```

only coding sequences, as far as we can tell. (Methods described by Keeling et al. 2014 were not transparent enough to correspond to the file nomenclature located on ftp://ftp.imicrobe.us/projects/104/.) Transrate scores may not be the best metric for comparison. The transrate score is a measurement of how well the original reads support the the final assembly. We have more unique k-mers, but we have worse transrate scores.

We edited "suggesting that both pipelines yielded equally valid contigs," to "suggesting that both pipelines yielded similarly valid contigs," so that the significant BUSCO and ORF differences between assemblies can be acknowledged.

To the discussion, we added:
""
Moreover, even though the number of contigs and the CRBB results between the DIB and NCGR assemblies were dramatically different, both the fraction of contigs with ORFs and the mean percentage of BUSCO matches were similar between the two assemblies, suggesting that both pipelines yielded similarly valid contigs, even though the NCGR assemblies were less sensitive.
""

L315: I'm not sure that you are "directly" evaluating the de Bruijn structure.

This is correct. We did not directly evaluate the de Bruijn graph structure. The sentence in the discussion mentions:

Metrics directly evaluating the underlying de Bruijn graph data structure used to produce the assembled contigs may be better evaluators of assembly quality.

We clarified this by adding:

In future studies, metrics directly evaluating the underlying de Bruijn graph data structure used to produce the assembled contigs may be better evaluators of assembly quality in the future.

L320: I'm not sure you show "Biologically meaningful". You show that you have recovered new stuff that is likely real (not an artifact of asembly), but not sure you can claim it's meaningful.

This is true. We changed the word "meaningful" to "relevant".

L364: In your discussion of kmer content (and other metrics) the idea that some of these datasets might in fact be meta-transcriptomes should be discussed. Lots of marine microeukaryotes associate with bacteria, viruses, etc, and unless extreme care was takes with the target species, to grow in sterile conditions, some of patterns of kmer distrib. might be because the datasets contain more than 1 species.

We added a paragraph:

""
RNA sequences generated from the MMETSP experiments are likely to contain genetic information from more than the target species, as many were not or could not be cultured axenically. Thus both the NCGR assemblies and DIB re-assemblies, including the additional biologically relevant information, might be considered meta-transcriptomes. Sequencing data and unique k-mer content likely include bacteria, viruses, or other protists that occurred within the sequenced sample. We did not make an attempt to de-contaminate the assemblies.
""

Table 1. Can you include the BUSCO results here?

Yes, these were added.

Fig3 needs a y-axis label

Done.

Fig 5c and a few other places. There are a few DIB assemblies that are WAY worse than the original assembly. Why? This could benefit from some explanation.

Added to Discussion:

""
For some samples, the DIB re-assemblies had lower metrics than the NCGR assemblies. Complete BUSCO scores were lower than over half of DIB vs. NCGR. This could be an effect of the BUSCO metric, given that these samples did not perform poorly with other metrics, such as \% ORF and number of contigs compared to the NCGR. For other samples, MMETSP0252 (*Prorocentrum lima*) in particular, assemblies required several tries and only four contigs were assembled from 30 million reads of data. The fastqc reports were unremarkable compared to the other samples. In such a large dataset with a diversity of species with no prior sequencing data to compare make it challenging to speculate why each anomaly occurred. However, further investigation into the reasons for failures and peculiarities in the evaluation metrics may lead to interesting discoveries about how we should be effectively assembling and evaluating nucleic acid sequencing data from a diversity of species.
""

In the end, what I took away from this paper is that the new assemblies had different transcripts, and this is great and potentially helpful for researchers. Saying that, on a whole, both BUSCO and TransRate scores trended toward lower, which is maybe surprising, especially because the original assemblies were assembled (best as I can tell) using a general genome assembler (ABySS/MIRA) rather than software specialized for transcriptomes.

This is a good summary of our findings, thank you. We added a sentence to the Introduction mentioning the ABySS assembler and cited Keeling et al. 2014 for methods (LINE 78 in the formatted manuscript).

Reviewer #2: The manuscript by Johnson et al. describe the re-analysis of the Marine Microbial Eukaryotic Transcriptome Sequencing Project (MMETSP). The authors have generate a new computational pipeline for the de novo assembly (using Trinity de novo) of the RNA-Seq reads of several hundred transcriptomes as well as downstream a set of scripts to compare the outcome with the results of the original publication (which used Trans-ABySS for the assembly).
The current manuscript is a great example that shows the value of revisiting old data sets with new computational tools. The authors put strong focus on reproducibility of their analysis. The effort for this should not be underestimated and the work can serve as a blueprint for similar data re-analysis projects.
I see no major issue in this work but still would like to have a few smaller ones addressed:

We thank the reviewer for their compliments on our work and appreciate the feedback they have provided. Below please find our responses to the the detailed comments:

* The manuscript is currently rather descriptive and has only a few explanations why there are certain differences in the presented assembly approaches. E.g. what are the reasons for the observation displayed in Figure 4 that there so many more unique k-mers in the DIB than in the NCGR set? Maybe not all results can be explained mechanistically but least at some potential reasons could be discussed.

Thank you for pointing this out-- we have tried to expand our explanations where we felt confident in our reasoning. For example, we have added the following explanation for why greater kmer content is conserved by the DIB assembly:
Added to L316

""
The relative increase in number of unique k-mers from the NCGR assemblies to the DIB re-assemblies could be an effect of having more contigs. Within the data, the Trinity assembler found evidence for building alternative isoforms. Whereas the ABySS assembler and transcriptome pipeline that NCGR used may not have preserved that variation, in an attempt to narrow down the contigs to a consensus transcript sequence.
""

* The authors write: "We used a different pipeline than the original one used to create the NCGR assemblies, in part because new software was available [8] and in part because of new trimming guidelines [27]". Is [8] really the correct reference here? If so this has to be further explained.

Thank you for catching this. This was a typo. Fixed to reference [18].

* I think figures 2, 3 and 5 are not red green blind safe.

You are correct-- thank you for bringing this to our attention. We have changed the figures to blue and yellow.

* In the script collection uploaded to Zenodo I personally would have removed the "pycache" folder and the containing Python byte code files (*pyc). Or do they have any purpose / contain useful information?

Thank you, we will make sure to remove this before publication.

* The supplementary notebooks could additionally be uploaded as ipyn files.

Thank you for the suggestion. We have uploaded the notebooks both as ipynb files as static html pages to a figshare: https://doi.org/10.6084/m9.figshare.7091003

* The authors have a configuration file for user specif paths but this is not strictly used. In "dibMMETSPconfiguration.py" another "basedir" variable is set and in trimqc.py even the full path for Trimmomatic is set ("/mnt/home/ljcohen/bin/Trimmomatic-0.33/trimmomatic-0.33.jar"). This make the reuse of the framework harder.

Thank you for catching this, we have removed absolute paths and added the edited the files to a new version of the repository on zenodo. While we agree that it does make sense for future reuse, we felt it was important to preserve the code in its original state used to generate the assembly files discussed in the paper.

* While I understand that it is sometime needed due to dependencies on old libraries I would like to discourage the use of Python 2.7 (aka "legacy Python") in currently research projects and would strongly recommend to use a current Python version (3 and higher) instead.

We completely agree. At the time the scripts were written they were in Python 2.7. The assemblies discussed in the paper were generated with this code. Future versions of this pipeline are written in Python 3.

Reviewer #3:
In the manuscript, Johnson et al have reassembled RNA-seq data from 678 samples generated from MMETS Project using a pipeline, which follows the Eal Pond mRNA seq protocol. The pipeline (DIG) starts by quality trimming the data followed by digital normalization and assembly using the Trinity assembler. The authors have compared their re-assemblies against assemblies generated from the method suggested by the National Center for Genome Resource (NCGR). For comparison, they have used difference evaluation metrics like Conditional Reverse Best BLAST (CRBB), BUSCO scores, annotation using the Dammit pipeline and ORF content in the assembly. They argued that their pipeline is able to provide additional biologically meaningful content as compared to the NCGR pipeline. While the work overall is quite interesting and the large set of assemblies appear useful, I feel that there are some improvements and clarifications necessary:
Major comments:
1) The core reason behind the observation that DIG pipeline being better than the NCGR pipeline is not clear. It might be due to the core algorithm behind the assembler used by the pipelines (DIG using Trinity and NCGR uses AbySS). But this should be explained in more

detail why their pipeline performs better. For example, is the performance increase linked to sequencing coverage of the read data sets? Or transcriptome complexity of the sample? Or is it the fact that the NCGR pipeline seems to use a custom build pipeline that uses multi-kmer ABySS but not the de novo transcriptome assembler trans-ABySS, which may be more suited?

Thank you for bringing this to our attention. We agree that we did not discuss this point thoroughly enough in the text. The main differences between the NCGR and DIB pipelines were the assemblers used (we used Trinity) and trimming parameters (MacManes 2014). Additionally, in postprocessing, we did not filter out assembly contigs for ORF content whereas the NCGR did in their 'cds' versions.**

We have added the following text to the discussion that explains that we believe the reason for improved metrics with the DIB pipeline relative to the NCGR could be because of increased contigs from the Trinity assembly and trimming parameters used.

""
The increase in number of unique k-mers from the NCGR assemblies to the DIB re-assemblies could due to the higher number of contigs generated by Trinity. Within the data, the Trinity assembler found evidence for building alternative isoforms. Whereas the ABySS assembler and transcriptome pipeline that NCGR used \citep{Keeling_2014} appears to not have preserved that variation, in an attempt to narrow down the contigs to a consensus transcript sequence.
""

2) The other major difference between the pipelines is the additional step of digital normalization which DIG uses. Normalization generally removes kmer information, which affect the overall assembly. It is not clear why normalization in case of DIG should improve the assemblies. Normally the expectation would not that the digital normalization leads to an improvement. So I assume the authors do it simply to reduce the computational costs of the many assemblies, which is plausible but should be stated.

Yes, our DIB pipeline used digital normalization with khmer, which is much like Trinity normalization, to remove redundant k-mers for the purpose of reducing computational resources required. As Brown et al (2012) showed, digital normalization does not affect the content of the resulting transcriptome. We clarified on line 144: "To decrease the memory requirements for each assembly, digital normalization was applied prior to assembly [46]."

Also, Trinity by default performs in-silico normalization. So, the additional normalization step is redundant. Is the option for normalization switched off in the assembler. If yes, the authors should comment on why they are using Diginorm instead of using Trinity's built-in normalization, is there any indication that this works better for the assemblies they have done?

We used an older version of Trinity (2.2.0) which did not have this turned on by default. We added: "This version of Trinity (2.2.0) did not include the “in silico normalization” option as a default parameter.

The digital normalization used here is the same algorithm as the Trinity in silico normalization, but it requires considerably less memory and is faster (Brown et al. 2012).

3) It is not clear which version of NCGR assemblies ("nt" or "cds") the authors used for calculating the mean ORF% in Table 1. If they have used the "nt" version, then the number can be misleading. The "cds" version of the NCGR assemblies contains contigs that have been predicted to show coding potential and hence might have a higher mean ORF content (as this is computed as percentages). I suggest the authors compare the mean ORF% content of the two NCGR version against the assemblies generated using DIG for full transparency and then discuss the differences regarding these two NCGR version and their assemblies.

Thank you for pointing this out. We have clarifed clarified the mean % ORF metric in Table 1, which originally only included the "nt" version of the NCGR assembly, and added the assembly numbers corresponding to the "cds" version of the NCGR assembly. The DIB re-assemblies were more comparable to the "nt" versions of NCGR since we did not filter contigs based on ORF content, which the NCGR did in their "cds" version. When filtration steps were performed, potentially useful content was lost. Explanations clarifying the comparability of the DIB to the "nt" and the "cds" versions of the NCGR assemblies were added to the results and discsussion.

4) I think the line plots used in the paper can be improved, because it is hard to quantify the amount of overlapping lines. For example I think that Figure 2A, 3A,5A,5C are probably more easy to interpret when made as a scatterplot, e.g. Fig2A where the number of contigs is compared between NCGR and DIB assemblies.

We appreciate this feedback; however, we feel that in this context, the line plots are visually drawing the relationship between the same sample for each NCGR and DIB assembly. Scatter plots would not show that relationship. We attempted to clarify this point by adding this sentence (Figure 2, line 151 in the formatted manuscript)

""
Slopegraphs show shifts in the number of contigs for each individual sample between the DIB and the NCGR assembly pipelines. Negative slope (brown) lines represent values where NCGR was higher than DIB and positive slope (blue) lines represent values where DIB was higher than NCGR.
""

5) I would not say that the distribution in Figure 2c looks like a Normal distribution as the right tail is much heavier than the left one. If you want to make that statement, use a test of normality, however I feel this is not important for the paper.

Changed to:

""
The frequency of the differences between Transrate scores in the NCGR ‘nt’ assemblies and the

DIB re-assemblies is centered around zero (Figure 2C).
""

Minor comments:
-Typo in reference 25 .. de ovo assembly ..
Thank you for catching this typo. We have fixed it in the references.

-line 336: I was not able to understand what the (see op-ed Alexander et al. 2018) refers to, as there is no such reference in the bibliography and no footnote
We were not able to post to this article to a preprint server as it was not original research. Here is a link to the current github repo for the text of the citation: https://github.com/dib-lab/2018-paper-reanalysis-op-ed

The final version of this paper will include a proper citation.

Close
