# Supplementary material for: Re-assembly, quality evaluation, and annotation of 678 microbial eukaryotic reference transcriptomes
Source: Gigascience. 2018 Dec 13;8(4):giy158. doi: 10.1093/gigascience/giy158 (PMC6481552; doi:10.1093/gigascience/giy158)
Supplement: Response-to-Reviewer-Comments_Revision-1.pdf [file giy158_response-to-reviewer-comments_revision-1.pdf]

Author's Response To Reviewer Comments

Close

Minor changes as requested.

Close
