# Supplementary material for: Re-assembly, quality evaluation, and annotation of 678 microbial eukaryotic reference transcriptomes
Source: Gigascience. 2018 Dec 13;8(4):giy158. doi: 10.1093/gigascience/giy158 (PMC6481552; doi:10.1093/gigascience/giy158)
Supplement: Reviewer-1-Report-(Original-Submission) -- Matthew Macmanes — 6/24/2018 Reviewed [file giy158_reviewer-1-report-(original-submission).pdf]

Reviewer Report

Title: Re-assembly, quality evaluation, and annotation of 678 microbial eukaryotic reference transcriptomes

Version: Original Submission **Date:** 6/24/2018

Reviewer name: Matthew Macmanes

Reviewer Comments to Author:

The manuscript submitted by Lisa Johnson and colleagues is a well written and comprehensive work aimed at reanalyzing a fairly enormous dataset. I feel that the work is important for the following two main reasons:

1. Assembly methods have improved substantially since the original datasets were analyzed, and as the authors point out - these new analyses recover new transcripts that might be useful to the original researchers and to the broader community.

2. Applying standardized and reproducible methods - at scale - is challenging, and the authors provide an example for how this could be done. I can imagine others using these ideas (or the actual code) to assemble other datasets in a similar fashion.

In terms of the manuscript itself, it is sound, with just a few areas where improvements will make for a more readable paper. Interspersed with this, I have a few more pedantic suggestions that the author should feel free to ignore if deemed unhelpful.

Line 91: replace 'higher' with 'more favorable' or even 'better'
L102: The link to the code does not seem to be active. I would have loved to review it.
L111: You are using 50bp reads. Do you think your conclusions would have been any different had longer reads (100-150bp) been available? More novice readers might wonder if these methods are just as applicable to them with longer reads as they are to you. I'm sure the answer is yes - your new assemblies might have been even better had you had longer reads.
L180-182: If I didn't know khmer already, I might struggle with the HyperLogLog estimator. Maybe just a sentence or 3 more might be useful to explain what this is and why it is used.

L245ish: I keep wondering about your BUSCO scores, and the fact tat they are lower on average in your new assemblies compared to the older ones. Why is this? How do you reconcile this with the more general statement you are trying to make about 'more genes being recovered' in the new assembly. I see that BUSCO is just one of the available metrics to assess this, but it's a little strange I think, given that I'm convinced that these assemblies are actually better.

Could you (did you) do a CRHB against Swiss-prot? I imagine that for each assembly pair (old assembly vs

new assembly), you'd see more hits to unique Swiss-Prot genes in the newer assembly.

L254: "less significantly different" Do you mean "significantly less"?

L306: I'm also confused about the TransRate scores. As best as I can tell the "NT" assemblies were the raw assemblies, while the "CDS" assemblies were further filtered. If my understanding is correct, then the opening statement for this paragraph (DIB assemblies were more inclusive) is incorrect, given that transrate metrics were higher for the NCGR nt assemblies that they were for the DIB assemblies. I'm also worried about the statements about DIB assemblies being better, while transrate scores were on whole, worse. Should reconcile this.

L315: I'm not sure that you are "directly" evaluating the de Bruijn structure.

L320: I'm not sure you show "Biologically meaningful". You show that you have recovered new stuff that is likely real (not an artifact of asembly), but not sure you can claim it's meaningful.

L364: In your discussion of kmer content (and other metrics) the idea that some of these datasets might in fact be meta-transcriptomes should be discussed. Lots of marine microeukaryotes associate with bacteria, viruses, etc, and unless extreme care was takes with the target species, to grow in sterile conditions, some of patterns of kmer distrib. might be because the datasets contain more than 1 species.

Table 1. Can you include the BUSCO results here?
Fig3 needs a y-axis label
Fig 5c and a few other places. There are a few DIB assemblies that are WAY worse than the original assembly. Why? This could benefit from some explanation.

In the end, what I took away from this paper is that the new assemblies had different transcripts, and this is great and potentially helpful for researchers. Saying that, on a whole, both BUSCO and TransRate scores trended toward lower, which is maybe surprising, especially because the original assemblies were assembled (best as I can tell) using a general genome assembler (ABySS/MIRA) rather than software specialized for transcriptomes.

Methods

Are the methods appropriate to the aims of the study, are they well described, and are necessary controls included? Choose an item.

Conclusions

Are the conclusions adequately supported by the data shown? Choose an item.

Reporting Standards

Does the manuscript adhere to the journal's guidelines on [minimum standards of reporting?] Choose an item.

Choose an item.

Statistics

Are you able to assess all statistics in the manuscript, including the appropriateness of statistical tests used? Choose an item.

Quality of Written English

Please indicate the quality of language in the manuscript: Choose an item.

Declaration of Competing Interests

Please complete a declaration of competing interests, considering the following questions:

- Have you in the past five years received reimbursements, fees, funding, or salary from an organisation that may in any way gain or lose financially from the publication of this manuscript, either now or in the future?
- Do you hold any stocks or shares in an organisation that may in any way gain or lose financially from the publication of this manuscript, either now or in the future?
- Do you hold or are you currently applying for any patents relating to the content of the manuscript?
- Have you received reimbursements, fees, funding, or salary from an organization that holds or has applied for patents relating to the content of the manuscript?
- Do you have any other financial competing interests?
- Do you have any non-financial competing interests in relation to this paper?

If you can answer no to all of the above, write 'I declare that I have no competing interests' below. If your reply is yes to any, please give details below.

I declare that I have no competing interests

I agree to the open peer review policy of the journal. I understand that my name will be included on my report to the authors and, if the manuscript is accepted for publication, my named report including any attachments I upload will be posted on the website along with the authors' responses. I agree for my report to be made available under an Open Access Creative Commons CC-BY license (http://creativecommons.org/licenses/by/4.0/). I understand that any comments which I do not wish to

be included in my named report can be included as confidential comments to the editors, which will not be published.

I agree to the open peer review policy of the journal

To further support our reviewers, we have joined with Publons, where you can gain additional credit to further highlight your hard work (see: https://publons.com/journal/530/gigascience). On publication of this paper, your review will be automatically added to Publons, you can then choose whether or not to claim your Publons credit. I understand this statement.

Yes Choose an item.
