# Supplementary material for: Re-assembly, quality evaluation, and annotation of 678 microbial eukaryotic reference transcriptomes
Source: Gigascience. 2018 Dec 13;8(4):giy158. doi: 10.1093/gigascience/giy158 (PMC6481552; doi:10.1093/gigascience/giy158)
Supplement: Reviewer-2-Report-(Original-Submission) -- Konrad Foerstner — 7/2/2018 Reviewed [file giy158_reviewer-2-report-(original-submission).pdf]

Reviewer Report

Title: Re-assembly, quality evaluation, and annotation of 678 microbial eukaryotic reference transcriptomes

Version: Original Submission **Date:** 7/2/2018

Reviewer name: Konrad Foerstner

Reviewer Comments to Author:

The manuscript by Johnson et al. describe the re-analysis of the Marine Microbial Eukaryotic Transcriptome Sequencing Project (MMETSP). The authors have generate a new computational pipeline for the de novo assembly (using Trinity de novo) of the RNA-Seq reads of several hundred transcriptomes as well as downstream a set of scripts to compare the outcome with the results of the original publication (which used Trans-ABySS for the assembly).

The current manuscript is a great example that shows the value of revisiting old data sets with new computational tools. The authors put strong focus on reproducibility of their analysis. The effort for this should not be underestimated and the work can serve as a blueprint for similar data re-analysis projects.

I see no major issue in this work but still would like to have a few smaller ones addressed:

* The manuscript is currently rather descriptive and has only a few explanations why there are certain differences in the presented assembly approaches. E.g. what are the reasons for the observation displayed in Figure 4 that there so many more unique k-mers in the DIB than in the NCGR set? Maybe not all results can be explained mechanistically but least at some potential reasons could be discussed.
* The authors write: "We used a different pipeline than the original one used to create the NCGR assemblies, in part because new software was available [8] and in part because of new trimming guidelines [27]". Is [8] really the correct reference here? If so this has to be further explained.
* I think figures 2, 3 and 5 are not red green blind safe.
* In the script collection uploaded to Zenodo I personally would have removed the "pycache" folder and the containing Python byte code files (*pyc). Or do they have any purpose / contain useful information?
* The supplementary notebooks could additionally be uploaded as ipyn files.
* The authors have a configuration file for user specif paths but this is not strictly used. In "dibMMETSPconfiguration.py" another "basedir" variable is set and in trimqc.py even the full path for Trimmomatic is set ("/mnt/home/ljcohen/bin/Trimmomatic-0.33/trimmomatic-0.33.jar"). This make the reuse of the framework harder.
* While I understand that it is sometime needed due to dependencies on old libraries I would like to discourage the use of Python 2.7 (aka "legacy Python") in currently research projects and would strongly recommend to use a current Python version (3 and higher) instead.

Methods

Are the methods appropriate to the aims of the study, are they well described, and are necessary controls included? Choose an item.

Conclusions

Are the conclusions adequately supported by the data shown? Choose an item.

Reporting Standards

Does the manuscript adhere to the journal's guidelines on minimum standards of reporting? Choose an item.

Choose an item.

Statistics

Are you able to assess all statistics in the manuscript, including the appropriateness of statistical tests used? Choose an item.

Quality of Written English

Please indicate the quality of language in the manuscript: Choose an item.

Declaration of Competing Interests

Please complete a declaration of competing interests, considering the following questions:

- Have you in the past five years received reimbursements, fees, funding, or salary from an organisation that may in any way gain or lose financially from the publication of this manuscript, either now or in the future?
- Do you hold any stocks or shares in an organisation that may in any way gain or lose financially from the publication of this manuscript, either now or in the future?
- Do you hold or are you currently applying for any patents relating to the content of the manuscript?
- Have you received reimbursements, fees, funding, or salary from an organization that holds or has applied for patents relating to the content of the manuscript?
- Do you have any other financial competing interests?
- Do you have any non-financial competing interests in relation to this paper?

If you can answer no to all of the above, write 'I declare that I have no competing interests' below. If your reply is yes to any, please give details below.

I declare that I have no competing interests

I agree to the open peer review policy of the journal. I understand that my name will be included on my report to the authors and, if the manuscript is accepted for publication, my named report including any attachments I upload will be posted on the website along with the authors' responses. I agree for my report to be made available under an Open Access Creative Commons CC-BY license (http://creativecommons.org/licenses/by/4.0/). I understand that any comments which I do not wish to be included in my named report can be included as confidential comments to the editors, which will not be published.

I agree to the open peer review policy of the journal

To further support our reviewers, we have joined with Publons, where you can gain additional credit to further highlight your hard work (see: https://publons.com/journal/530/gigascience). On publication of this paper, your review will be automatically added to Publons, you can then choose whether or not to claim your Publons credit. I understand this statement.

Yes Choose an item.
