# Supplementary material for: Re-assembly, quality evaluation, and annotation of 678 microbial eukaryotic reference transcriptomes
Source: Gigascience. 2018 Dec 13;8(4):giy158. doi: 10.1093/gigascience/giy158 (PMC6481552; doi:10.1093/gigascience/giy158)
Supplement: Reviewer-3-Report-(Original-Submission) -- Marcel Schulz — 7/4/2018 Reviewed [file giy158_reviewer-3-report-(original-submission).pdf]

Reviewer Report

Title: Re-assembly, quality evaluation, and annotation of 678 microbial eukaryotic reference transcriptomes

Version: Original Submission **Date:** 7/4/2018

Reviewer name: Marcel Schulz

Reviewer Comments to Author:

In the manuscript, Johnson et al have reassembled RNA-seq data from 678 samples generated from MMETS Project using a pipeline, which follows the Eal Pond mRNA seq protocol. The pipeline (DIG) starts by quality trimming the data followed by digital normalization and assembly using the Trinity assembler. The authors have compared their re-assemblies against assemblies generated from the method suggested by the National Center for Genome Resource (NCGR). For comparison, they have used difference evaluation metrics like Conditional Reverse Best BLAST (CRBB), BUSCO scores, annotation using the Dammit pipeline and ORF content in the assembly. They argued that their pipeline is able to provide additional biologically meaningful content as compared to the NCGR pipeline. While the work overall is quite interesting and the large set of assemblies appear useful, I feel that there are some improvements and clarifications necessary:

Major comments:

1) The core reason behind the observation that DIG pipeline being better than the NCGR pipeline is not clear. It might be due to the core algorithm behind the assembler used by the pipelines (DIG using Trinity and NCGR uses AbySS). But this should be explained in more detail why their pipeline performs better. For example, is the performance increase linked to sequencing coverage of the read data sets? Or transcriptome complexity of the sample? Or is it the fact that the NCGR pipeline seems to use a custom build pipeline that uses multi-kmer ABySS but not the de novo transcriptome assembler trans-ABySS, which may be more suited?

2) The other major difference between the pipelines is the additional step of digital normalization which DIG uses. Normalization generally removes kmer information, which affect the overall assembly. It is not clear why normalization in case of DIG should improve the assemblies. Normally the expectation would not that the digital normalization leads to an improvement. So I assume the authors do it simply to reduce the computational costs of the many assemblies, which is plausible but should be stated. Also, Trinity by default performs in-silico normalization. So, the additional normalization step is redundant. Is the option for normalization switched off in the assembler. If yes, the authors should comment on why they are using Diginorm instead of using Trinity's built-in normalization, is there any indication that this works better for the assemblies they have done?

3) It is not clear which version of NCGR assemblies ("nt" or "cds") the authors used for calculating the

mean ORF% in Table 1. If they have used the "nt" version, then the number can be misleading. The "cds" version of the NCGR assemblies contains contigs that have been predicted to show coding potential and hence might have a higher mean ORF content (as this is computed as percentages). I suggest the authors compare the mean ORF% content of the two NCGR version against the assemblies generated using DIG for full transparency and then discuss the differences regarding these two NCGR version and their assemblies.

4) I think the line plots used in the paper can be improved, because it is hard to quantify the amount of overlapping lines. For example I think that Figure 2A, 3A,5A,5C are probably more easy to interpret when made as a scatterplot, e.g. Fig2A where the number of contigs is compared between NCGR and DIB assemblies.

5) I would not say that the distribution in Figure 2c looks like a Normal distribution as the right tail is much heavier than the left one. If you want to make that statement, use a test of normality, however I feel this is not important for the paper.

Minor comments:

-Typo in reference 25 .. de ovo assembly ..
-line 336: I was not able to understand what the (see op-ed Alexander et al. 2018) refers to, as there is no such reference in the bibliography and no footnote

Methods

Are the methods appropriate to the aims of the study, are they well described, and are necessary controls included? Choose an item.

Conclusions

Are the conclusions adequately supported by the data shown? Choose an item.

Reporting Standards

Does the manuscript adhere to the journal's guidelines on minimum standards of reporting? Choose an item.

Choose an item.

Statistics

Are you able to assess all statistics in the manuscript, including the appropriateness of statistical tests used? Choose an item.

Quality of Written English

Please indicate the quality of language in the manuscript: Choose an item.

Declaration of Competing Interests

Please complete a declaration of competing interests, considering the following questions:

- Have you in the past five years received reimbursements, fees, funding, or salary from an organisation that may in any way gain or lose financially from the publication of this manuscript, either now or in the future?
- Do you hold any stocks or shares in an organisation that may in any way gain or lose financially from the publication of this manuscript, either now or in the future?
- Do you hold or are you currently applying for any patents relating to the content of the manuscript?
- Have you received reimbursements, fees, funding, or salary from an organization that holds or has applied for patents relating to the content of the manuscript?
- Do you have any other financial competing interests?
- Do you have any non-financial competing interests in relation to this paper?

If you can answer no to all of the above, write 'I declare that I have no competing interests' below. If your reply is yes to any, please give details below.

i declare that i have no competing interests

I agree to the open peer review policy of the journal. I understand that my name will be included on my report to the authors and, if the manuscript is accepted for publication, my named report including any attachments I upload will be posted on the website along with the authors' responses. I agree for my report to be made available under an Open Access Creative Commons CC-BY license (http://creativecommons.org/licenses/by/4.0/). I understand that any comments which I do not wish to be included in my named report can be included as confidential comments to the editors, which will not be published.

I agree to the open peer review policy of the journal

To further support our reviewers, we have joined with Publons, where you can gain additional credit to further highlight your hard work (see: https://publons.com/journal/530/gigascience). On publication of this paper, your review will be automatically added to Publons, you can then choose whether or not to claim your Publons credit. I understand this statement.

Yes Choose an item.
